# The performance of FIT-based and other risk prediction models for colorectal neoplasia in symptomatic patients: a systematic review

**DOI:** 10.1016/j.eclinm.2023.102204

**Published:** 2023-09-21

**Authors:** James S. Hampton, Ryan P.W. Kenny, Colin J. Rees, William Hamilton, Claire Eastaugh, Catherine Richmond, Linda Sharp, COLOFIT Research Team

**Affiliations:** a Population Health Sciences Institute, Newcastle University, United Kingdom; b Department of Gastroenterology, South Tyneside and Sunderland NHS Foundation Trust, United Kingdom; c Evidence Synthesis Group, The Catalyst, Population Health Sciences Institute, Newcastle University, United Kingdom; d National Institute for Health and Care Research Innovation Observatory, The Catalyst, Newcastle University, United Kingdom; e College of Medicine and Health, University of Exeter, United Kingdom

**Keywords:** FIT, Colorectal cancer, Risk prediction models, Symptoms

## Abstract

**Background:**

Colorectal cancer (CRC) incidence and mortality are increasing internationally. Endoscopy services are under significant pressure with many overwhelmed. Faecal immunochemical testing (FIT) has been advocated to identify a high-risk population of symptomatic patients requiring definitive investigation by colonoscopy. Combining FIT with other factors in a risk prediction model could further improve performance in identifying those requiring investigation most urgently. We systematically reviewed performance of models predicting risk of CRC and/or advanced colorectal polyps (ACP) in symptomatic patients, with a particular focus on those models including FIT.

**Methods:**

The review protocol was published on PROSPERO (CRD42022314710). Searches were conducted from database inception to April 2023 in MEDLINE, EMBASE, Cochrane libraries, SCOPUS and CINAHL. Risk of bias of each study was assessed using The Prediction study Risk Of Bias Assessment Tool. A narrative synthesis based on the guidelines for Synthesis Without Meta-Analysis was performed due to study heterogeneity.

**Findings:**

We included 62 studies; 23 included FIT (n = 22) or guaiac Faecal Occult Blood Testing (n = 1) combined with one or more other variables. Twenty-one studies were conducted solely in primary care. Generally, prediction models including FIT consistently had good discriminatory ability for CRC/ACP (i.e. AUC >0.8) and performed better than models without FIT although some models without FIT also performed well. However, many studies did not present calibration and internal and external validation were limited. Two studies were rated as low risk of bias; neither model included FIT.

**Interpretation:**

Risk prediction models, including and not including FIT, show promise for identifying those most at risk of colorectal neoplasia. Substantial limitations in evidence remain, including heterogeneity, high risk of bias, and lack of external validation. Further evaluation in studies adhering to gold standard methodology, in appropriate populations, is required before widespread adoption in clinical practice.

**Funding:**

10.13039/501100000272National Institute for Health and Care Research (NIHR) [10.13039/501100000664Health Technology Assessment Programme (HTA) Programme (Project number 133852).

**Research in context**

**Evidence before this study**

Colonoscopy is an expensive and invasive investigation and health services cannot cope with demand. There is a widespread view that less invasive tools are required to determine which patients require colonoscopy. The use of faecal immunochemical testing (FIT) in the symptomatic setting has significantly increased over recent years and, in some settings, guidance now advocates FIT for use in patients with features of possible colorectal cancer (CRC) to guide referral for urgent investigation. There is growing interest in the use of risk prediction models–statistical models that combine information from two or more variables to predict the likelihood of an outcome, and whether these models could further improve performance in identifying those requiring investigation.

In this review we included studies assessing symptomatic patients, developing/validating a predictive model (with 2 or more factors) for the prediction of CRC and/or advanced colorectal polyp (ACP) using MEDLINE, EMBASE, Cochrane libraries, SCOPUS and CINAHL electronic databases from inception to April 2023.

**Added value of this study**

The review provides a comprehensive and up to date review on the ability of risk prediction models (FIT and non-FIT based) to identify colorectal neoplasia. It both updates and extends a past systematic review on this topic (which included papers published to March 2014) and evaluates the evidence in the context of current clinical practice.

**Implications of all the available evidence**

This review shows that there is considerable potential for the use of risk prediction models, both FIT-based and non-FIT based, in identifying those most at risk of colorectal neoplasia. However further evaluation of models is required in ‘real world’ settings before widespread use in clinical practice can be recommended. Based upon this review this team have undertaken research to develop risk models in the UK population that will be used to guide UK policy.

## Introduction

Colorectal cancer (CRC) is the third most common cancer and second most common cause of cancer death worldwide, accounting for 1.9 million new cases and 935,000 deaths in 2020.^1^ The incidence of CRC is increasing and it is predicted that, by 2040 the number of new CRC cases globally per year will reach 3.2 million.^2^ This rise is based on projections of population ageing, population growth and human development.^2^^,^^3^

Most CRCs develop from pre-cancerous colorectal lesions (adenomas or serrated polyps) progressing, if left *in situ*, to CRC.^4^^,^^5^ This natural history means that there is considerable opportunity for cancer prevention if pre-cancerous lesions can be detected early and removed. Whilst population-based screening is effective in reducing incidence and mortality,^6^ the overwhelming majority of CRCs are diagnosed after symptoms develop, such as a change in bowel habit, abdominal pain, weight loss or the presence of iron deficiency anaemia.^7^^,^^8^

Colonoscopy, by allowing direct visualisation of the colonic mucosa, is the preferred investigation for those with suspected CRC.^9^ However, patients can experience pain, discomfort or anxiety before, during or after the procedure, and there is a risk (albeit small) of significant complications including haemorrhage and perforation.^10^^,^^11^ Moreover, demand on endoscopy services is increasing. In the United Kingdom (UK), for example, less than three-quarters of services meet targets for prompt investigation of patients referred for urgent investigation of symptoms.^12^^,^^13^

Until recently, there was no test to identify those higher-risk symptomatic patients warranting colonoscopy, nor to determine the urgency of investigation. In recent years, driven by growing demand for colonoscopy, researchers and service providers have explored the utility of Faecal Immunochemical Testing (FIT) in symptomatic populations.^14^^,^^15^ FIT is simple, non-invasive, can be completed by the patient at home, and is relatively cheap, making it attractive for widespread use. There is evidence to suggest that FIT is powerful in identifying a high-risk sub-population when used in symptomatic patients.^14^ As a consequence, guidance has begun to advocate routine use of FIT in patients with features of possible CRC.^16^ Alongside this, interest has grown in the development of risk prediction models–statistical models that combine information from two or more variables to predict the likelihood of an outcome–which seek to identify which sub-groups of symptomatic patients (e.g. defined by FIT result and/or a combination of other factors such as age, sex or medical history) are most likely to have pre-cancerous lesions or CRC.^17^ The hope is that routine implementation of the algorithms in such models could provide an efficient way for health services to ensure that those patients most at risk undergo colonoscopy in a timely manner, while those at lowest risk avoid unnecessary procedures.^18^^,^^19^

The aim of this systematic review was to identify, and assess the performance of, models that predict the risk of CRC and/or advanced colorectal polyps (ACP) in symptomatic patients, with a particular focus on those models that include FIT.

## Methods

### Study design

The review was registered with the International Prospective Register of Systematic Reviews (PROSPERO) (CRD42022314710) (Supplementary File 1) and has been conducted and reported in line with the Preferred Reporting Items for Systematic Review and Meta-Analysis Protocols (PRISMA) statement.^20^

The eligibility criteria were developed using the PICOTS (Population, Intervention, Comparator, Outcome, Timing, Setting) framework^21^ (Supplementary File 1). We included studies assessing symptomatic patients, developing/validating a predictive model (with 2 or more factors) for the prediction of CRC and/or ACP (see Supplementary File 1 for further detail on definition/terms used for ACP; in brief we accepted as eligible studies, which used a range of different terms). Studies could be randomised trials or observational studies that were conducted in primary, secondary or tertiary care. Studies utilising primary care databases/cancer registries were included if they did not explicitly state the study population included asymptomatic (screening) individuals. The main outcome was model accuracy (e.g. AUC, sensitivity, specificity) but we also included studies reporting positive predictive values (PPV) for combinations of predictors. In a deviation from protocol, studies reporting PPV, which used age or sex in combination with one other factor were not considered predictive models, as these generally involved simply calculating PPV for strata of the study population based on demographics; however, studies reporting PPV which included age *and* sex *and* at least one other factor were eligible. Studies were also excluded if they were not in English; assessed screening or surveillance only populations or prognostic factors for treatment or outcome of CRC; focused only on genetic variables; or included paediatric populations.

Searches were conducted from database inception to 4th March 2022, and updated on the 28th April 2023, in MEDLINE, EMBASE, Cochrane libraries, SCOPUS and CINAHL. The search strategy was developed by an information specialist in combination with the review team, utilising a pre-existing prognostic study filter.^22^ The complete search strategy can be seen in Supplementary File 2. Additionally, forward and backward citation searching was conducted on all included studies and systematic reviews identified as being relevant.

Study selection was conducted in two stages, first screening citations and then full text of potentially eligible papers, using Rayyan^23^ by two reviewers (JSH & RPWK) independently. A third reviewer (LS) arbitrated any conflicts at both title and abstract and full text screening stages. A data extraction form based on CHecklist for critical Appraisal and data extraction for systematic Reviews of prediction Modelling Studies (CHARMS) was created and utilised.^24^ Data were extracted by a single reviewer (JSH or RPKW) and checked for accuracy by a second reviewer (JSH or RPKW). For further information of what data was extracted, please see Supplementary File 1. The Prediction study Risk Of Bias Assessment Tool (PROBAST) was used to assess the risk of bias.^25^ One reviewer (JSH or RPKW) assessed risk of bias, with the second reviewer (JSH or RPKW) checking for accuracy.

### Synthesis methods & statistical analysis

No statistical analyses were conducted due to heterogeneity of the studies, which meant a meta-analysis was not possible. We include forest plots for studies that report measures of discrimination (i.e. AUC) as a visual representation only. These forest plots do not include a summary of the effect size (weighted or unweighted) as computing these was not deemedstatistically appropriate. A narrative synthesis based on the guidelines for Synthesis Without Meta-analysis was therefore completed.^26^ For the purpose of synthesis, studies were categorised into FIT and non-FIT containing models. Where models included guaiac faecal occult blood testing (gFOBT) they were grouped with FIT containing models since both methods detect blood in stool to aid synthesis, where studies with binary outcomes reported a c-statistic, this has been referred to as AUC.

### Role of the funding source

The funders played no role in the study design, collection, analysis, and interpretation of data, nor the writing of the report or the decision to submit the paper for publication. JSH and RPWK accessed and verified the data. LS, CJR and WH made the decision to submit the manuscript for publication.

## Results

**Fig. 1:**
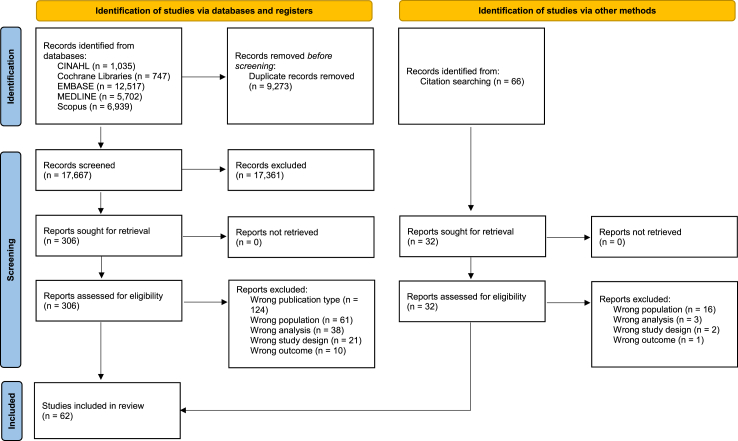
Study selection process.

Database searches, after de-duplication, provided 17,667 records for screening; 306 full text papers were assessed. Citation chaining provided a further 66 records; 32 were assessed at full text. The study selection process and reasons for exclusions are shown in Fig. 1. Overall, 62 studies were included in the review and synthesis. An overview of what each model contains can be seen in Supplementary File 3.

All included studies were of an observational study design, with 21 cross-sectional studies,^19^^,^^27^, ^28^, ^29^, ^30^, ^31^, ^32^, ^33^, ^34^, ^35^, ^36^, ^37^, ^38^, ^39^, ^40^, ^41^, ^42^, ^43^, ^44^, ^45^ 17 retrospective studies,^18^^,^^30^^,^^46^, ^47^, ^48^, ^49^, ^50^, ^51^, ^52^, ^53^, ^54^, ^55^, ^56^, ^57^, ^58^, ^59^, ^60^ 15 prospective studies,^39^^,^^61^, ^62^, ^63^, ^64^, ^65^, ^66^, ^67^, ^68^, ^69^, ^70^, ^71^, ^72^, ^73^, ^74^ and eight case–control studies.^62^^,^^75^, ^76^, ^77^, ^78^, ^79^, ^80^ One study design was unclear, as it was an abstract only.^81^

Settings were primary care (n = 21),^30^^,^^31^^,^^35^^,^^47^^,^^50^, ^51^, ^52^, ^53^, ^54^^,^^56^^,^^59^^,^^62^^,^^64^^,^^66^^,^^68^^,^^71^^,^^73^^,^^77^^,^^78^^,^^80^ primary and secondary care (n = 12),^18^^,^^19^^,^^30^^,^^33^^,^^34^^,^^49^^,^^60^^,^^63^^,^^65^^,^^67^^,^^72^ secondary care (n = 24),^29^^,^^32^^,^^36^, ^37^, ^38^, ^39^^,^^41^, ^42^, ^43^, ^44^^,^^46^^,^^48^^,^^55^^,^^57^^,^^58^^,^^61^^,^^62^^,^^69^^,^^70^^,^^74^, ^75^, ^76^^,^^79^^,^^82^ secondary and tertiary care (n = 3),^27^^,^^28^^,^^40^ and tertiary care (n = 1).^45^ One study was unclear regarding the setting.^81^ Databases or registries were used in 17 studies.^30^^,^^47^^,^^48^^,^^50^, ^51^, ^52^, ^53^, ^54^^,^^56^^,^^60^^,^^77^, ^78^, ^79^, ^80^^,^^82^, ^83^, ^84^

The studies were conducted in 15 different countries. One study examined patients from two different countries: Scotland and Spain.^18^ A further 24 studies assessed patients from the UK,^30^^,^^31^^,^^38^^,^^41^^,^^43^, ^44^, ^45^, ^46^, ^47^^,^^51^^,^^56^^,^^57^^,^^59^, ^60^, ^61^, ^62^^,^^66^^,^^70^^,^^71^^,^^77^^,^^80^^,^^81^ eight from Denmark,^35^^,^^39^^,^^48^^,^^69^^,^^74^^,^^76^^,^^79^^,^^82^ seven from Spain,^19^^,^^30^^,^^34^^,^^40^^,^^49^^,^^63^^,^^65^ five from the Netherlands,^50^^,^^52^, ^53^, ^54^^,^^64^ five from Sweden,^67^^,^^68^^,^^78^^,^^83^^,^^84^ four from Australia,^27^^,^^28^^,^^37^^,^^72^ two from China,^32^^,^^55^ one from the USA,^73^ one from Canada,^42^ one from New Zealand,^58^ one from Egypt,^75^ one from Italy,^36^ one from Malaysia^33^ and one from Nigeria.^29^ For further demographic information see Table 1.

**Table 1:** Demographics of the populations of the included studies.

Study (Country)	Study design and setting	Sample size and source of data (date)	Age (years)	Sex	CRC staging	Method used to identify the outcome	Outcome(s) to be predicted and number of events
Abdelhady 2021^75^ (Egypt)	Case-control Secondary care	CRC = 30 Pathological control = 30 Normal control = 30 Suez Canal University Hospital (June 2019–June 2020)	Mean (SD) CRC = 68 (7.3) Control pathological = 56.9 (6.3) Control normal = 59.5 (7.5)	CRC Male = 21 Female = 9 Pathological control Male = 12 Female = 18 Normal control Male = 15 Female = 15	I = 15 II = 10 III = 5 IV = 0	Pre-defined CRC cases, blood testing was utilised for the outcome	CRC = 30
Adelstein 2010^27^ (Australia)	Cross-sectional Secondary/Tertiary	Overall = 8204 Tertiary and non-tertiary practices/hospitals in NSW (April 2004–Dec 2006)	Median = 58 Range = 18-95	Male = 3860 Female = 4344	NR	Colonoscopy (if not visualised, additional tests of CT colonography or barium enema were performed to complete exam)	CRC = 159
Adelstein 2011^28^ (Australia)	Cross-sectional Secondary/Tertiary	Overall = 8204 Tertiary and non-tertiary practices/hospitals in NSW (April 2004–Dec 2006)	Median = 58 Range = 18-95	Male = 3860 Female = 4344	NR	Colonoscopy (if not visualised by follow up bowel investigations)	CRC = 159 Advanced Adenomas (≥25% villous features, high grade dysplasia, or >10 mm) = 468 Adenomas 6–9 mm = 286 Adenomas ≤5 mm = 507
Alatise 2018^29^ (Nigeria)	Cross-sectional Secondary	Overall = 362 Development = 217 Validation = 145 Three hospitals in southwest Nigeria (Training = OAUTHC; Validation = UCH and UITH) (Jan 2014–July 2016)	Median (range) Overall = 59.5 (44–95) Development = 60 (45–95) Validation = 5944–87	Development Male = 137 Female = 80 Validation Male = 99 Female = 46	Overall II = 19 III = 30 IV = 17	Colonoscopy	CRC Development = 38 Validation = 28
Ayling 2021^46^ (UK)	Retrospective cohort Secondary	Overall = 617 Barts Health NHS Trust (1st May 2020 included, after 6 months clinical outcomes were collected)	Median (range) = 58 (18–95)	Male = 314 Female = 303	NR	Clinical and radiological reports, endoscopy, and histological findings. Further investigation undertaken in 532 patients: Colonoscopy = 316 Abdominopelvic CT = 153 CT colonography = 54 Sigmoidoscopy alone = 6	CRC = 17 HRA = 28
Ballal 2010^61^ (UK)	Prospective cohort Secondary	Overall = 3457 Three consultant colorectal surgeons in a Welsh district general hospital. (Aug 2003–May 2008)	Mean (SD) Patients referred = 58.7 (16.2) Completed assessment = 59.1 (15.9)	Male = 1621 Female = 1836	NR	Either rigid or flexible sigmoidoscopy, colonoscopy, barium enema, or a combination of these.	CRC = 186
Blume 2016^76^ (Denmark)	Case-control Secondary	Overall = 4698 Final model for CRC = 300 Final model for AA = 302 Seven collaborating hospitals located in various Denmark locations. Three used for development and four for validation. (May 2010–Nov 2012)	Mean (SD) Overall = 63.5 (12.6) Development Control = 63.8 (7.04) CRC = 64.5 (7.01) Validation Control = 64.8 (5.76) CRC = 65.6 (6.09) Adenoma Development Control = 62.7 (7.33) AA = 63.1 (7.09) Validation Control = 62.5 (6.21) AA = 62.9 (5.9)	Overall Male = 2243 Female = 2455 Development (CRC) Male = 70 Female = 80 Validation (CRC) Male = 80 Female = 70 Development (AA) Male = 76 Female = 74 Validation (AA) Male = 76 Female = 76	Overall I = 101 II = 163 III = 139 IV = 108 NA = 1 Development I = 17 II = 30 III = 16 IV = 12 Validation I = 17 II = 21 III = 18 IV = 19	Colonoscopy Patients unable to undergo complete colonoscopy and patients with complete colonoscopy but without bowel pathology and persisting symptoms, were offered additional examination using combinations of x-ray with barium enema, ultrasound, computed axial tomography, and magnetic resonance imaging.	Development CRC = 75 AA = 75 Validation CRC = 75 AA = 76
Boulind 2022^62^ (UK)	Prospective cohort Secondary	Overall = 558 Model = unclear Three NHS trusts (Yeovil, North Bristol, and St James, Leeds); screened from consecutive fast track CRC referrals and approached when attending colonoscopy or review. (Aug 2018–Dec 2020)	mean (range): 64 (18–89)	Male = 311 Female = 247	NR	Colonoscopy or CT	CRC = 18 (5 suspected at CT) Polyp = 134
Cama 2021^30^ (UK)	Retrospective cohort Primary	3460 patients returned a FIT sample, 1046 underwent any investigation and 701 patients had full colonic evaluation–it is unclear who was used in the analysis Medical records (cross referenced with the trust cancer datanase); Herts Valley UK (June 2019–July 2020)	Mean (IQR): 66 (56–76)	Male = 43% Female = 57%	NR	Colonic investigation—undefined	NR
Collins 2012^47^ (UK)	Retrospective cohort Primary	QResearch database (internal validation) = 1,236,601 THIN (external validation) Male = 417,560 (with imputation = 1,059,765) Female = 1,075,775 THIN database (external validation; 1st Jan 2000–30th June 2008)	Mean (SD) QResearch database Development = 50.1 (15) Validation = 50.1 (14.9) THIN database Median (IQR) Male = 47 (38–60) Female = 49 (38–63)	THIN database Male = 1,059,765 Female = 1,075,775	NR	Identification via the THIN database records.	THIN database CRC = 3712
Croner 2017^48^ (Denmark)	Retrospective cohort Secondary	Overall = 4698 Development = 3099 Validation = 1336 Endoscopy II database samples, collected from seven hospitals across Denmark. (2010–2012)	Mean (SD) All = 63.5 (12.6) Development Control = 62.7 (12.6) CRC = 69.7 (10.6) Validation Control = 62.9 (12.7) CRC = 70.1 (10.7)	Overall Male = 2243 Female = 2455 Development Control Male = 1286 Female = 1473 CRC Male = 196 Female = 144 Validation Control Male = 539 Female = 650 CRC Male = 92 Female = 55	Overall I = 101 II = 163 III = 139 IV = 108 Development I = 74 II = 105 III = 87 IV = 73 Validation I = 25 II = 50 III = 45 IV = 27	Colonoscopy	CRC Development = 340 Validation = 147
Cubiella 2016^19^ (Spain)	Cross-sectional Primary and secondary	Development = 1572 Validation = 1481 Development cohort consisted of consecutive patients with gastrointestinal symptoms referred for colonoscopy from primary and secondary health care to Complexo Hospitalario Universitario de Ourense, Spain (March 2012–Sept 2013). Validation cohort included a prospective cohort of patients with gastrointestinal symptoms referred for colonoscopy in 11 hospitals in Spain (March 2014–March 2015).	Median (range) Development = 68 (20–96) Validation = 64 (19–101)	Development Male = 810 Female = 762 Validation Male = 719 Female = 762	Development 0 = 2.8% I = 18.6% II = 25.1% III = 37.7% IV = 15.8% Validation NR	Colonoscopy	CRC Development = 214 Validation = 136 AN Development = 251 Validation = 197
Cubiella 2017^18^ (Spain and Scotland)	Retrospective cohort Primary and secondary	Development = 1572 Validation = 3976 Development Patients referred to colonoscopy in Ourense, Spain (March 2012–Sept 2013) Validation Five studies evaluating diagnostic accuracy of different FIT analytical systems for CRC, AN, and SCL. Three Scottish and Two Spanish (dates not reported)	Median (range) Overall = 65 (15–100) Development = 68 (25–96) Validation (five studies) 1 = 60 (15–89) 2 = 64 (16–90) 3 = 63 (18–84) 4 = 63 (18–90) 5 = 64 (19–100)	Overall (%) Male = 46.2 Development Male = 51.5 Validation 1 = 40.4 2 = 45.5 3 = 42.1 4 = 46.9 5 = 48.7	NR	Colonoscopy	CRC (%) Development = 13.7 Validation 1 = 2.1% 2 = 3.7% 3 = 2.3% 4 = 3% 5 = 9%
Digby 2019^85^ (Scotland)	Cross-sectional Primary and secondary	Overall = 1447 Patients presenting to primary care with symptoms, who underwent FIT and colonoscopy at NHS Tayside (Dec 2015–Dec 2016)	NR	NR	NR	Colonoscopy In addition, linkage with the Scottish Cancer Registry was performed to ensure that all cases of CRC had been identified.	CRC = 94
Ellis 2005^31^ (UK)	Cross-sectional Primary	Overall = 319 Analysis = 266 Three practices, one in a market/rural community, one in a suburban area, and one in an inner-city. GP asked to identify patients whose complaint was rectal bleeding and other symptoms, with rectal bleeding. (Study dates NR)	Mean (range) Male = 56 (35–84) Female = 62 (35–94)	NR	NR	Flexible sigmoidoscopy = 219 Barium enema = 37 Colonoscopy = 24	CRC = 11
Ewing 2016^83^ (Sweden)	Case-control Primary	Overall = 2681 Cases = 542 Control = 2139 Swedish Cancer register, a database in Region Vastra Gotaland (RVG)	Median (range) Cases: 72 (30–94) Controls: 72 (30–94)	NR	I = 118 II = 223 III = 201	Swedish Cancer register	CRC = 542
Fernandez-Banares 2019^63^ (Spain)	Prospective cohort Primary and secondary	Overall = 1495 Development = 867 Validation = 628 Three hospitals in Spain. (March 2014–Sept 2016)	NR	Development ACN Male = 103 Female = 68 Control Male = 311 Female = 385 Validation ACN Male = 89 Female = 59 Control Male = 224 Female = 256	NR	Colonoscopy	ACN (CRC + AA) Development CRC = 67 AA = 104 Validation CRC = 49 AA = 99
Fijten 1995^64^ (Netherlands)	Prospective cohort Primary	Overall = 269 83 GPs in Limburg, Netherlands. (Sept 1988–April 1990)	Mean (SD) = 42 (15)	Male = 118 Female = 151	NR	At the end of the initial consultation 8% of patients were referred to a medical specialist (5% to an internist, 3% to a surgeon). Endoscopy or roentgenography was requested for 14% and 10% of patients, respectively. Follow up after at least one year, a total of 24% of patients had been referred, 14% internist, 5% surgeon, 2% to another specialist and 3% to several specialist. 31% had further investigations initiated by the GP by: sigmoidoscopy (9%) colon roentgenography (9%) proctoscopy (8%) sonography (6%) colonoscopy (2%) some patients had more than 1 investigation	CRC = 9 Polyps = 6
Hamilton 2005^77^ (UK)	Case-control Primary	Overall = 2093 Cases = 349 Control = 1744 Registry that collects registrations from three main sources: direct notifications by clinicians, routine notification of all positive histology results and forwarding of patient lists from oncology treatment centre (Devon and Exeter). (1998–2002)	NR	Cases Male = 177 Female = 172 Control Male = 885 Female = 889	NR	Cancer registry at the Royal Devon and Exeter hospital. Supplemented by computerised searches at every practice identified for any missing from the cancer registry.	CRC = 349
Herrero 2018 (Spain)	Retrospective cohort Primary and secondary	Overall = 1572 Uses COLONPREDICT cohort, see Cubiella 2016.	NR	NR	NR	Colonoscopy	CRC = 214
Hijos-Mallada 2023^65^ (Spain)	Prospective cohort Primary and secondary	571	Median (IQR) Significant pathology = 70 (59.5–80.5) Non-significant findings = 60 (48.5–71.5)	Significant pathology Male = 67 Female = 51 Non-significant findings Male = 205 Female = 248	NR	Colonoscopy	CRC = 30 Adenoma = 53
Hippisley-Cox 2012^66^ (UK)	Prospective cohort Primary	Overall = 3,880,944 Development = 2,351,052 Validation = 1,236,601 QResearch database (v.30). All practices in England and Wales that had been using their EMIS (Egton Medical Information System) computer system for at least a year were included. Two thirds of practices were randomly allocated to the development cohort and the remaining third to the validation.	mean (SD) development = 50.1 (15) validation = 50.1 (14.9)	Development Male = 1,178,382 Female = 1,172,670 Validation Male = 620,240 Female = 616,361	NR	Database: incident of CRC during the 2 years after study entry. Either on GP record or on their linked ONS cause of death record.	CRC Development = 4798 Validation = 2603
Hogberg 2020^68^ (Sweden)	Prospective cohort Primary	Overall = 18,913 Analysis = 15,789 (Those with three samples of FIT; Note: number varies depending on equipment and combination)	Median (IQR) = 65 (48–75)	Male = 7489 Female = 11,424	NR	Incident of CRC during 2 years after FIT completion. Information about patients diagnosed with CRC within 2 years of the FITs was obtained from the Swedish Cancer Register. Note: FIT was measured using 4 different analysers (Actim Fecal Blood, Analyz FOB, Chemtrue FOB, Diaquick FOB) and the results are reported split by each analyser	CRC = 304 (Note: number varies depending on equipment and combination)
Hogberg 2017^67^ (Sweden)	Prospective cohort Primary and secondary	Overall = 391 Analysis = 364 Four health care centres in the region Jamtland Harkedaken. (30 Jan 2013–31 May 2014)	Median = 63	Male = 138 Female = 253	NR	Colonoscopy In the results they do mention that some patients underwent CT (abdominal and colon). Some had barium enema. All patients that agreed to participate were followed for 2 years, and data on bowel imaging and clinical outcome were collected from their medical records	CRC = 8 HRA = 8
Hoogendoorn 2016^50^ (Netherlands)	Retrospective cohort Primary	Overall >90,000 Final model number is unclear Anonymised primary care dataset originating from a network of GPs centred around the Utrecht University Medical Center. (1st July 2006–31st Dec 2011)	NR	NR	NR	Electronic medical records	CRC = 588
Jin 2012^32^ (China)	Cross-sectional Secondary	Overall = 201 Beijing military general hospital. (Oct 2009–March 2010)	Mean (range) = 67 (31–91)	Male = 153 Female = 48	NR	Colonoscopy	CRC = 21 AA = 47
Johansen 2015^69^ (Denmark)	Prospective cohort Secondary	Overall = 4496 Six Danish hospitals. (Jan 2004–Dec 2005)	Median (range) = 61 (18–97)	Male = 2064 Female = 2432	NR	Colonoscopy = 2738 Flexible sigmoidoscopy = 1701 Rigid proctoscopy = 52 Unknown = 5	Colon cancer = 184 Rectal cancer = 109 adenomas = 854
Johnstone 2002^51^ (UK)	Retrospective cohort Primary	Overall = 4968 NHS Greater Glasgow and Clyde. (Aug 2018–Jan 2019)	Median (range) = 59 (16–97)	Male = 2102 Female = 2866	NR	Cancer registry used to identify CRCs Colonoscopy = 1330 CT/CT colon = 153	CRC = 61
Koning 2015^52^ (Netherlands)	Retrospective cohort Primary	Overall = 2787 Julius General Pracitioners Network (JPGN) database. (Utretcht Netherlands; 1st Jan 2007–31st Dec 2011)	Mean (SD) = 58 (13.9)	Male = 1260 Female = 1527	NR	Outcomes were extracted from colonoscopy test results, relevant specialist letters or, if these were not readily available or specifically coded, outcome was based on the presence of corresponding ICPC codes within 1 year after referral for colonoscopy.	CRC = 57 HRA = 31
Kop 2015^53^ (Netherlands)	Retrospective cohort Primary	Overall = 127,304 Numbers in analysis are unclear. Two GP databases in Utreccht Netherlands. (1st July 2006–31st Dec 2011)	NR	NR	NR	Electronic medical records	CRC = 651
Kop 2016^54^ (Netherlands)	Retrospective cohort Primary	Overall = 263,879 Three GP databases in urban regions of the Netherlands. (2007–2011)	NR	NR	NR	Electronic medical records	CRC = 1292
Law 2014^33^ (Malaysia)	Cross-sectional Primary and secondary	Overall = 1013 A large teaching institution serving multi-ethnic Asian urban population (Chinese, Malays, and Indians; July 2009–March 2011).	Mean (SD) = 59.9 (13.7) Range = 18-95	Male = 483 Female = 530	NR	Colonoscopy	CRC = 114 Adenomas = 172
Liu 2021^55^ (China)	Retrospective cohort Secondary	Overall = 1142 Development = 686^a^ Validation = 228^a^ Testing = 228^a^ Samples from human aerospace hospital and peoples hospital of Ningxiang. (Study dates not reported)	Mean (range) = 49.2 (26–83)	Male = 577 Female = 565	I-II = 67 III-IV = 113	Colonoscopy	CRC = 180 Adenoma = 60 Polyp = 273
Lucoq 2022^81^ (UK)	Unclear (abstract only)	A single health board (undefined) 2018–2021	Median = 65 (NR)	Ratio M:F = 0.9:1.0	NR	Colonoscopy	unclear
Lue 2020^86^ (Spain)	Cross-sectional Primary and secondary	Overall = 404 Referred to HCU Lozano Blesa. (June 2015–April 2017)	Median (IQR) = 59 (47–69)	Male = 166 Female = 238	NR	Colonoscopy	CRC = 16 AA = 39
Mahadavan 2012^70^ (UK)	Prospective cohort Secondary	Overall = 714 Patients obtained from a population of around 400,000, with approximately 125–140 (May 2008–May 2009)	Median (IQR) CRC = 74 (70–80) Control = 70 (62–80)	Male = 319 Female = 395	NR	Colonoscopy or CT (generally within 2–3 weeks)	CRC = 72
Malagon 2019^34^ (Spain)	Cross-sectional Primary and secondary	Overall = 333 Patients referred to Complexo Hospitalario de Ourense. (Study dates not reported)	Mean (range) CRC = 73 (53–91) AA = 65 (44–83) non-AA = 67 (37–89) normal = 61 (20–87)	Female n (%) CRC = 17 (10) AA = 15 (8.8) non-AA = 32 (18.8) normal = 106 (62.4)	0 = 3 I = 6 III = 21 IV = 8	Colonoscopy	CRC = 48 AA = 30
Marshall 2011^56^ (UK)	Retrospective cohort Primary	Overall = 43,791 THIN Database. (Jan 2001–July 2006)	Mean (range) = 70.6 (30–105)	Male = 23,253 Female = 20,538	NR	Identification via the THIN database records.	CRC = 5477
Mowat 2016^71^ (UK)	Prospective cohort Primary	Overall = 2173 Analysis = 755 At the point of referring patients to the colorectal pathway GPs were prompted to request FHb and FC tests alongside full blood count, urea and electrolytes and C reactive protein and record the presenting symptoms via NHS Tayside electronic test software. If they had more than one symptom, they were attributed one in order of decreasing clinical importance: rectal bleeding, anaemia, diarrhoea, altered bowel habit, abdominal pain, and weight loss. (Oct 2013–March 2014)	Median (IQR) = 64 (52–73) Range = 16–90	Analysed: Male = 342 Female = 413	NR	Colonoscopy	CRC = 28 HRA = 41
Nemlander 2023a^78^ (Sweden)	Case-control Primary	Overall = 2681 Development = 2013 Validation = 668 Swedish cancer register and the VEGA regional administrative healthcare database. Dates NR	Age at diagnosis date Mean (SD) Cases = 71.2 (11.7) Controls = 71.2 (11.7)	Male Cases = 272/542 Controls = 1074/2139	I = 118 II = 278 III = 130	Registry	Non-metastatic CRC Development = 407 Validation = 135
Nemlander 2023b^84^ (Sweden)	Case-control Primary	Overall = 14,548 Stockholm regional health care administration database (VAL) 2015–2019	Age at diagnosis date Mean (SD) Cases = 70.7 (12.6) Controls = 70.6 (12.5)	Male Cases = 1483/2920 Controls = 5901/11,628	I = 731 II = 846 III = 1343	Registry	Non-metastatic CRC cases = 2920
Norrelund 1996^35^ (Denmark)	Cross-sectional Primary	Study 1 = 208 Study 2 = 209 (analysis = 156) Study 1 Every fourth GP registered in the directory of the Danish medical associaton (n = 750) were to participate in the study. The GPs were to include a maximum of three consecutive patients, 40 years and older, who presented with a first episode of overt rectal bleeding within the previous six months. (1989–1991) Study 2 Using the same method as in study 1 but omitting the 750 GPs who were previously invited, 450 GPs were invited to participate in a second study. Each GP was to contribute a maximum of four patients. (1991–1992)	NR	Study 1 Male = 97 Female = 111 Study 2 NR for all those in study 2	NR	A yearly letter to GP or microscopically verified	Study 1 CRC = 32 Polyps = 16 Study 2 CRC = 25
Parente 2012^36^ (Italy)	Cross-sectional Secondary	Overall = 280 Analysis = 278 (two patients excluded without reason) Three participating centres (A. Manzoni Hospital, Lecco, S. Orsola Hospital, Bologna, and Regina Margherita Hospital, Rome; over a 6 month period of an unspecified study period)	Mean (range) = 67 (50–80)	Male = 157 Female = 123	NR	Colonoscopy	CRC = 47 AA = 85 Low risk adenomas = 22
Payne 1983^37^ (Australia)	Cross-sectional Secondary	Overall = 159 Recruitment setting and dates not specified.	NR	NR	NR	Sigmoidoscopy, air contrast barium enema and/or colonoscopy	CRC = 46
Rai 2008^38^ (UK)	Cross-sectional Secondary	Overall = 1422 Three hospitals of the University Hospitals of Leicester National Health Service (NHS) Trust and the six peripheral community hospitals in Leicestershire. (Sept 2003–Aug 2004)	Median (range) = 68 (21–95)	Male = 751 Female = 671	NR	All referrals were followed up during the course of hospital investigations until a final diagnosis, benign or malignant, was made. Exact method not specified.	CRC = 83
Rasmussen 2017^82^ (Denmark)	Cross-sectional Secondary	Overall = 4773 Final analysis = 4105 Endoscopy II project, collected from 7 hospitals across of Denmark (Aarhus, Bispebjerg, Herning, Hillerød, Horsens, Hvidovre and Randers). (May 2010–Nov 2012)	Median (range) = 64 (18–95)	Male = 1964 Female = 2141	I-II = 225 III-IV = 216	Colonoscopy	CRC = 441 HRA = 342
Rasmussen 2021^79^ (Denmark)	Case-control Secondary	Overall = 4698 Final analysis = 784 Endoscopy II project, collected from 7 hospitals across of Denmark (Aarhus, Bispebjerg, Herning, Hillerød, Horsens, Hvidovre and Randers). (May 2010–Nov 2012)	Median (range) CRC = 70 (38–92) HRA = 66 (42–96) Clean colorectum = 60 (28–87)	CRC Male = 127 Female = 69 HRA Male = 54 Female = 44 Clean colorectum Male = 94 Female = 102	I = 49 II = 49 III = 49 IV = 49	Colonoscopy	CRC = 196 HRA = 96
Rodriguez-Alonso 2015^40^ (Spain)	Cross-sectional Secondary and tertiary	Overall = 1003 The Endoscopy Department of Bellvitge University Hospital. Referrals originated from general practitioners and community gastroenterologists, as well as from the hospital environment. (Sept 2011–Oct 2012)	NR	Male = 470 Female = 533	NR	Colonoscopy	CRC = 30 AN = 133
Selvachandran 2002^41^ (UK)	Cross-sectional Secondary	Overall = 2268 Recruitment setting not specified. (Oct 1999–Oct 2001)	NR	Male = 1037 Female = 1231	Dukes A = 22 Other stages not reported	Endoscopy (specific procedure is not reported)	CRC = 95
Simpkins 2017^42^ (Canada)	Cross-sectional Secondary	Overall = 1981 Consecutive, unselected patients newly referred from primary care to two secondary care centres. The McMaster University Medical Center and St. Joseph's Healthcare. (Jan 2008–Dec 2012)	Mean = 49.3	Male = 730 Female = 1251	NR	Colonoscopy	CRC = 47
Stapley 2017^80^ (UK)	Case-control Primary	Overall = 5640 Data collected prospectively from the Clinical Practice Research Datalink (CPRD). The CPRD maintains records from nearly 700 participating practices in the UK. (Jan 2000–Dec 2013)	Range = 18–49	Cases Males = 855 Females = 806 Controls Males = 1828 Females = 2151	NR	Clinical Practice Research Datalink (CPRD) using diagnostic medical codes.	CRC = 1661
Steffen 2014^72^ (Australia)	Prospective cohort Primary and secondary	Development (45 and up) = 197,874 Validation (MCCS) = 24,233 Retrospective analysis of two prospective studies, the 45 and up study (development) and the Melbourne collaborative cohort study (validation).	Mean (SD) at baseline Development = 61.2 (16.3) Validation = 65.7 (8.7)	Development^a^ Male = 84,492 Female = 113,382 Validation^a^ Male = 9354 Female = 14,879	NR	Cancer registry	Development CRC = 1103 Validation CRC = 224
Thompson 2017^57^ (UK)	Retrospective cohort Secondary	Overall = 26,972 Development = 17,403 Validation = 11,602 All patients referred by their GP to the colorectal surgical outpatient clinics at St Mary's Hospital, Queen Alexandra Hospital and two peripheral hospitals in and near Portsmouth. (1986–2007)	Mean (SD) Development = 60.1 (16.3) Validation = 60.1 (16.5)	Development Male = 7651 Female = 9752 Validation Male = 5043 Female = 6559	NR	Sigmoidoscopy and/or whole colonic imaging Cancers not diagnosed after the first visit were included if detected within 3 years, mainly by referral back to hospital and local hospital audit. A small number were detected by comparison of the database with the Regional Cancer Registry.	CRC = 1626
Turvill (2018)^43^ (UK)	Cross-sectional Secondary	Overall = 515 A single centre in the UK. (Feb 2016–March 2017)	Median (IQR) = 69 (61–76)	Reported that both sexes were equally represented	NR	Patients undergoing full colonoscopy or CT colonography or a lesser investigation (such as CT abdomen/pelvis with contrast plus flexible sigmoidoscopy) limited by the identification of pathology were included in the data analysis.	CRC = 27
Wells 2014^73^ (USA)	Prospective cohort Primary	Male = 80,062 Female = 100,568 Prospective cohort, followed up for 11.5 years, or until development of CRC, or until 31st Dec 2004. (Cohort study started between 1993 and 1996).	Mean (SD) Male CRC = 64.2 (7.8) No CRC = 59.8 (8.9) Female CRC = 64 (7.9) No CRC = 59.5 (8.8)	Male = 80,062 Female = 100,568	NR	Registry data (information regarding IBD disease, sigmoidoscopy or colonoscopy not known)	CRC Male = 1486 Female = 1276
Whitfield 2018^58^ (New Zealand)	Retrospective cohort Secondary	Development = 2236 Validation = 958 Single centre in New Zealand: Palmerston North Hospital. (July 2005–June 2016)	NR	NR	NR	Colonoscopy	CRC Development = 170 Validation = 75
Widlak 2017^44^ (UK)	Cross-sectional Secondary	Overall = 430 Single centre in the UK: University Hospitals Coventry and Warwickshire UHCW National Health Service (NHS) Trust. (Jan 2015–March 2016)	Median (IQR) = 67 (57–76) Range = 29–93	Male = 210 Female = 220	NR	Colonic investigations –Colonoscopy or CT colonography or CT abdomen/pelvis with contrast plus flexible sigmoidoscopy.	CRC = 24 (plus 1 high grade dysplasia) Adenoma (with low grade dysplasia and other pathology) = 28 Adenoma (with low grade dysplasia) = 42
Widlak 2018^45^ (UK)	Cross-sectional Tertiary	Overall = 562 Single tertiary care centre in UK. (Study dates not reported)	Median (range) = 68 (29–89)	Male = 286 Female =	NR	Endoscopic or radiological colonic cross-sectional imaging.	CRC = 35 HRA = 27 All adenomas = 94
Wilhelmson 2017^39^ (Denmark)	Prospective cohort Secondary	Overall = 4692 Final analysis = 4521 7 Collaborating hospitals in Denmark. (May 2010–Nov 2012)	NR	NR	I = 101 II = 163 III = 139 IV = 108 1 not available	Colonoscopy	CRC = 400 HRA = 399
Wilhelmsen 2018^74^ (Denmark)	Prospective cohort Secondary	Overall = 3732 Final analysis = 3555 7 Collaborating hospitals in Denmark. (May 2010–Nov 2012)	NR	NR	I = 82 II = 127 III = 109 IV = 84	Colonoscopy Those without colonoscopy were offered additional examination, ie, gastroscopy, X-ray with barium enema, ultrasonography, computer-assisted tomography, and/or magnetic resonance imaging. (These tests likely for evaluation of extracolonic cancers).	CRC = 400 Adenomas = 502
Wilson 2012^59^ (UK)	Retrospective cohort Primary	Overall = 748 Stage I = 632 Stage II = 249 19 General Practices in the South Birmingham area. Patients recruited through mailed questionnaires. (Study dates not reported)	Median (IQR) = 59 (54–63) Range = 50–70	Male = 356 Female = 392	NR	Colonoscopy	CRC = 46 (8 sample were lost)
Withrow 2022^60^ (UK)	Retrospective cohort Primary and secondary	Overall = 18,656 Final analysis = 16,604 Data from the Oxford University Hospital (OUH), 67 GPs in Oxford. (March 2017–Dec 2020; 6 month follow up allowed up until June 2021)	Median = 61	Male = 7019 Female = 9585	NR	The composite reference standard incorporated the review of multiple-linked databases (hospital clinical records, pathology results, and endoscopy and radiology reports) for evidence of a new colorectal cancer diagnosis	CRC = 139

a Estimated from provided percentage. UK = United Kingdom; USA = United States of America; NR = not reported; CRC = colorectal cancer; AN = advanced neoplasia; AA = advanced adenoma; HRA = high risk adenoma; SD = standard deviation; IQR = inter-quartile range; CT = computed tomography.

### Models including FIT

Twenty-three of the studies included FIT (n = 22) or gFOBT (n = 1) combined with one or more other variables (Table 2).^18^^,^^19^^,^^30^^,^^32^^,^^34^^,^^36^^,^^40^^,^^43^, ^44^, ^45^, ^46^^,^^49^^,^^51^^,^^60^^,^^63^^,^^65^^,^^67^^,^^68^^,^^70^^,^^71^^,^^81^ Of these, ten studies reported model development only,^30^^,^^34^^,^^40^^,^^43^, ^44^, ^45^^,^^60^^,^^65^^,^^70^^,^^81^ four studies presented validations of models,^30^^,^^46^^,^^49^ three studies presented both development and validation,^18^^,^^19^^,^^63^ and six were classed as PPV only studies (i.e. they reported PPVs for FIT in combination with at least one other factor).^32^^,^^36^^,^^51^^,^^67^^,^^68^^,^^71^

**Table 2:** Results from studies including faecal blood tests (FIT/gFOBT) combined with one or more other variables.

Study (type of study)	Predictors (final model)	Modelling method	AUC (95% CI)	Sensitivity % (95% CI)	Specificity % (95% CI)	PPV % (95% CI)	NPV % (95% CI)
Ayling 2021^46^ (validation; ColonFlag and FAST score)	ColonFlag (band 3) Age Sex Full blood count FAST score (>4.5) Age Sex FIT (≥4 μg Hb/g)^b^	NR directly ColonFlag = machine learning FAST score = Logistic regression	NR	CRC FAST: 72.7 (39–94) ColonFlag: 81.8 (48.2–97.7) CRC + HRA FAST: 60 (42.1–76.1) ColonFlag: 42.9 (26.3–60.7) FIT + ColonFlag CRC: 100 (71.5–100) CRC + HRA: 85.7 (69.7–95.2)	CRC FAST: 80.6 (76.2–84.5) ColonFlag band 3: 73.5 (68.7–77.9) CRC + HRA FAST >4.5: 83 (78.7–86.8) Colonflag band 3: 73.4 (68.4–77.9) FIT + colonflag CRC: 49.6 (44.4–54.8) CRC + HRA: 51.6 (46.2–56.9)	CRC alone FAST >4.5: 9.9 (6.7–14.3) Colonflag band 3: 8.3 (6.1–11.1) CRC + HRA FAST >4.5: 25.9 (19.7–33.3) Colonflag band 3: 13.7 (9.5–19.5) FIT + colonflag CRC: 5.5 (4.9–6) CRC + HRA: 14.9 (12.9–17.3)	CRC alone FAST >4.5: 99 (97.5–99.6) Colonflag band 3: 99.3 (97.6–99.8) CRC + HRA FAST >4.5: 95.4 (93.3–96.9) Colonflag band 3: 92.8 (90.6–94.6) FIT + colonflag CRC: 100 CRC + HRA: 97.3 (94.1–98.8)
Cama 2021^30^ (validation; FAST score)	FAST score (>2.12) Age Sex FIT (>10 μg/g) NG12 criteria (comparison)	NR Compared FAST score and NG12 criteria using MedCalc software	NR	FAST >2.12 = 1.00 (0.93–1.00) NG12 = 0.82 (0.67–0.91)	FAST >2.12 = 0.25 (0.24–0.27) NG12 = 0.42 (0.4–0.43)	NR	NR
Cubiella 2016^19^ (development and validation; COLONPREDICT)	Age Sex Change in bowel habit Rectal bleeding Benign anorectal lesion Rectal mass Anaemia CEA Previous colonoscopy (10 yrs) Aspirin use FIT (≥20μ Hb/g)	Logistic regression	CRC Development = 0.92 (0.91–0.94) Validation = 0.92 (0.9–0.94) ACN Development = 0.83 (0.8–0.85) Validation = 0.82 (0.79–0.85)	Development 5.6+ CRC = 90.1 (85.1–93.6) ACN = 66.7 (61.8–71.2) 3.5+ CRC = 99.5 (97–100) ACN = 89.5 (86.1–92.2) Validation 5.6+ CRC = 87.1 (79.9–92.1) ACN = 66 (60.3–71.3) 3.5+ CRC = 100 (96–100) ACN = 88.2 (83.9–91.5)	Development 5.6+ CRC = 78.7 (76.4–80.9) ACN = 82.3 (79.9–84.4) 3.5+ CRC = 45.8 (43.1–48.2) ACN = 50.1 (47.2–53.1) Validation 5.6+ CRC = 79.3 (76.9–81.4) ACN = 83.5 (81.2–85.7) 3.5+ CRC = 46.8 (44–49.6) ACN = 50.7 (47.7–53.7)	Development 5.6+ CRC = 40.7 (36.2–45.3) 3.5+ CRC = 22.9 (20.3–25.8) Validation NR	Development 5.6+ CRC = 98 (96.9–98.7) 3.5+ CRC = 99.8 (98.9–100) Validation NR
Cubiella 2017^18^ (development and validation; FAST Score)	Age Sex FIT (in equation 0, 20, or 200 μg Hb/g)^b^ FAST scores assessed ≥4.50 and ≥ 2.12	Logistic regression	CRC Development = 0.88 (0.85–0.9) Validation = 0.91 (0.9–0.93) ACN Development = 0.82 (0.8–0.84) Validation = 0.79 (0.76–0.8)	Development CRC: 4.50+ = 89.8 (84.7–93.3) 2.12+ = 100 (97.8–100) ACN: 4.50+ = 75.4 (70.9–79.4) 2.12+ = 98.8 (97.1–99.6) Validation CRC: 4.50+ = 89.3 (84.1–93) 2.12+ = 100 (97.7–100) ACN: 4.50+ = 60.7 (56.6–64.7) 2.12+ = 96.7 (94.9–98)	Development CRC: 4.50+ = 71.3 (68.8–73.7) 2.12+ = 13.9 (12.1–15.9) ACN: 4.50+ = 76.9 (74.3–79.3) 2.12+ = 15.9 (13.9–18.2) Validation CRC: 4.50+ = 82.3 (81.1 = 83.5) 2.12+ = 19.8 (18.6–21.1) ACN: 4.50+ = 85.4 (84.1–86.5) 2.12+ = 21.5 (20.1–22.9)	Development CRC: 4.50+ = 33.2 (29.4–37.2) 2.12+ = 15.6 (13.7–17.6) ACN: 4.50+ = 54.4 (50.2–58.5) 2.12+ = 30 (27.6–32.5) Validation CRC: 4.5+ = 21.7 (NR) ACN: 4.5+ = 41.7 (NR)	Development CRC: 4.50+ = 97.8 (96.6–98.6) 2.12+ = 100 (97.5–100) ACN: 4.50+ = 89.6 (87.4–91.4) 2.12+ = 97.3 (93.5–99) Validation NR
Digby 2019^85^ (validation; FAST Score)	Age Sex FIT (in equation 0, 20, or 200 μg Hb/g)^b^ FAST score ≥2.12	Logistic regression	NR	2.12+ = 99 (94.3–100)	2.12+ = 22.4 (20.2–24.7)	2.12+ = 8.2 (8–8.5)	2.12+ = 98.9 (97.7–100)
Fernandez Banares 2019^63^^,^^d^ (development and validation; COLONOFIT)	Age MAXFIT (maximum f-Hb value of three samples) NSAMPLES >4 (number of samples >4 μg Hb/g faeces) Previous colonoscopy (5 yrs) Smoking status	Bayesian logistic regression (Bootstrapping completed for internal validation; development)	Development CRC = 0.93 (0.91–0.95) CRC + AA = 0.865 (0.83–0.89) Validation CRC = 0.86 (0.025^b^) CRC + AA = 0.79 (0.02^b^)	Validation CRC = 96 (85–99) CRC + AA = 79 (72–85.4) Development + Validation CRC = 98 (93–99.7) CRC + AA = 85 (80.3–88)	Validation CRC = 52 (48–56) CRC + AA = 58 (54.2–63) Development + Validation CRC = 53 (51–56) CRC + AA = 60 (57.4–63)	Validation CRC = 14.4 (11–19) CRC + AA = 37 (32–42.7) Development + Validation CRC = 15 (13–18) CRC + AA = 36 (33.2–40)	Validation CRC = 99.3 97–99.9) CRC + AA = 90 (87–93.2) Development + Validation CRC = 99.7 (99–100) CRC + AA = 93.5 (91.5–95)
Herrero 2018^49^ (validation; COLONPREDICT, FAST Score, 2017 NG12 and CG27 NICE)	Various combinations for referral, only NG12 was directly reported: Age Weight loss Abdominal pain Iron deficiency anaemia Change in bowel habit Rectal mass Abdominal mass FIT	NR	NG12 = 0.53 (0.49–0.57) CG27 = 0.59 (0.55–0.63) COLONPREDICT = 0.92 (0.91–0.94) FAST Score (≥4.50) = 0.87 (0.85–0.89)	NG12 = 100 (97.8–100) CG27 = 68.2 (61.5–74.3) NB: for COLONPREDICT and FAST score, see Cubiella 2016; 2017	NG12 = 6.8 (5.6–8.4) CG27 = 50.3 (47.6–53) NB: for COLONPREDICT and FAST score, see Cubiella 2016; 2017	NG12 = 14.5 (12.8–16.5) CG27 = 17.8 (15.3–20.6) NB: for COLONPREDICT and FAST score, see Cubiella 2016; 2017	NG12 = 100 (95–100) CG27 = 91 (89–93) NB: for COLONPREDICT and FAST score, see Cubiella 2016; 2017
Hijos-Mallada 2023^65^ (development)	FIT (qualitative) Transferrin (>0.4 μg/g) Lactoferrin (>10 μg/g) FC (>50 μg/g)	Logistic regression	CRC = 0.872 (0.815–0.929) Adenoma = 0.673 (0.599–0.747)	CRC = 50 (NR) Adenoma = 57 (NR)	CRC = 96.5 (NR) Adenoma = 94 (NR)	CRC = 44.1 (NR) Adenoma = 8.8 (NR)	CRC = 97.2 (NR) Adenoma = 90.7 (NR)
Hogberg 2017^67^ (PPV)	FIT (one or more samples were positive, i.e. ≥25 μg Hb/g) Faecal Calprotectin (≥100 μg/g) Anaemia Iron deficiency	NA	NA	FIT positive and/or FC 100ug/g+ = 87.5 FIT positive and/or FC 20ug/g+ = 100 FIT positive and/or anaemia = 100 FIT positive and/or iron deficiency = 100 FIT positive and/or anaemia and/iron deficiency = 100	FIT positive and/or FC 100ug/g+ = 61.1 FIT positive and/or FC 20ug/g+ = 40.3 FIT positive and/or anaemia = 60 FIT positive and/or iron deficiency = 59.2 FIT positive and/or anaemia and/iron deficiency = 54.8	FIT positive and/or FC 100ug/g+ = 4.7 FIT positive and/or FC 20ug/g+ = 3.5 FIT positive and/or anaemia = 5.2 FIT positive and/or iron deficiency = 5.1 FIT positive and/or anaemia and/iron deficiency = 4.7	FIT positive and/or FC 100ug/g+ = 99.6 FIT positive and/or FC 20ug/g+ = 100 FIT positive and/or anaemia = 100 FIT positive and/or iron deficiency = 100 FIT positive and/or anaemia and/iron deficiency = 100
Hogberg 2020^68^ (PPV)	FIT (≥2–50 μg Hb/g depending on machine brand) Anaemia Thrombocytosis	NA	NA	FIT positive + Anaemia Actim Fecal Blood = 52 Analyz FOB = 38.3 Chemtrue FOB = 55.2 Diaquick FOB = 30.6 FIT positive + Thrombocytosis Actim Fecal Blood = 14.3 Analyz FOB = 17.3 Chemtrue FOB = 20.7 Diaquick FOB = 12.1	FIT positive + Anaemia Actim Fecal Blood = 88 Analyz FOB = 90.8 Chemtrue FOB = 89.2 Diaquick FOB = 91.8 FIT positive + Thrombocytosis Actim Fecal Blood = 96.2 Analyz FOB = 96.8 Chemtrue FOB = 95.6 Diaquick FOB = 98.1	FIT positive + Anaemia Actim Fecal Blood = 7.9 (5.5–10.3) Analyz FOB = 8.6 (6.4–10.7) Chemtrue FOB = 8.9 (4.7–13) Diaquick FOB = 8.3 (4.2–14.3) FIT positive + Thrombocytosis Actim Fecal Blood = 7.6 (1.8–13.4) Analyz FOB = 10.7 (6.6–14.9) Chemtrue FOB = 8.7 (2–15.3) Diaquick FOB = 13.8 (3.9–31.7)	FIT positive + Anaemia Actim Fecal Blood = 98.9 (98.6–100) Analyz FOB = 98.5 (98.2–98.8) Chemtrue FOB = 99.1 (98.5–99.6) Diaquick FOB = 98.2 (97.4–98.8) FIT positive + Thrombocytosis Actim Fecal Blood = 98 (97.4–98.6) Analyz FOB = 98.1 (97.8–98.5) Chemtrue FOB = 98.3 (97.7–99) Diaquick FOB = 97.8 (96.8–98.5)
Johnstone 2022^51^ (PPV)	FIT (categorised: <10 μg/g, 10–149 μg/g, 150–399 μg/g, and ≥400 μg/g) Anaemia	NA	NA	98.2 (NR)	65.4 (NR)	3.99 (NR)	99.96 (NR)
Jin 2012^32^ (PPV)	FIT (≥0.2 μg/ml) Faecal transferrin test	NA	NA	CRC = 47.6 AA 10 mm+ = 30.6 AA <10 mm = 36.4 AA + CRC = 36.8	CRC = 78.3 AA 10 mm+ = NR AA <10 mm = NR AA + CRC = 78.2	CRC = 20.4 AA 10 mm+ = 22.4 AA <10 mm = 8.2 AA + CRC = 34.1	CRC = 92.8 AA 10 mm+ = NR AA <10 mm = NR AA + CRC = 71.7
Lucoq 2022^81^ (development)	FIT (undefined) Anaemia (iron deficiency, severe anaemia, low TSAT anaemia) Other symptoms (undefined)	Machine learning	FIT + anaemia = 0.806 (NR) FIT + symptoms = 0.842 (NR)	NR	NR	NR	NR
Lue 2020^86^ (development)	FIT (≥20 μg/g) Faecal Calprotectin	NR	NR for individual outcomes	CRC = 93.75 AA = 82 CRC + AA = 85.5	CRC = 43.3 AA = 44.4 CRC + AA = 46.1	CRC = 6.4 AA = 13.6 CRC + AA = 20	CRC = 99.4 AA = 98.85 CRC + AA = 95.3
Mahadavan 2012^70^ (development)	Age Sex Colonocyte DNA Mean red cell volume CEA Rectal bleeding FOBT^c^	Logistic regression	Final model = 0.88 (0.84–0.92) Excl. unreliable samples = 0.9 (0.86–0.93) Excl. palpable patients = 0.84 (0.78–0.9)	NR	NR	NR	NR
Malagon 2019^34^ (development; RAID-CRC)	FIT (10 μg Hb/g of faeces) Eubacteria (EUB) P stomatis (PTST) B fragilis (BCTF) B thetaiotaomicron (BCTT)	Machine learning (four methods, neural network, logistic regression, gradient boosting tree, random forest)	CRC + AA = 0.84 (0.73–0.94)	CRC + AA = 80 (NR)	CRC + AA = 90 (NR)	CRC + AA = 70 (NR)	CRC + AA = 94 (NR)
Mowat 2016^71^ (PPV)	FHb (FIT: any numerical result greater than zero) Faecal Calprotectin (unclear cut-off)	NA	NA	CRC FHb and/or FC 50+ μg/g = 100 FHb and/or FC 200+ μg/g = 100 HRA FHb and/or FC 50+ μg/g = 92.7 FHb and/or FC 200+ μg/g = 85	CRC FHb and/or FC 50+ μg/g = 20.3 FHb and/or FC 200+ μg/g = 35.4 HRA FHb and/or FC 50+ μg/g = 20.3 FHb and/or FC 200+ μg/g = 35.1	CRC FHb and/or FC 50+ μg/g = 4.7 FHb and/or FC 200+ μg/g = 5.7 HRA FHb and/or FC 50+ μg/g = 6.3 FHb and/or FC 200+ μg/g = 6.9	CRC FHb and/or FC 50+ μg/g = 100 FHb and/or FC 200+ μg/g = 100 HRA FHb and/or FC 50+ μg/g = 97.9 FHb and/or FC 200+ μg/g = 97.6
Parente 2012^36^ (PPV)	Combinations of: FIT (100 ng/ml) Faecal Calprotectin Pyruvate kinase (M2-PK) At least one test must be positive for further investigation.	NA	NA	CRC FIT + FC = 90.9 (78.8–96.4) FIT + M2-PK = 91.5 (80.1–96.6) FC + M2-PK = 95.7 (85.7–98.8) FIT + FC + M2-PK = 95.7 (85.7–98.8) ACN FIT + FC = 75.8 (67.3–82.7) FIT + M2-PK = 71.2 (62.9–78.2) FC + M2-PK = 82.8 (75.1–88.4) FIT + FC + M2-PK = 86.1 (78.8–91.1)	CRC FIT + FC = 35.9 (29.7–42.6) FIT + M2-PK = 57.1 (50.6–63.2) FC + M2-PK = 26.4 (20.9–32.6) FIT + FC + M2-PK = 24.1 (18.8–30.2) ACN FIT + FC = 37.2 (29.6–45.6) FIT + M2-PK = 66.9 (58.9–73.9) FC + M2-PK = 26.9 (20.3–34.8) FIT + FC + M2-PK = 26.2 (19.7–34.1)	CRC FIT + FC = 22.9 (17.3–29.7) FIT + M2-PK = 30.1 (23.1–38) FC + M2-PK = 22.1 (16.9–28.2) FIT + FC + M2-PK = 21.5 (16.5–27.6) ACN FIT + FC = 50.6 (43.2–57.9) FIT + M2-PK = 65.7 (57.6–73) FC + M2-PK = 49.5 (42.7–56.3) FIT + FC + M2-PK = 50.2 (43.5–56.9)	CRC FIT + FC = 94.9 (87.7–98) FIT + M2-PK = 97.1 (92.7–98.9) FC + M2-PK = 96.6 (88.5–99.1) FIT + FC + M2-PK = 96.3 (87.5–98.9) ACN FIT + FC = 64.5 (53.5–75.4) FIT + M2-PK = 72.3 (64.2–79.1) FC + M2-PK = 64.4 (51.6–75.4) FIT + FC + M2-PK = 68.5 (55.2–79.3)
Rodriguez-Alonso 2015^40^ (Development; FAST score)	Age Sex FIT (≥10 μg/g faeces)	Logistic regression (internal validity assessed by split sampling)	ACN = 0.79 (0.76–0.84)	Score ≥5 = 75.9 (67.8–82.9)	Score ≥5 = 72 (68.8–74.9)	NR	NR
Turvill 2018^43^ (development)	FIT (varied from ≥2 to ≥12 μg Hb/g) Faecal Calprotectin (varied from ≥10 to ≥239 μg/g) Combinations of the tests include number of times ran and cut-offs	NR	Two FIT ≥2μgHb/g faeces + two FC ≥10 μg/g = 0.887 (0.828–0.946)^a^	91.7	85.8	25.6	99.5
Widlak 2017^44^ (development)	FIT (≥7 μg Hb/g) Faecal Calprotectin (≥50 μg Hb/g)	NR	CRC + HGD = 0.95 (NR) Adenoma = NR	CRC + HGD = 84 (NR) Adenoma = 69 (NR)	CRC + HGD = 93 (NR) Adenoma = 56 (NR)	CRC + HGD = 41 (NR) Adenoma = 15 (NR)	CRC + HGD = 99 (NR) Adenoma = 94 (NR)
Widlak 2018^45^ (development)	Model 1 FIT (≥3 μg Hb/g) Faecal Calprotectin (cut-off unclear) Model 2 FIT (≥3 μg Hb/g) Volatile organic compounds	Bayesian logistic regression (Internal validation by cross-validation)	Model 1 CRC = 0.91 (0.86–0.96) HRA = 0.69 (0.59–0.79) All adenomas = 0.6 (0.54–0.94) Model 2 CRC = 0.86 (0.77–0.94)	Model 1 CRC = 80 (66–93) HRA = 93 (81–100) Adenomas = 86 (79–93) Model 2 CRC = 80 (66–93)	Model 1 CRC = 93 (91–95) HRA = 25 (21–29) Adenomas = 26 (22–30) Model 2 CRC = 89 (87–93)	Model 1 CRC = 43 (31–55) HRA = 6 (4–8) Adenomas = 19 (15–23) Model 2 CRC = NR	Model 1 CRC = 99 (97–100) HRA = 99 (96–100) Adenomas = 90 (85–95) Model 2 CRC = 99 (97–100)
Withrow 2022^60^ (development)	FIT (≥2 or 10 μg Hb/g) Age Sex Blood tests (Hb, platelets, white cell count, MCH, MCV, serum ferritin, and CRP)	Logistic regression	Model a (FIT continuous) = 0.91 (0.87–0.95) Model b (FIT and blood tests dichotomous) = 0.93 (0.91–0.96) Model c (FIT spline) = 0.94 (0.92–0.96)	Model a = 93.8 (85–97.5) Model b = 93.5 (88.2–96.6) Model c = 92.1 (86.4–95.5)	Model a = 45.9 (44.7–47.1) Model b = 90.1 (89.6–96.6) Model c = 91.5 (91.1–91.9)	Model a = 1.7 (1.4–2.2) Model b = 7.4 (6.2–8.7) Model c = 8.4 (7.1–9.9)	Model a = 99.9 (99.6–99.9) Model b = 99.9 (99.9–100) Model c = 99.9 (99.9–100)

CRC = Colorectal Cancer; AA = Advanced Adenoma; HRA = High Risk Adenoma; ACN = Advanced Colorectal Neoplasia; NR = Not Reported; NA = Not Applicable; CI = Confidence Interval; AUC = Area Under the Curve; CEA = Carcinoembryonic Antigen; CIBH = Change in Bowel Habit; FIT = Faecal immunochemical test; BMI = Body Mass Index; MCH = Mean cell haemoglobin; CRP = C-reactive protein; HGD = High grade dysplasia; HRA = High Risk Adenoma; MCV = Mean Corpuscular volume; MCH = Mean Corpuscular Haemoglobin.

a Most accurate model presented.

b FAST score calculation increases with increasing value of FIT (0 μg/g, 0.6841 if 1–19 μg/g, 2.824 if 20–199 μg/g and 4.184 if ≥200 μg/g.

c Undefined, assumed to be guaiac.

d Assumed represents standard error.

**Fig. 2:**
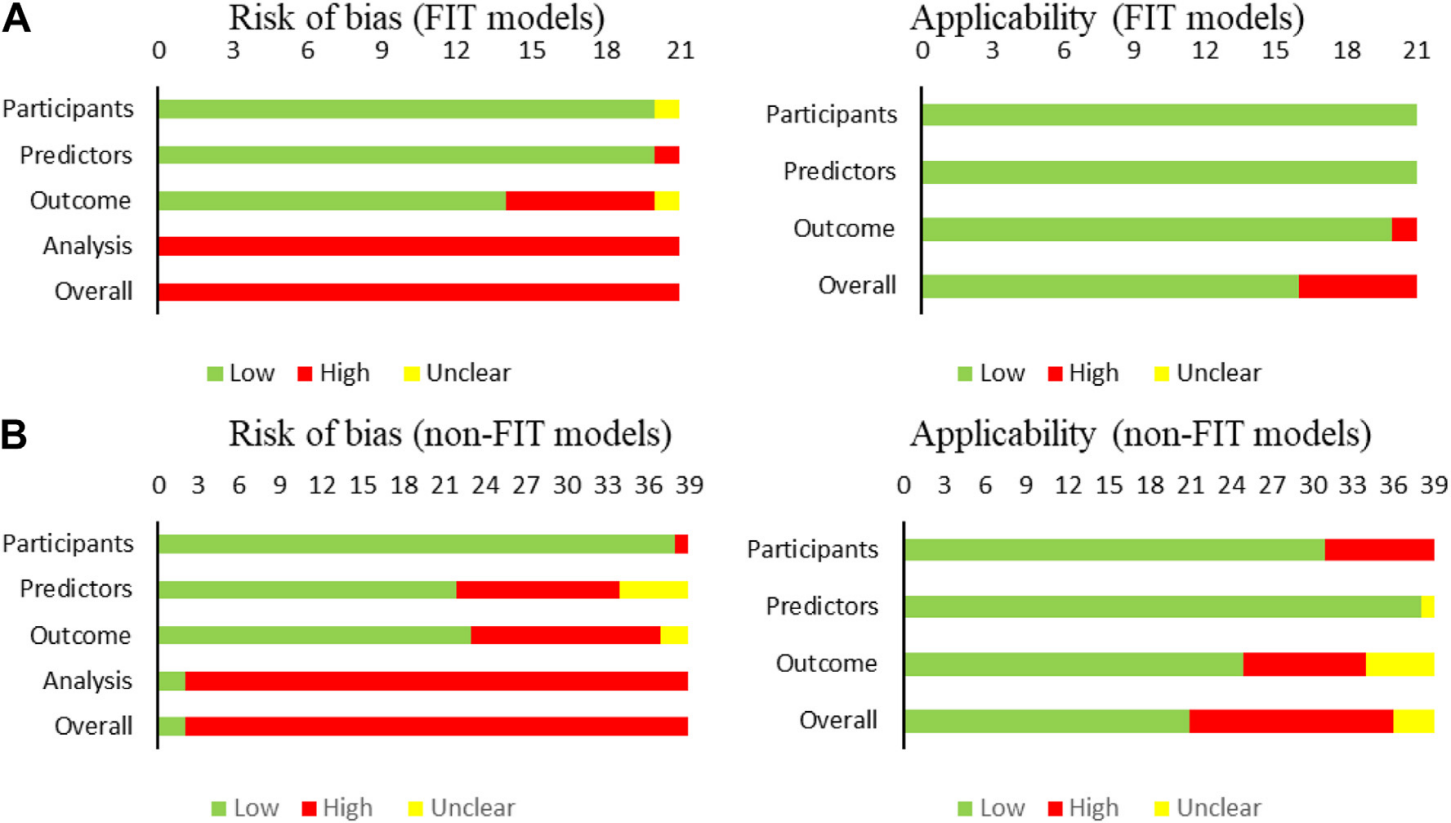
Risk of bias (left) and applicability (right) for **A.** Predictive model studies including FIT **B.** Predictive models not including FIT. Two models included in FIT are gFOBT.

The cut-off considered positive for FIT varied between studies (Table 2). One study classed any result above zero μg/g of faeces as positive^71^; another used a cut-off of 0.2 μg/ml,^32^ Eleven studies utilised a cut-off between 2 and 25 μg/g of faeces for a positive FIT result.^19^^,^^34^^,^^40^^,^^43^, ^44^, ^45^, ^46^^,^^60^^,^^63^^,^^67^^,^^86^ One study assessed four different analytical machines, with a positive FIT varying between machines (2–50 μg/g of faeces).^68^ Three studies of the FAST score (an equation based on FIT, age and sex) used different FIT cut-off values.^18^^,^^49^^,^^85^ One study categorised patients by their FIT result between <10 and >400 μg/g of faeces.^51^ The final FIT study assessed a cut-off 100 ng/ml.^36^ All studies including FIT/gFOBT as a variable were rated as high in the risk of bias. This was generally due to a lack of reporting of adequate calibration statistics (Fig. 2A).

#### FIT models assessing CRC

**Fig. 3:**
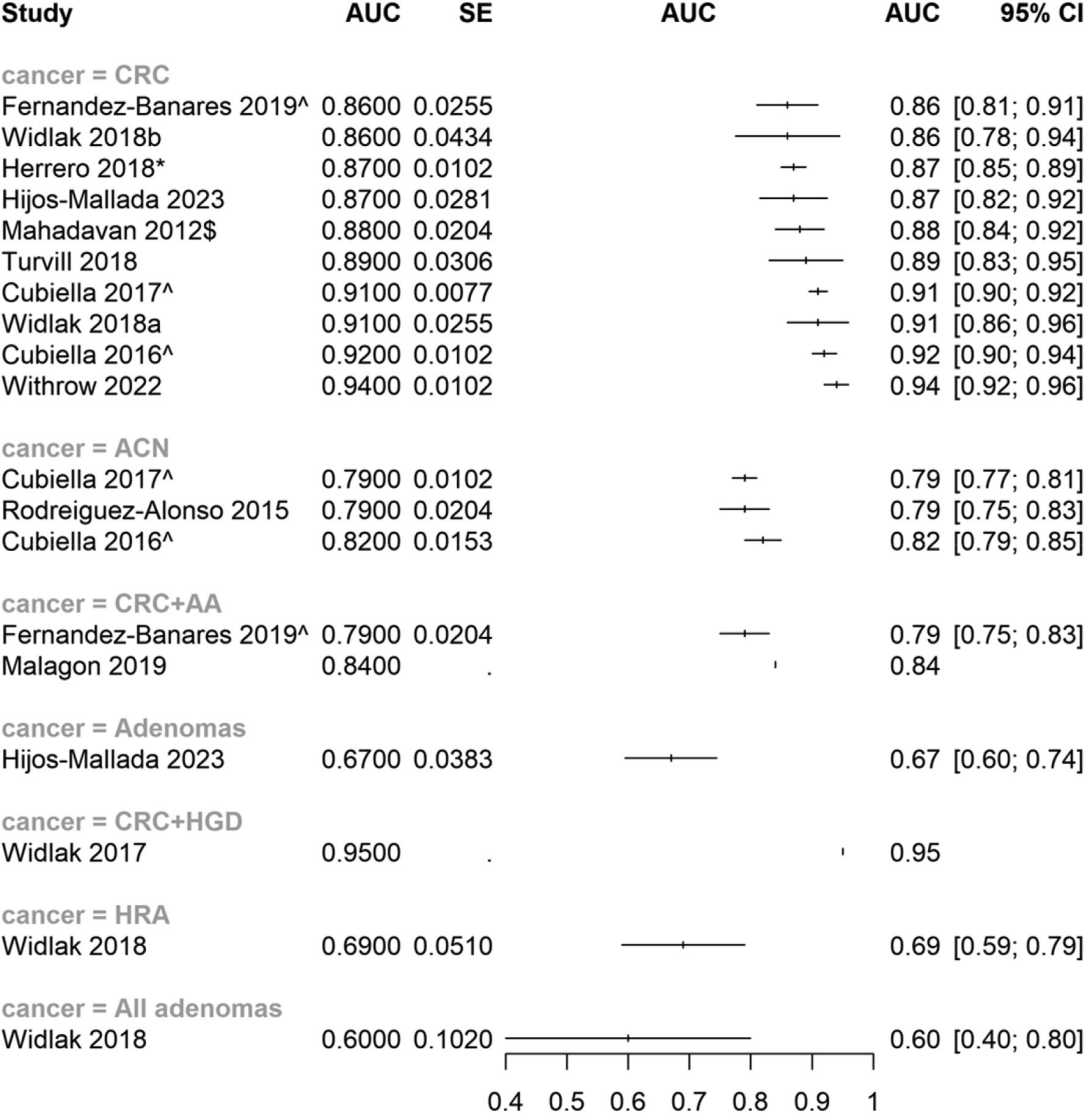
Forest plot (unweighted) of the area under the curve (AUC) and 95% confidence intervals (CI) of included studies assessing models that included FIT as a variable, subgroup is by outcome aimed to predict. Where models were validated, these scores are used in the forest plot. $denotes the model used gFOBT, not FIT. ˆdenotes a development and validation model; ∗denotes a validation only model. If no denotation, the model was development only. Studies that do not have confidence intervals did not report dispersion data. Widlak 2018a for CRC combined FIT and FC; Widlak 2018b for CRC combined FIT and volatile organic compounds. Abbreviations: AUC = Area Under the Curve; CI = Confidence Interval; CRC = Colorectal Cancer; ACN = Advanced Colorectal Neoplasia; AA = Advanced Adenoma; HGD = High Grade Dysplasia; HRA = High Risk Adenoma.

Ten of the models including FIT (or gFOBT) assessed CRC and reported measures of discrimination.^18^^,^^19^^,^^34^^,^^43^^,^^45^^,^^49^^,^^60^^,^^63^^,^^65^^,^^70^ Overall, these showed good discriminatory ability for CRC identification (i.e. AUC ≥0.8; see Fig. 3).

The most commonly reported model (n = 5) utilised FIT, age and sex (FAST) to produce a score that is assessed against a threshold (e.g. >2.12) for the prediction of both CRC and for can, separately (which is reported below). The FAST score showed good discriminatory ability for CRC when externally validated (AUC = 0.91).^18^ Further external validation showed similar results (AUC = 0.87).^49^ Three studies performed some form of further validation; these three studies reported similar levels of accuracy (i.e. sensitivity and specificity), but did not report measures of discrimination.^30^^,^^46^^,^^85^ All of these studies were rated high for risk of bias, mainly due to statistical concerns; for example, lack of calibration and selection of variables being based on univariate analysis. The case was similar for all studies that reported models including FIT, with no study being rated as low overall for risk of bias and analysis concerns being the major driver of this (see Fig. 2).

Two further models were also externally validated: COLONOFIT^63^ and COLONPREDICT.^19^ COLONOFIT, which used the maximum value and number of values above 4 μg Hb/g of FIT across three samples, in addition to age, smoking status and history of previous colonoscopy, showed good discrimination for CRC (validation AUC = 0.86). COLONPREDICT, which uses FIT, demographics, symptoms, and blood tests, also suggested good discrimination for CRC (validation AUC = 0.92). COLONPREDICT and the FAST score were reported to be more accurate at predicting CRC than the English National Institute for Health & Care Excellence (NICE) Guideline 12 (NG12)^49^ and Clinical Guideline 27 (CG27)—the NICE guideline for suspected cancer that preceded NG12.^30^^,^^49^

Ayling and colleagues (2021)^46^ also provided some validation of the ColonFlag score, an artificial intelligence learning algorithm, which was originally developed in an asymptomatic population.^87^, ^88^, ^89^, ^90^ They suggested that combining it with FIT could improve the sensitivity but discrimination and calibration were not reported.

Four studies reported on the combination of FIT/gFOBT and other biomarkers.^60^^,^^65^^,^^70^^,^^75^ One study obtained a high discrimination value for CRC (AUC = 0.94) by including haemoglobin, platelets, white cell count, Mean Corpuscular Haemoglobin (MCH), MCV, serum ferritin, and CRP markers, in addition to FIT.^60^ One other study reported on the combination of FIT and transferrin, but only reported accuracy measures (PPV = 20.4% for CRC).^32^ Another study assessed the combination of FIT, transferrin, lactoferrin and FC, showing good discriminatory ability (AUC = 0.87), however, this was not validated.^65^ One study that utilised a mixture of demographics, other biomarkers (colonocyte DNA, Mean Corpuscular Volume (MCV), Carcinoembryonic antigen (CEA)), rectal bleeding and gFOBT showed good discrimination for CRC (AUC = 0.88).

FIT combined with faecal calprotectin had high AUC for CRC, using either two samples from both tests (AUC = 0.89)^43^ or a single sample from each test (AUC = 0.91),^45^ but neither study provided either internal or external validation. Seven studies, reported varying results for accuracy when combining FIT with faecal calprotectin alone or with other variables (see Table 2).^36^^,^^43^, ^44^, ^45^^,^^67^^,^^71^^,^^86^ Three studies combining FIT and haematological tests such as anaemia/iron deficiency and thrombocytosis reported PPVs for CRC in the range 4%–9%.^51^^,^^67^^,^^68^

#### FIT models assessing CRC and ACP/ACN or colorectal neoplasia alone

**Fig. 4:**
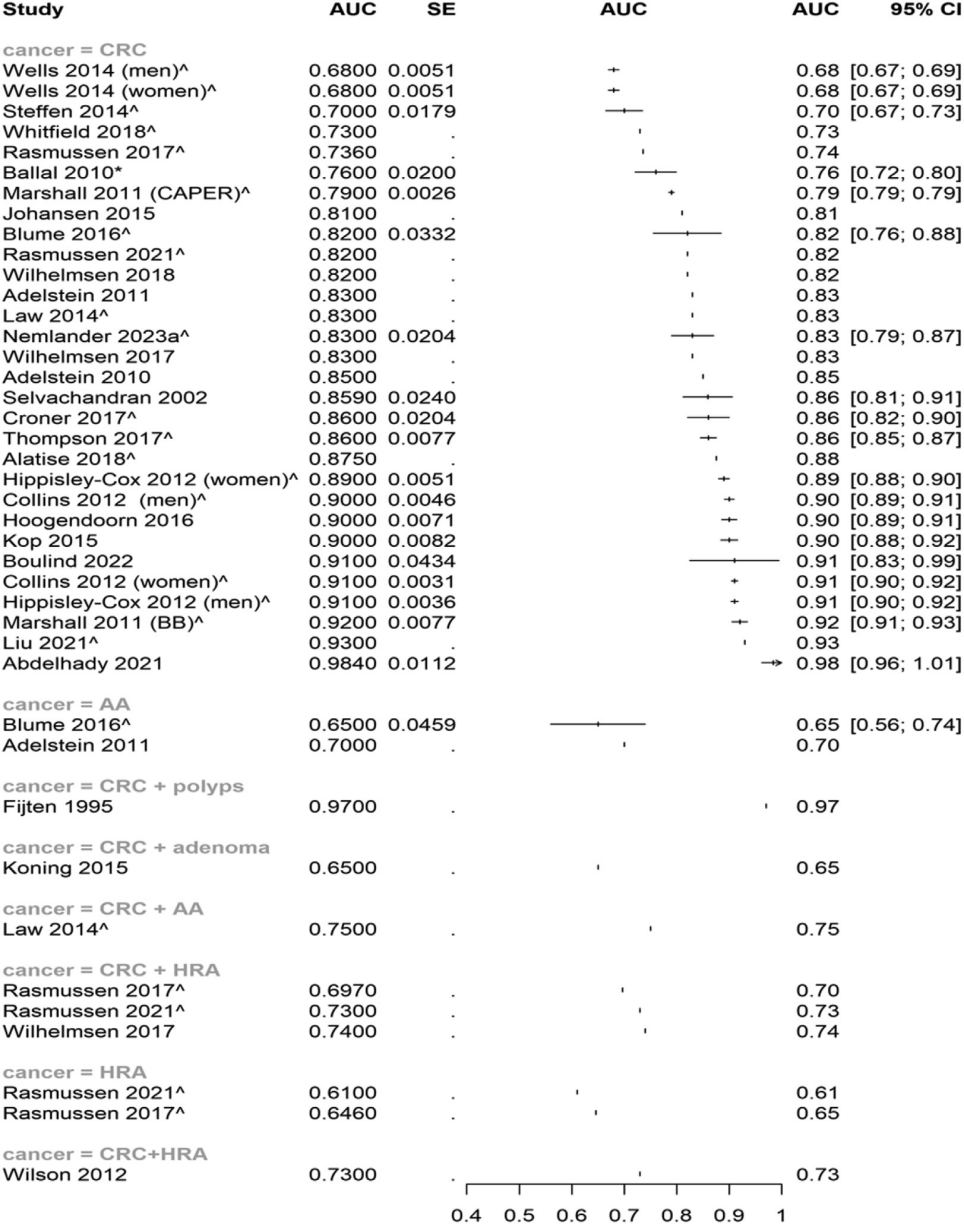
Forest plot (unweighted) of the area under the curve (AUC) and 95% confidence intervals (CI) of included studies assessing models that did not include FIT as a variable, subgroup is by outcome aimed to predict. Where models were validated, these scores are used in the forest plot. ˆdenotes a development and validation model; ∗denotes a validation only model. If no denotation, the model was development only. Studies that do not have confidence intervals did not report dispersion data. Abbreviations: AUC = Area Under the Curve; CI = Confidence Interval; CRC = Colorectal Cancer; AA = Advanced Adenoma; HRA = High Risk Adenoma.

Eight studies reported the discriminatory ability of FIT and other variables to assess CRC combined with other outcomes (e.g. advanced adenoma; AA) or such outcomes alone (e.g. ACN; see Fig. 4).^18^^,^^19^^,^^34^^,^^40^^,^^44^^,^^45^^,^^63^^,^^65^

The FAST score was originally developed for ACN, and it showed some discriminatory ability (AUC = 0.79)^40^; when externally validated this discriminatory ability was maintained (AUC = 0.79).^18^ Similar accuracy measures were obtained in these studies when using a cut-off score >4.5 for the outcome of CRC and HRA.^46^ Similar results for COLONPREDICT were observed when assessing the outcome of ACN (validation AUC = 0.82).^19^ COLONOFIT had a similar discriminatory ability for the outcome of CRC combined with advanced adenoma (AA), (validation AUC = 0.79).^63^

One study utilised machine learning methods to develop a model using bacterial biomarkers in addition to FIT for prediction of CRC and advanced adenoma (AA) combined, suggesting good discrimination (AUC = 0.84).^34^ However, the study was not internally or externally validated. Another biomarker study utilising FIT, FC, transferrin and lactoferrin showed poor discrimination (AUC = 0.67) for the prediction of adenomas.^65^

Assessing for the combined outcome of CRC and high-grade dysplasia, the combination of FIT and faecal calprotectin had high discriminatory ability (AUC = 0.95),^44^ but the study included only 430 people and did not report internal or external validation. One further study reported the combination of FIT with FC had poor discriminatory ability for HRA (AUC = 0.69) and all adenomas (AUC = 0.6)^45^ The combination of FIT and FC had a varying reported PPVs for outcomes such as ACN and HRA (PPV range = 6.3–22.9%).^36^^,^^71^^,^^86^

### Non-FIT models

The remaining 39 studies did not include FIT/gFOBT and assessed models that utilised a mixture of symptoms, haematological tests, medical history, and demographical information.^27^, ^28^, ^29^^,^^31^^,^^33^^,^^35^^,^^37^, ^38^, ^39^^,^^41^^,^^42^^,^^47^^,^^48^^,^^50^^,^^52^, ^53^, ^54^, ^55^, ^56^, ^57^, ^58^, ^59^^,^^61^^,^^62^^,^^64^^,^^66^^,^^69^^,^^72^, ^73^, ^74^, ^75^, ^76^, ^77^, ^78^, ^79^, ^80^^,^^82^ Of these, 18 were development studies,^27^^,^^28^^,^^33^^,^^39^^,^^41^^,^^50^^,^^52^, ^53^, ^54^^,^^59^^,^^62^^,^^64^^,^^69^^,^^73^, ^74^, ^75^^,^^79^^,^^82^ three were validation studies,^38^^,^^47^^,^^61^ ten presented both development and validation,^29^^,^^48^^,^^55^, ^56^, ^57^, ^58^^,^^66^^,^^72^^,^^76^^,^^78^ and eight were classified as PPV studies.^31^^,^^35^^,^^37^^,^^42^^,^^62^^,^^77^^,^^78^^,^^80^ For further details of the results, see Table 3.

**Table 3:** Results from studies that did not include faecal blood tests as a variable but combined two or more other variables.

Study (type of study)	Predictors (final model)	Modelling method	AUC (95% CI)	Sensitivity % (95% CI)	Specificity % (95% CI)	PPV % (95% CI)	NPV % (95% CI)
Abdelhady 2021^75^ (development)	Golgi protein-73 CEA	Unclear	0.984 (0.963–1.007)	93.33 (NR)	98.33 (NR)	96.6 (NR)	96.7 (NR)
Adelstein 2010^27^ (development)	Age Sex Previous colonoscopy (10 yrs) Diverticular disease NSAID/aspirin use Mucus Abdominal pain Anaemia	Logistic regression, backwards elimination (Internal validation, bootstrapping)	0.85 (NR)^a^	NR	NR	NR	NR
Adelstein 2011^28^ (development)	Age Sex Education level Previous colonoscopy (10 yrs) NSAIDs/aspirin use Smoking status Previous polyps IBS Rectal bleeding Mucus Anaemia Fatigue	Logistic regression, backwards elimination	CRC = 0.83 (NR)^a^ AA = 0.7 (NR)^a^	NR	NR	NR	NR
Alatise 2018^29^ (development and validation)	Weight loss (last 6 months) Change in bowel habit	logistic regression	Development = NR Validation = 0.875 (NR)	89% (NR; Symptom score of 2)	83% (NR; Symptom score of 2)	NR	NR
Ballal 2010^61^ (validation; Selva Score)	WNS derived from a colorectal symptom questionnaire. Works by adding assigned weightages to reported main symptoms of bleeding per rectum and CIBH. Weights change with age and presence/no presence of other symptoms (See Selvachandran 2002).	NR	0.76 (SE = 0.02)	WNS score 40+: 93 (NR) WNS score 50+: 88.2 (NR) WNS score 60+: 70.4 (NR) WNS score 70+: 59.1 (NR)	WNS score 40+: 31.7 (NR) WNS score 50+: 47.9 (NR) WNS score 60+: 64 (NR) WNS score 70+: 77.4 (NR)	WNS score 40+: 7.2 (NR) WNS score 50+: 8.8 (NR) WNS score 60+: 10 (NR) WNS score 70+: 12.9 (NR)	NR
Blume 2016^76^ (development and validation)	Alpha-1-acid glycoprotein 1 (AACT) Cathepsin D (CATD) CEA Complement component 3 (CO3) Complement component 9 (CO9) Macrophage migration inhibitory factor (MIF) P-selection glycoprotein ligand 1(PSGL) Seprase (SEPR)	Machine learning (support vector, with sigmoid kernel–default parameters)	CRC Development = 0.85 (NR)^a^ Validation = 0.82 (0.75–0.88)^a^ AA Development = 0.77 (NR) Validation = 0.65 (0.56–0.74)	80 (NR)	68 (NR)	NR	NR
Boulind 2022^62^ (development)	Volatile organic compounds x 13 Unclear which compounds are used in the final model	Artificial Neural Network 3 volatile organic compound analyses: Selected Ion Flow Tube Mass Spectrometry (SIFT-MS) Field Asymmetric Ion Mobility Spectrometry (FAIMS) Gas Chromatography Mass Spectrometry (GC–MS)	CRC SIFT-MS = 0.872 (0.794–0.949) FAIMS = 0.855 (0.724–0.986) GCMS = 0.913 (0.825–1) CRC + polyps SIFT-MS = 0.662 (0.602–0.723) FAIMS = 0.664 (0.591–0.734) GCMS = 0.896 (0.802–0.966) CRC vs polyps SIFT-MS = 0.813 (0.704–0.922) FAIMS = 0.855 (0.732–0.977) GCMS = 0.896 (0.796–0.996)	CRC: SIFT-MS = 0.778 (0.524–0.936) FAIMS = 0.889 (0.653–0.986) GCMS = 0.833 (0.586–0.964) CRC + polyps SIFT-MS = 0.6 (0.5–0.694) FAIMS = 0.429 (0.332–0.529) GCMS = 0.878 (0.752–0.953) CRC vs polyps SIFT-MS = 0.722 (0.465–0.903) FAIMS = 0.722 (0.465–0.903) GCMS = 0.889 (0.633–0.986)	CRC: SIFT-MS = 0.78 (0.733–0.822) FAIMS = 0.778 (0.524–0.936) GCMS = 0.815 (0.7–0.901) CRC + polyps SIFT-MS = 0.605 (0.543–0.664) FAIMS = 0.872 (0.794–0.928) GCMS = 0.882 (0.726–0.967) CRC vs polyps SIFT-MS = 0.759 (0.655–0.844) FAIMS = 0.889 (0.653–0.986) GCMS = 0.871 (0.702–0.964)	NR	NR
Collins 2012^47^ (validation; QCancer)	*Men* Age Family history of GI cancer Abdominal pain Appetite loss Rectal bleeding Weight loss Anaemia Change in bowel habit Alcohol consumption	NR directly QCancer = Cox's proportional hazards model	Internal validation = 0.91 (0.9–0.91) External validation Multiple imputation model = 0.918 (0.913–0.923) Complete cases = 0.901 (0.892–0.910)	NR	NR	NR	NR
	*Women* Age Family history of GI cancer Abdominal pain Appetite loss Rectal bleeding Weight loss Anaemia		Internal validation = 0.89 (0.88–0.9) Complete cases = 0.909 (0.903–0.915)	NR	NR	NR	NR
Croner 2017^48^ (development and validation)	Alpha-1-acid glycoprotein (A1AG) CEA Complement 9 (CO9) Dipeptidyl peptidase IV (DPPIV) Macrophage migration inhibitory factor (MIF) Pyruvate kinase isozyme M2 (PKM2) Transferrin receptor protein (TFRC)	Machine learning	Development = 0.89 (NR) Validation = 0.86 (0.82–0.9)	Development = 0.8 (NR) Validation = 0.8 (NR)	Development = 0.87 (NR) Validation = 0.83 (NR)	Validation = 36.5 (NR)	Validation = 97.1 (NR)
Ellis 2005^31^ (PPV)	Rectal bleeding + one or more of the following: Chang in bowel habit Perianal symptoms Abdominal pain	NA	NA	Bleeding + CIBH = 100 Bleeding + CIBH (loose) = 91 Bleeding + no perianal symptoms = 64 Bleeding + CIBH + abdominal pain = 55	Bleeding + CIBH = 55 Bleeding + CIBH (loose) = 32 Bleeding + no perianal symptoms = 78 Bleeding + CIBH + abdominal pain = 44	Bleeding + CIBH = 9.2 Bleeding + CIBH (loose) = 12.1 Bleeding + no perianal symptoms = 11.1 Bleeding + CIBH + abdominal pain = 9 (+no pain = 9.6)	NR
Ewing 2016^83^ (PPV)	Change in bowel habit Rectal bleeding (incl. GI, unclassified and melena) Weight loss (incl. anorexia) Anaemia (combined iron deficiency anaemia and other anaemias) Abdominal pain	NA	NA	NR	NR	CIBH + bleeding = 13.7 (2.1–54.4) CIBH + abdominal pain = 1.5 (0.8–2.6) CIBH + Anaemia = 2.9 (1–8.4) Bleeding + abdominal pain = 12.2 (1.8–51.2) Bleeding + Anaemia = 2.9 (1.2–6.9) Weight loss + Anaemia = 5.6 (0.7–33) Abdominal pain + Anaemia = 4.2 (1.6–2.4)	NR
Fijten 1995 (development)	Age Sex Blood mixed with stool Change in bowel habit (excl. constipation)	Logistic regression	0.97 (NR)	Cut-off = 0.042 100 (NR)	Cut-off = 0.042 90 (NR)	Cut-off = 0.042 26 (NR)	Cut-off = 0.042 0 (NR)
Hamilton 2005^77^ (PPV)	Constipation Diarrhoea Rectal bleeding Weight loss Abdominal pain Abdominal tenderness Abnormal rectal exam Haemoglobin	NA	NA	NR	NR	PPV >5% Abdominal tenderness + weight loss = 6.4 Abnormal rectal exam + diarrhoea = 11 + rectal bleeding = 8.5 + weight loss = 7.4 + abdominal tenderness = 5.8 Hb < 10 g dl + abdominal pain = 6.9 + abdominal tenderness = >10	NR
Hippisley-Cox 2012^66^ (development and validation; QCancer)	Split by male and female: Age Alcohol status (Males only) Change in bowel habit (Males only) Family history of GI cancer Hb < 11 g/dl in last year Rectal bleeding Abdominal pain Appetite loss Weight loss	Cox's proportional hazards model	Development = NR Validation Female = 0.89 (0.88–0.9) Male = 0.906 (0.899–0.913)	Provided at risk thresholds for top percentage risk score: 10% = 70.6 5% = 56.4 1% = 24.6	Provided at risk thresholds for top percentage risk score: 10% = 90.1 5% = 95.1 1% = 99	Provided at risk thresholds for top percentage risk score: 10% = 1.5 5% = 2.4 1% = 5.2	Provided at risk thresholds for top percentage risk score: 10% = 1.5 5% = 2.4 1% = 5.2
Hoogendoorn 2016^50^ (development)	Age Sex Medication: medication prescribed, dosage. ATC scheme Consultation codes: code of symptoms and/or diagnoses during the consultation visit, ICPC coding (Dutch version) Referrals: to secondary care Lab results: any form of lab measurement performed by the GP, or received from an external lab. Consultation notes: uncoded notes entered by GP (in Dutch)	Machine learning (Completed using various methods: bag of words (1) topic modelling with oversampling (2) separate topic modeling for two classes (3) topic modeling beyond consultation code (4) coding using ICPC (5) coding using UMLS (6) topic modelling can use one of the following bayesian approaches: Latent dirichlet allocation (LDA) Hierarchical dirichlet processes (HDP))	Average AUCs obtained from 5 fold cross validation Age, sex consultation code, medication, referrals, lab result, and text/consultation notes—UMLS coding Regular counts = 0.896 (0.882–0.910)^a^ Temporal patterns plus regular counts = 0.900 (0.886–0.914)^a^	NR	NR	NR	NR
Johansen 2015^69^ (development)	Age Sex CEA Serum YKL-40	Logistic regression	0.81 (NR)	NR	NR	NR	NR
Koning 2015^52^ (development)	Age Sex Hypertension Abdominal pain	Logistic regression	CRC + Adenoma = 0.65 (NR)	NR	NR	NR	NR
Kop 2015^53^ (development)	Based on model Non-temporal model Temporal model All (non-temporal + temporal + age/sex) Knowledge driven (Bristol–Birmingham equation + age/sex) Age/sex only	Machine learning (Four methods used, logistic regression, random forest, support vector modelling and classification and regression trees; 5 fold cross-validation)	Random forest provided the most accurate model Knowledge driven = 0.896 (0.88–0.912)^a^	NR	NR	NR	NR
Kop 2016^54^ (development)	Temporal pattern with succession relationships (s). Top five predictors: Drugs for constipation Iron deficiency anaemia Lipid modifying agents (s) Drugs for constipation Age Drugs for acid related disorders (s) Drugs for constipation	Machine learning (Three methods used, logistic regression, random forest, and classification and regression trees; 5 fold cross-validation)	Logistic regression Age/sex, Bristol–Birmingham equation + like category = 0.891 (0.879–0.903)^a^ Extra step of “various steps of the regular pipeline” did not change AUC.	NR	NR	NR	NR
Law 2014^33^ (development)	Age Sex Ethnicity Education level Smoking status Family history of colorectal polyps Family history of colitis Family history of any cancer Family history of colorectal cancer Medication history—NSAID, aspirin, anti-diabetic, and iron tablets Symptom history—abdominal pain, pain on defection, CIBH, jelly-like stool, anal irritation, itch and swelling General symptoms—loss of appetite, weight loss, tiredness	Logistic regression (internal validation by cross-validation)	CRC Adjusted model = 0.83 (cross-validation = 0.79) Score based model = 0.83 (cross-validation = 0.83) CRC + AA Adjusted model = 0.76 (cross-validation = 0.73) Score based model = 0.76 (cross-validation = 0.75)	Score CRC 5+ = 99.1 10+ = 86.4 15+ = 47.4 17+ = 34.2 CRC + AA 5+ = 85.7 10+ = 39.4 12+ = 22.9	Score CRC 5+ = 15.6 10+ = 63.9 15+ = 90.9 17+ = 96.3 CRC + AA 5+ = 49.3 10+ = 89.7 12+ = 96.9	Score CRC 5+ = 13 10+ = 23.3 15+ = 39.7 17+ = 54.2 CRC + AA 5+ = 26.1 10+ = 44.5 12+ = 60.6	Score CRC 5+ = 99.3 10+ = 97.5 15+ = 93.2 17+ = 92 CRC + AA 5+ = 94.3 10+ = 87.6 12+ = 85.7
Liu 2021^55^ (development and validation)	Biomarkers: Septin 9 (SEPT9) Syndecan 2 (SDC2) Secreted frizzled-related protein 2 (SFRP2)	Logistic regression	Development = 0.931 (NR) Validation = 0.927 (NR) Testing = 0.937 (NR)	Testing = 94.1 (NR)	Testing = 89.2 (NR)	NR	NR
Marshall 2011^56^ (development and validation; Bristol–Birmingham equation and CAPER score)	Constipation Diarrhoea Change in bowel habit Abdominal pain Weight loss Rectal bleeding Hb concentration Mean cell volume	Logistic regression	Development = 0.83 (0.82–0.84) Validation = 0.92 (0.91–0.94) CAPER score^b^ Development = 0.91 (0.89–0.93) Validation = 0.79 (0.79–0.8)	NR	NR	NR	NR
Nemlander 2023a^78^ (development and validation)	Unclear; 16 most important variables were: Iron deficiency anaemia other diseases of anus and rectum Abdominal and pelvic pain Other anaemias Haemorrhoids and perianal venous thrombosis CIBH number of consultations during the year before the index date other and unspecified non-infective gastroenteritis and colitis Melaena Haemorrhage of anus and rectum gastrointestinal haemorrhage, unspecified Benign neoplasm of colon, rectum, anus and anal canal Nausea and vomiting Other diseases of digestive system Other and unspecified soft tissue disorders, not elsewhere classified Essential primary hypertension	Stochastic gradient boosting applied to classification decision trees	Validation = 0.83 (0.79–0.87)	73.3 (NR)	83.5 (NR)	NR	NR
Nemlander 2023b^84^ (PPV; validation)	Validation of Swedish Colorectal Cancer Risk Assessment Tool (SCCRAT) developed by Ewing 2016: CIBH rectal bleeding Weight loss abdominal pain anaemia	Logistic regression	NR	NR	NR	PPVs > 2.5% All ages and sex CIBH + rectal bleeding = 7.8 (1.9–26.9) CIBH + abdominal pain = 3.1 (1.9–5) CIBH + anaemia = 3.5 (1.8–6.6) rectal bleeding + abdominal pain = 10.7 (1.5–48) rectal bleeding + anaemia = 4.2 (1.6–10.4) weight loss + anaemia = 3.8 (0.8–15.9)	NR
Norrelund 1996^35^^,^^c^ (PPV)	Age Change in bowel habit Patient belief symptoms due to cancer	Logistic regression	NR	CRC Study 1 Age >69 yrs + CIBH = 44 (NR) Due to cancer + CIBH = 22 (NR) Study 2 new bleeders Age >69 yrs + CIBH = 15 (NR) Due to cancer + CIBH = 0 (NR) Study 2 new or changed bleeders Age >69 yrs + CIBH = 23 (NR) Due to cancer + CIBH = 5 (NR)	CRC Study 1 Age >69 yrs + CIBH = 94 (NR) Due to cancer + CIBH = 97 (NR) Study 2 new bleeders Age >69 yrs + CIBH = 88 (NR) Due to cancer + CIBH = 95 (NR) Study 2 new or changed bleeders Age >69 yrs + CIBH = 88 (NR) Due to cancer + CIBH = 96 (NR)	CRC Study 1 Age >69 yrs + CIBH = 56 (NR) Due to cancer + CIBH = 58 (NR) Study 2 new bleeders Age >69 yrs + CIBH = 13 (NR) Due to cancer + CIBH = 0 (NR) Study 2 new or changed bleeders Age >69 yrs + CIBH = 24 (NR) Due to cancer + CIBH = 14 (NR)	CRC Study 1 Age >69 yrs + CIBH = 90 (NR) Due to cancer + CIBH = 87 (NR) Study 2 new bleeders Age >69 yrs + CIBH = 85 (NR) Due to cancer + CIBH = 87 (NR) Study 2 new or changed bleeders Age >69 yrs + CIBH = 87 (NR) Due to cancer + CIBH = 86 (NR)
Payne 1983^37^ (PPV)	CEA Leucocyte adherence inhibition	NA	NA	91 (NR)	68 (NR)	54 (NR)	95 (NR)
Rai 2008^38^ (validation; Selva score)	Weighted numerical score (WNS); See Selvachandran 2002.	NR	NR	WNS cut off 40 = 95.2 (NR) WNS cut off 50 = 78.3 (NR) WNS cut off at 60 = 77.1 (NR) WNS cut off at 70 = 63.9 (NR)	WNS cut off 40 = 36.3 (NR) WNS cut off 50 = 52.7 (NR) WNS cut off at 60 = 68.5 (NR) WNS cut off at 70 = 82.7 (NR)	WNS cut off 40 = 8.5 (NR) WNS cut off 50 = 10.7 (NR) WNS cut off at 60 = 13.2 (NR) WNS cut off at 70 = 18.9 (NR)	NR
Rasmussen 2017^82^ (development)	Age Sex ccfn containing 5-methylcytosine DNA (5 mC) CEA	Logistic regression (internal validation by cross-validation)	CRC = 0.736 (NR) CRC + HRA = 0.697 (NR) HRA = 0.646 (NR)	Specificity at 70^a^ CRC = 61.5 CRC + HRA = 57.1 HRA = 48	NR	NR	NR
Rasmussen 2021^79^ (development)	All models include: Age Sex CRC only: Angiopoietin 2 (ANGPT2) Arginase 1 (ARG1) Colony stimulation factor 1 (CSF-1) Galectin 9(Gal-9) Inducible T-cell costimulatory ligand (ICOSLG) Interleukin 8 (IL8) HRA only: T-cell surface glycoprotein 28 (CD28) CRC or HRA: ICOSLG IL8	Logistic regression (internal validation by cross-validation)	CRC only = 0.82 (NR) HRA only = 0.61 (NR) CRC or HRA = 0.73 (NR)	Sensitivity at varying specificities Specificity 70 CRC only = 58 (NR) HRA only = 43 (NR) CRC or HRA = 54 (NR) Specificity 80 CRC only = 39 (NR) HRA only = 31 (NR) CRC or HRA = 36 (NR) Specificity 90 CRC only = 18 (NR) HRA only = 13 (NR) CRC or HRA = 18 (NR)	NR	NR	NR
Selvachandran 2002^41^ (Development; Selva score)	Weighted numerical score Age Sex Blood per rectum Change in bowel habit Tenesmus, urgency, and incomplete emptying Perianal symptoms Abdominal symptoms Weight loss Loss of appetite Tiredness Family history (unspecified) Relevant medical history	NR	0.859 (SE = 0.024)	40+ = 99 (NR) 50+ = 91 (NR) 60+ = 76 (NR) 70+ = 70 (NR)	40+ = 46 (NR) 50+ = 62 (NR) 60+ = 78 (NR) 70+ = 88 (NR)	NR	NR
Simpkins 2017^42^ (PPV)	Combinations stratified by age (only those with 2 symptoms are reported here) Weight loss Abdominal pain Rectal bleeding Change in bowel habit Anaemia	NA	NA	≥40 years old + weight loss + abdominal pain = 32.6 (20.5–47.5) ≥50 years old + rectal bleeding + abdominal pain = 12.8 (6–25.2) <50 years old + rectal bleeding + CIBH = 10.6 (4.6–22.6) <50 years old + rectal bleeding + weight loss = 12.8 (6–25.2) <50 years old + rectal bleeding + anaemia = 2.2 (0.4–11.3)	≥40 years old + weight loss + abdominal pain = 87.1 (85.5–88.5) ≥50 years old + rectal bleeding + abdominal pain = 82 (80.2–83.7) <50 years old + rectal bleeding + CIBH = 87.5 (86–88.9) <50 years old + rectal bleeding + weight loss = 91.4 (90.1–92.6) <50 years old + rectal bleeding + anaemia = 93.6 (92.5–94.7)	≥40 years old + weight loss + abdominal pain = 5.4 (3.3–8.9) ≥50 years old + rectal bleeding + abdominal pain = 1.7 (0.8–3.7) <50 years old + rectal bleeding + CIBH = 2 (0.9–4.7) <50 years old + rectal bleeding + weight loss = 3.8 (1.7–7.9) <50 years old + rectal bleeding + anaemia = 0.8 (0.1–4.5)	≥40 years old + weight loss + abdominal pain = 98.3 (97.5–98.8) ≥50 years old + rectal bleeding + abdominal pain = 97.5 (96.6–98.1) <50 years old + rectal bleeding + CIBH = 97.6 (96.7–98.2) <50 years old + rectal bleeding + weight loss = 97.7 (96.9–98.3) <50 years old + rectal bleeding + anaemia = 97.5 (96.7–98.1)
Stapley 2017^80^ (PPV)	Diarrhoea Abdominal pain Rectal bleeding Change in bowel habit Constipation Nausea/vomiting Rectal mass Raised inflammatory markers (erythrocyte sedimentation rate, CRP, or plasma viscosity)	Logistic regression (Assessed strength of associations between clinical features and CRC)	NA	NR	NR	PPVs >5% Rectal mass + bleeding = 17 + CIBH = 6.3 + constipation = 6.1 + diarrhoea = 5.1 + abdominal pain = 7 + low Hb = 5.6 + raised inflammatory markers = 7 Rectal bleeding + constipation = 5.8 + low Hb = 13 + low mean red cell volume = 8 CIBH + diarrhoea = 6.1 + low Hb = 5.1 Constipation + low mean red cell volume = 5.1	NR
Steffen 2014^72^ (development and validation)	Age Sex BMI Diabetes Ever had CRC screening Smoking status Alcoholic drinks per day	Cox's proportional hazards regression	Development = 0.73 (0.72–0.74) Validation = 0.7 (0.66–0.73)	NR	NR	NR	NR
Thompson 2017^57^ (development and validation)	Age Sex Change in bowel habit Rectal bleeding Abdominal pain/discomfort Perianal symptoms Rectal mass Abdominal mass Iron deficiency anaemia Change in weight (loss or gain)	Logistic regression	Development = 0.87 (0.85–0.88) Validation = 0.86 (0.84–0.87)	23⋅9% when the probability of bowel cancer was over 50% 38⋅3% with a 20% probability of bowel cancer	99⋅3% when the probability of bowel cancer was over 50% 97⋅1% with a 20% probability of bowel cancer	NR	NR
Wells 2014^73^ (development)	Split by male and female: Age Ethnicity BMI Red meat intake per day (male only) Aspirin use (male only) Physical activity hours per day (male only) NSAID use (female only) Oestrogen use (female only) Pack years smoking History of diabetes Years of education Alcoholic drinks per day Family history of CRC Multivitamin use	Logistic regression (10-fold cross validation)	Men = 0.681 (0.669–0.694) Women = 0.679 (0.665–0.692) Results presented only after internal validation.	NR	NR	NR	NR
Whitfield 2018^58^ (development and validation)	Age Indication of bleeding Minimum mean corpuscular Hb Minimum ferritin Median white blood cell count Median platelet count	Logistic regression	Development = 0.779 (NR) Validation = 0.727 (NR)	NR	NR	NR	NR
Wilhelmsen 2017^39^ (development)	Model 1 (full model) Age Sex AFP Ca19-9 CEA Galectin-3 CyFra21-1 Ferritin Hs-CRP TIMP-1 Model 2 (reduced model) Age Sex CEA CyFra21-1 Ferritin Hs-CRP	Logistic regression	Model 1 CRC = 0.84 (NR) CRC + HRA = 0.76 (NR) Model 2 CRC = 0.83 (NR) CRC + HRA = 0.74 (NR)	CRC 90 80 70 60 CRC + HRA 90 80 70 60 Reported at varying sensitivities; values are reported in line with sensitivity	CRC 33 50 66 75 CRC + HRA 48 66 81 89	CRC 25 29 34 37 CRC + HRA 18 23 31 41	CRC 93 91 90 88 CRC + HRA 97 96 95 95
Wilhelmsen 2018^74^ (development)	Model 1 (full model) Age Sex Pepsinogen 2 Huma epidermis antigen 4 (HE4) hs-CRP CEA Ferritin CyFra21-1 Model 2 (reduced model) Age Sex HE4 CEA CyFra21-1	Logistic regression	Model 1 = 0.84 (NR) Model 2 = 0.82 (NR)	NR	NR	NR	NR
Wilson 2012^59^ (development)	Age Sex Weight loss Blood in stools Harder stools Anal pain/soreness White blood cell count Smoking history Alcohol history Hypertension Serum Matrix Metalloproteinase 9 (MMP9)	Logistic regression (two-stage process; cut-off of 0.05 on predicted probability of neoplasia, all patients who were positive from this process re-entered for a second stage using the same cut-off)	Stage 1 = 0.77 (NR) Stage 2 = 0.73 (NR)	Stage 1 = 79% Stage 2 = NR Combined stage 1 & 2 = 79%	Stage 1 = 63% Stage 2 = NR Combined stage 1 & 2 = 70%	NR	NR

CRC = Colorectal Cancer; AA = Advanced Adenoma; HRA = High Risk Adenoma; ACN = Advanced Colorectal Neoplasia; NR = Not Reported; NA = Not Applicable; CI = Confidence Interval; AUC = Area Under the Curve; CEA = Carcinoembryonic Antigen; NSAIDs = Non-steroidal anti-inflammatory drugs; IBS = Irritable Bowel Syndrome; CIBH = Change in Bowel Habit; GI = Gastrointestinal; BMI = Body Mass Index; MCH = Mean cell haemoglobin; CRP = C-reactive protein; SE = Standard Error.

a Most accurate model presented.

b CAPER development is from original dataset and validation is the THIN database used in Marshal 2011.

c Presents two studies, second study refers to new or changed bleeders.

#### Non-FIT models assessing CRC

Twenty-seven studies reported discriminatory ability of models including a diverse range of variables with the aim of predicting CRC (see Fig. 4).^27^^,^^28^^,^^29^^,^^33^^,^^39^^,^^41^^,^^47^^,^^48^^,^^50^^,^^53^^,^^56^^,^^57^^,^^58^^,^^59^^,^^61^^,^^62^^,^^66^^,^^69^^,^^73^^,^^74^^,^^75^^,^^76^^,^^78^^,^^79^^,^^82^

#### Biomarker-based models

Twelve studies reported on models that included one or more tests from routine blood panels or biomarkers.^37^^,^^39^^,^^48^^,^^55^^,^^59^^,^^62^^,^^69^^,^^74^, ^75^, ^76^^,^^79^^,^^82^ The most commonly reported biomarker was carcinoembryonic antigen (CEA; n = 8, three of which had a case–control design).^37^^,^^39^^,^^48^^,^^69^^,^^74^, ^75^, ^76^^,^^82^ One study assessed the combination of Golgi protein-73 and CEA and reported high discriminatory ability for CRC (AUC = 0.98); but the study included only 90 people and had a case–control design.^75^ Two studies reported development of models, with no validation, for combinations of other biomarkers (see Table 3).^79^^,^^82^ Three further studies developed and externally validated various biomarker combinations, without including sex and age as factors.^48^^,^^55^^,^^76^ All three showed good discriminatory ability for CRC in Danish (AUC = 0.82 and 0.86),^48^^,^^76^ Chinese (AUC = 0.94)^55^ and patients. Finally, one study that only provided accuracy measures, suggested combining CEA and leucocyte adherence inhibition had a high PPV (54%) for CRC.^37^ All of these studies were rated as high risk of bias, mainly due to concerns regarding analysis (e.g. lack of appropriate calibration). Four other studies reported varying accuracy in development models using multiple different biomarkers combined with age and sex but did not externally validate results.^39^^,^^69^^,^^74^^,^^82^

#### Demographics, symptoms, and medical history-based models

The Bristol–Birmingham (BB) equation was developed and validated using the UK THIN primary care database, identifying multiple symptoms and providing one of the highest discrimination values for CRC (AUC = 0.92).^56^ However, there were some concerns regarding the identification and applicability of the outcome in the risk of bias assessment. The BB equation was validated within the study and compared against the CAPER (Cancer Prediction in Exeter) score, suggesting it was superior in identifying CRC (validation AUC = 0.79).^56^

One study developed and validated a model using change in bowel habit (CIBH) and weight loss, although patients must have presented with rectal bleeding.^29^ Only the validation AUC was reported; this suggested good discrimination for CRC (0.88). Another study that utilised a combination of demographics, symptoms and iron deficiency anaemia suggested good discriminatory ability for CRC in development (AUC = 0.87) and validation (AUC = 0.86) cohorts.^57^ However, there were concerns regarding the handling of missing data in the analysis, which were coded as absent/missing and meant the predictive value of symptoms may have been overestimated.

A study in Australian patients developed and validated a model using demographics, lifestyle, and past medical history factors for prediction of CRC and colon and rectal cancers separately.^72^ While the model showed moderate discrimination for all three outcomes in development, and the CRC and colon models maintained adequate discrimination after validation (AUC = 0.7 and 0.72, respectively), the discrimination for rectal cancer was less than adequate after validation (AUC = 0.64).

Two development studies combined medical history, demographics, symptoms and haematological tests, providing good discriminatory ability for CRC (AUC ≥0.83).^27^^,^^28^ Another development model utilised age and sex with CIBH (excluding constipation) and the presence of blood in stool with age and sex and demonstrated good discriminatory ability for CRC (AUC = 0.97).^64^ An issue of applicability was present in this study; rectal bleeding was a pre-requisite for inclusion.^64^ Only one of these four studies provided some form of validation (internal).^27^

#### Scored-based models

Three papers reported development^41^ and validation^38^^,^^61^ of a weighted numerical score (also known as the Selva score), which combines demographics, history and symptoms, for CRC prediction. The results suggested a good to moderate discriminatory ability (AUC development = 0.86,^41^ validation = 0.76)^61^ in a secondary care setting. A similar score-based model—incorporating age, indication of bleeding, minimum MCH, minimum ferritin, median WBC, and median platelet count–was reported to have adequate discrimination after validation (AUC = 0.73), but was only available as a conference abstract so detail was limited.^58^ Each of these studies were rated as having a high risk of bias, mainly due to reporting of analysis. One study (of the Selva score) also had concerns regarding patient and outcome applicability.^41^

The QCancer for CRC risk was developed and validated using the UK QResearch database.^47^^,^^66^ This algorithm, included demographics, history, and symptoms, with some factors only considered for males and some only for females (Table 3).^47^^,^^66^ Results suggested good discriminatory ability for CRC (AUC = 0.91 for men and 0.89 women). Net benefit analysis showed QCancer to be better than an “investigate all” or “investigate none” approach.^47^ Additionally, the validation study was rated as low risk of bias, only one of two studies to attain this rating.^47^^,^^73^ The other study that attained a low risk of bias was similar to the QCancer algorithm, utilising historical variables to assess male and female risk separately; however, only internal validation was performed and the AUC indicated less than adequate discrimination (0.68).^73^ Another study developed a score-based algorithm with an array of factors (see Table 3), reporting good discriminatory ability for CRC (AUC = 0.83).^33^

#### Machine learning models using GP records

Four studies applied machine learning techniques to medical notes (e.g. GP records).^50^^,^^53^^,^^54^ All three models, which were developed in Dutch patients’ records, showed good discrimination for CRC (AUC range = 0.81–0.9). One of these studies utilised the BB equation to aid the development of their most accurate model.^53^ Another study explicitly focused on non-metastatic CRC using a case–control study design (Swedish cancer registry) to create a model using multiple symptoms and medical history, reporting good discriminatory ability (validation AUC = 0.83).^78^ There were major concerns regarding these studies and how they identified predictors and outcomes. All studies utilised medical records from their respective countries; three from the Netherlands,^50^^,^^53^^,^^54^ and one from Sweden,^78^ which could limit their.

#### PPV studies

Eight studies assessed PPV for CRC of combinations of symptoms or haematological tests (Table 3).^31^^,^^35^^,^^37^^,^^42^^,^^62^^,^^77^^,^^78^^,^^80^ The most commonly considered symptoms were rectal bleeding (n = 5),^31^^,^^42^^,^^77^^,^^80^^,^^83^ CIBH (n = 5),^31^^,^^35^^,^^42^^,^^80^^,^^83^ and abdominal pain (n = 4).^31^^,^^42^^,^^77^^,^^83^ The PPVs varied depending on the combinations of symptoms, with highest PPVs for symptoms alone being for rectal mass and bleeding (17% for CRC).^80^ All of these studies were rated as high risk of bias, due to analysis concerns and issues of predictor selection^80^^,^^83^ and outcome definitions.^35^^,^^77^^,^^83^ Nemlander and colleagues 2023b^84^ validated the symptom combinations used by Ewing and colleagues,^83^ in a separate Swedish population with a focus on non-metastatic CRC and found similar PPVs, for example CIBH and rectal bleeding PPVs were 7.8% and 13.7%, rectal bleeding and abdominal pain were 10.7% and 12.2%, respectively.

#### Non-FIT models assessing CRC and ACP/ACN or colorectal neoplasia alone

Eleven studies reported discriminatory ability of varying models for the identification of other outcomes (e.g. AA) alone or in combination with CRC (see Fig. 4).^28^^,^^33^^,^^39^^,^^52^^,^^55^^,^^59^^,^^64^^,^^76^^,^^79^^,^^82^

One study assessed the combination of several biomarkers for prediction of AA and reported poor discriminatory ability after validation (Table 3; AUC = 0.65).^76^ There were concerns about how the predictors where determined. Four other studies combined demographic information (e.g. age) and/or various biomarkers.^39^^,^^76^^,^^79^^,^^82^ Poor discriminatory ability was observed when assessing only AA (AUC = 0.65)^76^ and HRAs (AUC = 0.61–0.65).^79^^,^^82^ Discriminatory ability improved when attempting to predict CRC and HRA (AUC = 0.7–0.76).^39^^,^^79^^,^^82^ However, poor results were observed for the combination of age, sex, hypertension and abdominal pain for the prediction of CRC and adenoma (AUC = 0.65).^52^ One study assessed a single biomarker (serum matrix metalloproteinase 9) with age, sex, symptoms, white blood cell count, lifestyle factors and hypertension, and reported adequate discrimination for the prediction of colorectal neoplasia (defined as presence of adenocarcinoma or HRA) (internal validation AUC = 0.73),^59^ but did not undertake external validation.

One development study combined medical history, demographics, symptoms and haematological tests, providing and adequate discrimination ability for AA (AUC = 0.7).^28^ A similar study, utilising demographics, history (e.g. family, medication), and symptoms, also reported adequate ability for CRC and AA combined (AUC = 0.76).^33^ One study, including hypertension and abdominal pain, had poor discrimination for CRC and adenoma prediction (AUC = 0.65).^52^

One study reported an adjusted model (AUC = 0.73; cross-validation) and a score-based model (AUC = 0.75; cross-validation) combining demographics, family and medical history, and symptoms for the prediction of CRC and AA.^33^ Calibration was lacking. The highest recorded discriminatory ability for a combined outcome (in this case polyps and CRC) was reported by combining age, sex, blood mixed in stool and CIBH (AUC = 0.92).^64^ However, there were concerns regarding the participants, outcome identification, analysis, and the applicability of the study.

## Discussion

This systematic review identified 62 studies assessing risk prediction models for CRC and/or ACP in symptomatic patients. Of these, 23 assessed models containing tests for blood in stool (21 FIT-based; one gFOBT-based) and 39 assessed non-FIT/gFOBT based models. Twenty-one of the 62 studies were conducted solely in primary care populations. Overall, the evidence suggests prediction models including FIT consistently have good accuracy and discriminatory ability (i.e. AUC > 0.8).

Some models that did not include FIT also had high levels of accuracy and discrimination, but this was not a consistent finding. In addition, eight of the studies assessing non-FIT predictive models had a case–control study design,^62^^,^^75^, ^76^, ^77^, ^78^, ^79^, ^80^ which could have overestimated model usefulness. Models, irrespective of whether they included FIT, generally had higher discriminatory ability for CRC than for CRC combined with ACP or ACP alone. For example, the FAST score (FIT, age, and sex) reported AUC of 0.91 for CRC compared to 0.79 for advanced neoplasia in external validation.^18^ Of note, only two studies in this review had a low risk of bias; neither of those models included FIT.^47^^,^^73^ Moreover, several of the studies (n = 15) which reported AUC or similar measures did not report measures of dispersion. The majority of these were non-FIT models (n = 13).

FIT-based models varied in what other variables they included and, by and large, the number of included variables was unrelated to model performance. This, and the heterogeneity in the variables included, means that it is not possible to recommend to those developing such models on variables they might consider including (with the exception of sex, which is discussed further below). Some FIT-based models (such as the FAST score) contained a small number of simple additional variables which, other issues notwithstanding, would suggest they could fairly easily be implemented in routine clinical practice. In comparison, others, such as COLONPREDICT, which reported similar discriminatory ability for CRC (AUC = 0.92) to the FAST score, utilised eleven variables. Furthermore, the COLONOFIT model required three stool samples for calculation, which would require considerable effort to manage in routine clinical practice, including complex safety-netting should patients not provide all samples required. Simple combinations of tests also showed promising results; for example, FIT and faecal calprotectin was explored in several studies and showed some promise as a predictive test, with good discriminatory ability for CRC and HRA. However, no validation was performed in these studies.^43^, ^44^, ^45^

While FIT-based models generally performed well, there were variations in the cut-off for defining a “positive” FIT across the models, with no single cut-off most favoured. Sometimes this was because of limitations in the analytical performance of the test (e.g. unable to detect below a certain level). The lack of certainty around the optimum cut-off for FIT in models reported to date, and concerns around comparability of different tests in the symptomatic setting,^91^ has implications for comparison of findings across studies and settings, though this is somewhat averted by studies using FIT as a continuous variable in their modelling. It also has implications for future implementation in that it was not possible to reach a conclusion on which cut-off should be preferred in practice; this remains to be established.

A number of models utilising biomarkers combined with FIT or gFOBT (n = 5)^34^^,^^36^^,^^45^^,^^65^^,^^70^ or other factors excluding FIT (n = 13)^37^^,^^39^^,^^48^^,^^55^^,^^58^^,^^59^^,^^62^^,^^69^^,^^74^^,^^76^^,^^79^^,^^80^^,^^82^ were identified. However, most of these studies had no form of validation. Commonly, such biomarker studies assessed two or more biomarkers either alone or in conjunction with age and sex. The main concern with these models was that many of the biomarkers assessed are not readily available in a clinical setting, having not progressed beyond the research arena. For example, one biomarker model included Septin 9 (SEPT9), Syndecan 2 (SDC2) and Secreted frizzled-related protein 2 (SFRP2), which are not routinely available.^55^ The feasibility of using such models is currently low.

Many models included sex as a predictive factor while some, such as the QCancer for CRC risk, went further and utilised different variables for males and females.^66^ The QCancer model was the only model to present a net-benefit of using the model: this suggested it was more accurate than the (unrealistic) scenarios of “test nobody” or “test everyone”. The attraction of sex-stratified models is clear given the higher incidence rate of CRC in males than females^1^ but the acceptability to patients, health professionals and health service decision-makers of different referral algorithms by sex requires investigation.

An important factor to consider when evaluating the potential utility of a risk prediction model is the setting for potential use. For example, three models that applied machine learning techniques to medical notes were developed in Dutch patients’ records and, although the studies showed good discriminatory ability, it is not known if these models are applicable in other healthcare systems, where medical documentation styles may differ.^50^^,^^53^^,^^54^ Such models require further external validation to demonstrate their generalisability to other data outside that used to develop the model. Related to this, few of the studies reported the ethnicity of the individuals in the population(s) in which they developed or validated their models. Therefore, an important caveat on the conclusions of the review is that, while some models perform well (and are validated), it is generally uncertain how they would perform in a population with a very different ethnic make-up.

In this review we also included studies where the outcome measure was PPV for combinations of variables; the rationale for this was our desire to provide a comprehensive overview of the current state of the evidence-base. All of these studies were classed as high risk of bias as PPV (a measure of diagnostic accuracy) is not considered to be an adequate outcome measure for risk prediction models, though is widely used by clinicians and policy makers. These studies were included because previous UK guidance for investigation of symptomatic patients has been based on PPVs.^92^ Studies without FIT presented an array of different symptom combinations and identified some combinations with a high predictive value (e.g. rectal mass and bleeding had a PPV of 17% in one study).^80^ Those which included FIT generally combined it with other blood or stool test results (e.g. faecal calprotectin, iron deficiency) and mostly reported high PPVs. Given these findings, and the fact that some of these other test results would either be available routinely as part of primary care blood panels or could be assessed in stool samples, future work assessing calibration and validation of models including FIT, other standard blood/stool test results and, potentially, combinations of symptoms, is warranted.

This review was conducted using a comprehensive search strategy, developed in combination with an information specialist, and utilised rigorous systematic review methodology. By focussing on risk prediction models published up to 2023, it both updates and extends a past systematic review on this topic (which included papers published to March 2014)^93^ and the systematic review that informed the 2022 British Society of Gastroenterology/Association of Coloproctology of Great Britain and Ireland guidance on use of FIT in symptomatic patients, which focussed on diagnostic accuracy studies.^94^ However, there are some limitations. Firstly, we excluded non-English language studies. While this, in theory, may have introduced some selection bias, research suggests that the chances of this are low.^95^ Secondly, we did not perform data extraction in blinded duplicate: this could increase data extractions errors. However, a second reviewer assessed the data extraction for accuracy minimising or eliminating such error. Thirdly, studies utilising primary care databases/cancer registries to identify CRC diagnoses were considered eligible for inclusion unless it was explicitly stated that the study population included asymptomatic or screening patients. The rationale for this was two-fold: firstly, the review sought to be comprehensive and excluding these studies would have limited scope and introduced an element of selection bias and, secondly, in primary care, most CRCs are diagnosed through symptomatic services (even in settings with well-organised population-based screening programmes). However, it is possible these studies may have included a small proportion of asymptomatic patients. Fourthly, we included studies with a case–control design; while this was in order to be comprehensive, such studies may be more prone to bias and can over-estimate model usefulness. These limitations were reflected in the risk of bias assessment for the relevant studies. Also considered in the risk of bias assessment was the method of investigation for neoplasia. Method of identification for the outcome of interest (i.e. CRC and/or ACN) varied. While many studies utilised colonoscopy alone (n = 25), some studies utilised varying methods of identification (e.g. sigmoidoscopy; n = 20) or used a database/registry without providing clarification as to how the outcome was identified in those patients (n = 15). While colonoscopy would generally be considered gold-standard, studies with varying methods of identification were included to reflect real-world practice, but it is possible that model performance may have varied if colonoscopy had been used.

This review was undertaken within a programme of work (COLOFIT) intended to inform optimal use of a FIT-based strategy for managing referral of patients with possible CRC symptoms presenting to primary care in NHS England (https://fundingawards.nihr.ac.uk/award/NIHR133852). The review findings suggest several recommendations for future research on risk prediction models for colorectal neoplasia in symptomatic patients; while some of these will be addressed in COLOFIT, they have internationally applicability. While it may seem obvious, to rigorously evaluate the likely performance of a model, it should be assessed in the population that is the intended target of the algorithm (here, most often, primary care populations); secondary or tertiary care populations are generally enriched for CRC/ACP making models potentially non-generalisable to primary care populations. Ideally, the ethnic composition of the population should be reported. Adequate validation should be undertaken, at a minimum internal validation, though ideally external. Authors should report all available data, including calibration plots and measures of dispersion for AUC, and consider conducting a net-benefit analysis to assess likely model effectiveness and compare their model to existing pathways. If including FIT, if possible, authors should report performance for different cut-offs and, if including symptoms, understanding the predictive value of individual symptoms would be valuable. As is evident from this review, many models have now been developed. However, the lack of data on net-benefit in appropriate target populations and external validation is a significant impediment to their wider implementation. Finally, real world studies of the impact of the use of prediction models on clinical decision-making and patient outcomes are urgently required.^96^

The use of FIT in the symptomatic setting has significantly increased over recent years and, in some settings, guidance now advocates FIT for use in patients with features of possible CRC to guide referral for urgent investigation. This review shows that there is considerable promise for the use of risk prediction models, both FIT-based and non-FIT based, in identifying those most at risk of colorectal neoplasia. However, there are significant limitations in the evidence base, notably around the lack of net-benefit analysis and external validation, and the real-world impact of such algorithms is not yet understood.

## Contributors

James S Hampton (JSH) and Ryan PW Kenny (RPWK) co-authored the first draft of the review protocol, contributed to development of the search strategy, undertook the screening and selection of articles, extracted data, synthesised results and co-authored the first draft of the manuscript.

Claire Eastaugh (CE) and Catherine Richmond (CR) provided expertise in developing and performing the searches and approved final manuscript for submission.

Colin J Rees (CJR) had the idea for the review, secured funding, edited and approved review protocol, contributed to development of the search strategy, edited and approved final manuscript for submission.

William Hamilton (WH) had the idea for the review, secured funding, edited and approved review protocol, contributed to development of the search strategy, edited and approved final manuscript for submission.

Linda Sharp (LS) had the idea for the review, secured funding, edited and approved review protocol, contributed to development of the search strategy, arbitrated any conflicts in the study selection process, edited and approved final manuscript for submission.

JSH and RPWK accessed and verified the data. LS, CJR and WH made the decision to submit the manuscript for publication.

## Data sharing statement

All of the relevant data is contained within the manuscript and Supplementary material.

## Declaration of interests

JSH, RPK, CE, CR, WH declare no competing interests. CJR has received grant funding from ARC medical, Norgine. Medtronic, 3D Matrix solutions and Olympus medical. He was an expert witness for ARC medical and Olympus medical. LS holds grant funding from Medtronic and 3D Matrix.
