# Supplementary material for: The performance of FIT-based and other risk prediction models for colorectal neoplasia in symptomatic patients: a systematic review
Source: eClinicalMedicine. 2023 Sep 21;64:102204. doi: 10.1016/j.eclinm.2023.102204 (PMC10541467; doi:10.1016/j.eclinm.2023.102204)
Supplement: Supplementary material [file mmc1.pdf]

# A systematic review of the performance of risk prediction models for colorectal cancer and advanced neoplasia in symptomatic patients

Review methods were amended after registration. Please see the revision notes and previous versions for detail.

## Citation

James Hampton, Ryan Kenny, Catherine Richmond, Claire Eastaugh, Willie Hamilton, Linda Sharp, Colin Rees. A systematic review of the performance of risk prediction models for colorectal cancer and advanced neoplasia in symptomatic patients. PROSPERO 2022 CRD42022314710 Available from:
https://www.crd.york.ac.uk/prospero/display_record.php?ID=CRD42022314710

## Review question

Aim:

1) To identify and synthesise quantitative data on the contributions of FIT and other variables (utilised within existing risk prediction models) for the prediction of colorectal cancer (CRC) and/or advanced colorectal polyps (ACP) in symptomatic patients

Objectives:

1) To identify, and assess the performance, of models that predict the risk of CRC and/or ACP in symptomatic patients

2) To identify, and assess the performance of, FIT based models that predict the risk of CRC and/or ACP in symptomatic patients

## Searches

The search strategy will be developed subjectively and iteratively, based upon reporting methods and indexing of prediction and prognostic studies. The main prognostic study filter added to the content search strategy follows the study by Geersing. et.al. This is a validated filter attributed to the Cochrane Prognostic Group and is associated with the Dutch Cochrane Centre. The filter has been pre-validated by the authors, to confirm that it results in a highly sensitive search for retrieving hard to find prediction studies, and clinical decision rules. The main diagnostic study filter added to the search strategy is used by SIGN and has been adapted from the filter designed by the Health Information Research Unit of the McMaster University, Ontario.

The search strategy is designed to run on (OVID) MEDLINE initially, using a combination of MESH thesaurus headings and keywords pertaining to CRC, risk models and prognosis, with appropriate use of stemming for alternative word endings, alternative spellings and plurals. No restrictions will be applied according to language or country.

The following electronic databases will be used:

MEDLINE

Embase

Cochrane Library

Scopus

CINAHL

This topic is a rapidly evolving field of research it is likely that some relevant work will not yet be published in scientific journals therefore, grey literature sources (e.g., GoogleScholar), registers of trials/protocols (e.g., Clincialtrials.gov) and repositories for preprints (e.g., Open Science Framework) and abstracts will also be searched.

Forward and backward citation searching will be utilised and reference lists of any relevant systematic reviews will be reviewed. In addition, experts with the field will be consulted to ensure all eligible studies are included.

## Types of study to be included  [1 change]

Predictive modelling studies using randomised designs, prospective or retrospective cohort studies or cross sectional studies.

Studies that report the positive predictive value for combinations of predictors will also be considered

Studies will be excluded if they do not match the inclusion criteria and if:

No full text is available, however, abstracts that report full findings will be included. Where appropriate we will contact authors for data.

Non-English Language papers

Assess solely screening populations or populations undergoing surveillance. Studies containing both screening /surveillance and symptomatic will be eligible if the symptomatic group is reported separately.

Studies that are looking at prognostic factors in treatment or outcome of CRC (instead of disease risk)

Studies that focus only upon genetic variables.

Contain paediatric populations

## Condition or domain being studied

Colorectal cancer. Risk prediction models.

## Participants/population  [1 change]

Patients with symptoms suggestive of CRC. Any stage of CRC or type of ACP will be considered.

Studies performed in primary care that use primary care datasets/cancer registries to identify CRC diagnoses, will be included unless it is explicitly stated patients were asymptomatic or screening populations. The rationale for this is because in primary care the majority of CRCs are diagnosed through symptomatic services.

For studies recruiting patients from colonoscopy lists, hospital records or cancer units, or which use data/samples from commercial or publicly available datasets/repositories, the authors must state patients were symptomatic.

## Intervention(s), exposure(s)

All predictive models concerned with the prognostic ability of models containing FIT and/or other prognostic factors for the prediction of CRC and/or ACP in symptomatic patients.

## Comparator(s)/control

None

## Context

Studies from primary, secondary or tertiary care

## Main outcome(s) [1 change]

Ability to detect CRC, ACP* or both

*There are several terms that can be used to describe colorectal polyps with features that are high risk for malignancy, and we acknowledge that there is variation in the terms used. For the review, all studies that use any of following terms as outcomes will be accepted. All definitions of these terms, including but not limited to the definitions below, will be accepted:

Advanced serrated polyp—A serrated polyp of at least 10mm in size or containing any grade of dysplasia

Advanced adenomatous polyp (advanced adenoma)—An adenoma of at least 10mm in size or containing high-grade dysplasia

Advanced colorectal polyp (ACP)—includes both advanced serrated polyps and advanced adenomatous polyp

Advanced colorectal neoplasia (ACN) - This term has been used historically to describe the combination of advanced adenomas and colorectal cancers

## Additional outcome(s)

Not applicable

## Data extraction (selection and coding)

Study selection will be conducted in two stages, involving screening the citations identified by the search and identifying those meeting the inclusion criteria.

Firstly, citations will be exported from the Endnote library and into Rayyan, a web-based tool designed to speed up the process of screening and selection of studies. Title and abstract screening will be conducted independently in a blinded manner by two reviewers.

Secondly, studies that appear to meet the eligibility criteria will be exported back into Endnote and the full text papers will be obtained. Where full texts are not readily available locally, we will obtain the full text report using an inter-library loan. Full papers will be independently screened by two reviewers against the eligibility criteria. Any disagreements will be resolved by arbitration with a third reviewer.

A detailed description of the screening and selection process, including reasons of exclusion at full text stage will be documented using the guidelines in the PRISMA statement.

A data extraction form will be created based on the Checklist for critical Appraisal and data extraction for systematic Reviews of prediction Modelling Studies. A single reviewer will extract data from included studies; a second reviewer will check for accuracy.

Data extracted will include:

- Publication title and journal

- Study design

- Setting (e.g., primary care or secondary care)

- Participant information

 - Eligibility criteria

 - Recruitment methods

- Outcomes to be predicted

- Candidate predictors

- Sample size

- Missing data

- Model Development

- Model performance measures

- Model evaluation

- Results

- Interpretation and Discussion

Any additional information on the performance of models, such as the prognostic ability of models to predict multiplicity of polyps or non-advanced colorectal polyps will also be extracted where available.

## Risk of bias (quality) assessment

Each study will be assessed using the Prediction study Risk Of Bias Assessment Tool (PROBAST). One researcher will assess the risk of bias, with a second reviewer checking for accuracy. Any conflicts that cannot be resolved will be arbitrated with a third reviewer. The PROBAST toll assesses the risk of bias and applicability of prediction modelling studies across four domains:

- Participant selection

- Predictors

- Outcomes

- Analysis

Each domain is rated as low, high or unclear risk of bias by applying responses to signalling questions about the study in the tool and then an overall judgement of risk of bias is made using one of the following three categories:

- Low risk

- High risk

- Unclear risk

## Strategy for data synthesis [1 change]

Where possible we will meta-analyse the predictive performance of similar prediction models using statistical measures of predictive performance, discrimination and calibration.

Discrimination is a prediction model's ability to distinguish between patients developing and not developing the outcome of interest. This is commonly quantified by the concordance (C) statistic or the area under the receiver operating characteristic (AUROC).

Calibration is a prediction model's accuracy of predicted risk probabilities, indicating the extent to which expected outcomes (predicted from the model) and observed outcomes agree. If possible we will extract information regarding the total number of observed (O) and expected (E) events. The total O:E ratio provides a rough indication of the overall model calibration across the entire range of predicted risks. Additionally, when reported we will extract information summarising estimates of the calibration slope.

We will consider measures of accuracy (sensitivity and specificity). Sensitivity evaluates a model's ability to predict true positives, while specificity the true negatives.

For meta-analysis we will follow guidance by Debray and colleagues. Briefly, we will run a random-effects meta-analysis, using the REML estimation and the HKSJ correction. Additionally, we will include 95% prediction intervals. To assess the influence of each study on the overall effect size, a sensitivity analysis using the leave-one-out method will be utilised. included compared to when it is not included.

A narrative synthesis will also be presented. If a meta-analytical approach is not possible, we will follow the guidelines for Synthesis Without Meta-analysis. It is possible that the review will identify studies that utilise factors which are not routinely collected or used in clinical practice. As such, we will provide a narrative overview of which factors are commonly available and those that are not.

## Analysis of subgroups or subsets

Risk status of the patients is an important clinical consideration, which will be described narratively, and where possible a subgroup analysis will be provided. Additionally, we will consider further subgroup analyses by neoplasia outcome (CRC, ACN or ACP) and by study design (e.g., retrospective vs prospective).

## Contact details for further information

Dr James Hampton

james.hampton@nhs.net

## Organisational affiliation of the review

Newcastle University

## Review team members and their organisational affiliations

Dr James Hampton. Newcastle University

Dr Ryan Kenny. Newcastle University

Catherine Richmond. Newcastle University

Claire Eastaugh. Newcastle University

Professor Willie Hamilton. University of Exeter

Professor Linda Sharp. Newcastle University

Professor Colin Rees. Newcastle University

## Type and method of review

Meta-analysis, Narrative synthesis, Systematic review

## Anticipated or actual start date

03 January 2022

## Anticipated completion date [1 change]

31 October 2022

## Funding sources/sponsors

NIHR HTA Programme

## Grant number(s)

State the funder, grant or award number and the date of award

NIHR133852

## Conflicts of interest

## Language

English

## Country

England

## Stage of review

Review Ongoing

## Subject index terms status

Subject indexing assigned by CRD

## Subject index terms

Colonoscopy; Colorectal Neoplasms; Early Detection of Cancer; Humans

## Date of registration in PROSPERO

15 March 2022

## Date of first submission

11 March 2022

## Stage of review at time of this submission [1 change]

| Stage | Started | Completed |
|---|---|---|
| Preliminary searches | Yes | Yes |
| Piloting of the study selection process | Yes | Yes |
| Formal screening of search results against eligibility criteria | Yes | Yes |
| Data extraction | Yes | No |
| Risk of bias (quality) assessment | Yes | No |
| Data analysis | No | No |

## Revision note

The inclusion criteria was amended to provide increased clarity on which studies are to be included in the review.

*The record owner confirms that the information they have supplied for this submission is accurate and complete and they understand that deliberate provision of inaccurate information or omission of data may be construed as scientific misconduct.*

*The record owner confirms that they will update the status of the review when it is completed and will add publication details in due course.*

## Versions

15 March 2022

26 August 2022

# COLOFIT Search Strategies

Claire Eastaugh
Research Assistant Information Specialist
Evidence Synthesis Group
11/03/2022

## Contents

Totals ........ 2
MEDLINE ........ 3
EMBASE ........ 5
CINAHL ........ 7
Scopus ........ 9
Cochrane Libraries ........ 8

## Totals

Searches carried out on: 04/03/22

| DATABASE | NUMBER OF RESULTS |
|---|---|
| **MEDLINE** | 5227 |
| **EMBASE** | 11222 |
| **CINAHL** | 936 |
| **COCHRANE LIBRARIES** | 673 |
| **SCOPUS** | 6233 |
| | |
| **TOTAL BEFORE DE-DUPLICATION** | **24291** |
| **TOTAL AFTER DE-DUPLICATION** | **15614** |

## MEDLINE

Database(s): **Ovid MEDLINE® and Epub Ahead of Print, In-Process, In-Data-Review & Other Non-Indexed Citations, Daily and Versions** 1946 to March 03, 2022
Search Strategy:

| # | Searches | Results |
|---|---|---|
| 1 | Colorectal Neoplasms/ | 100088 |
| 2 | Rectal Neoplasms/ | 45391 |
| 3 | ((colorectal or rectal or rectum) adj3 (cancer* or carcinoma* or neoplas* or tumo?r* or malignan*)).ti,ab,kw. | 170761 |
| 4 | Colonic polyps/ | 9269 |
| 5 | adenoma/ and exp intestine, large/ | 1953 |
| 6 | ((colorectal or rectal or rectum) adj3 (polyp* or adenoma*)).ti,ab,kw. | 11508 |
| 7 | (“colorectal polyp” or “serrated polyp”).ti,ab,kw. | 883 |
| 8 | or/1-7 | 208844 |
| 9 | (“clinical feature” or symptom*).ti,ab,kw. | 1296398 |
| 10 | (((bowel* or 3ntestine*) adj3 obstruction*) or (“abdominal pain” or fatigue or an?emia or constipation or diarrh?ea or “weight loss” or “gastrointestinal h?emorrhage” or h?ematochezia or “rectal bleed*”)).ti,ab,kw. | 547989 |
| 11 | Gastrointestinal Hemorrhage/ | 43832 |
| 12 | Intestinal Obstruction/ | 30776 |
| 13 | Abdominal Pain/ | 22748 |
| 14 | Anemia/ | 53062 |
| 15 | Fatigue/ | 32076 |
| 16 | Constipation/ | 15115 |
| 17 | Diarrhea/ | 50480 |
| 18 | Weight Loss/ | 40477 |
| 19 | or/9-18 | 1850715 |
| 20 | Validat$.mp. or Predict$.ti. or Rule$.mp. or (Predict$ and (Outcome$ or Risk$ or Model$)).mp. or ((History or Variable$ or Criteria or Scor$ or Characteristic$ or Finding$ or Factor$) and (Predict$ or Model$ or Decision$ or Identif$ or Prognos$)).mp. or (Decision$.mp. and ((Model$ or Clinical$).mp. or Logistic Models/)) or (Prognostic and (History or Variable$ or Criteria or Scor$ or Characteristic$ or Finding$ or Factor$ or Model$)).mp. [mp=title, abstract, original title, name of substance word, subject heading word, floating sub-heading word, keyword heading word, organism supplementary concept word, protocol supplementary concept word, rare disease supplementary concept word, unique identifier, synonyms] | 5532466 |
| 21 | exp “Sensitivity and Specificity”/ | 631973 |

| | | |
|---|---|---|
| 22 | sensitivity.tw. | 891726 |
| 23 | specificity.tw. | 510983 |
| 24 | ((pre-test or pretest) adj probability).tw. | 2585 |
| 25 | post-test probability.tw. | 673 |
| 26 | predictive value$.tw. | 123033 |
| 27 | likelihood ratio$.tw. | 17752 |
| 28 | or/21-27 | 1617403 |
| 29 | 20 or 28 | 6502020 |
| 30 | 8 and 19 and 29 | 5227 |

## EMBASE

Database(s): **Embase** 1974 to 2022 March 03
Search Strategy:

| # | Searches | Results |
|---|---|---|
| 1 | colorectal tumor/ | 26738 |
| 2 | rectum tumor/ | 14232 |
| 3 | ((colorectal or rectal or rectum) adj3 (cancer* or carcinoma* or neoplas* or tumo?r* or malignan*)).ti,ab,kw. | 253970 |
| 4 | colon polyp/ | 13698 |
| 5 | adenoma/ and exp large intestine/ | 3648 |
| 6 | ((colorectal or rectal or rectum) adj3 (polyp* or adenoma*)).ti,ab,kw. | 17622 |
| 7 | ("colorectal polyp" or "serrated polyp").ti,ab,kw. | 1816 |
| 8 | or/1-7 | 279404 |
| 9 | ("clinical feature" or symptom*).ti,ab,kw. | 1914307 |
| 10 | (((bowel* or intestin*) adj3 obstruction*) or ("abdominal pain" or fatigue or an?emia or constipation or diarrh?ea or "weight loss" or "gastrointestinal h?emorrhage" or h?ematochezia or "rectal bleed*")).ti,ab,kw. | 823550 |
| 11 | gastrointestinal hemorrhage/ | 66866 |
| 12 | intestine obstruction/ | 30920 |
| 13 | abdominal pain/ | 181408 |
| 14 | anemia/ | 204147 |
| 15 | fatigue/ | 233010 |
| 16 | constipation/ | 98854 |
| 17 | diarrhea/ | 257700 |
| 18 | body weight loss/ | 61152 |
| 19 | or/9-18 | 2921215 |
| 20 | Validat$.mp. or Predict$.ti. or Rule$.mp. or (Predict$ and (Outcome$ or Risk$ or Model$)).mp. or ((History or Variable$ or Criteria or Scor$ or Characteristic$ or Finding$ or Factor$) and (Predict$ or Model$ or Decision$ or Identif$ or Prognos$)).mp. or (Decision$.mp. and ((Model$ or Clinical$).mp. or Logistic Models/)) or (Prognostic and (History or Variable$ or Criteria or Scor$ or Characteristic$ or Finding$ or Factor$ or Model$)).mp. [mp=title, abstract, heading word, drug trade name, original title, device manufacturer, drug manufacturer, device trade name, keyword heading word, floating subheading word, candidate term word] | 7476614 |
| 21 | exp "SENSITIVITY AND SPECIFICITY"/ | 422875 |
| 22 | sensitivity.tw. | 1153136 |

| | | |
|---|---|---|
| 23 | specificity.tw. | 660171 |
| 24 | ((pre-test or pretest) adj probability).tw. | 4578 |
| 25 | post-test probability.tw. | 958 |
| 26 | predictive value$.tw. | 184292 |
| 27 | likelihood ratio$.tw. | 24160 |
| 28 | *Diagnostic Accuracy/ | 15771 |
| 29 | or/21-28 | 1711747 |
| 30 | 20 or 29 | 8496784 |
| 31 | 8 and 19 and 30 | 11222 |

## CINAHL

Friday, March 04, 2022 9:41:10 AM

| # | Query | Results |
|---|---|---|
| S30 | S8 AND S19 AND S29 | 936 |
| S29 | S20 OR S28 | 960,588 |
| S28 | S21 OR S22 OR S23 OR S24 OR S25 OR S26 OR S27 | 212,668 |
| S27 | TI likelihood ratio$ OR AB likelihood ratio$ | 6,242 |
| S26 | TI predictive value$ OR AB predictive value$ | 31,489 |
| S25 | TI post-test probability OR AB post-test probability | 290 |
| S24 | TI ((pre-test or pretest) N probability) OR AB ((pre-test or pretest) N probability) | 7 |
| S23 | TI Specificity OR AB Specificity | 67,370 |
| S22 | TI Sensitivity OR AB Sensitivity | 131,726 |
| S21 | (MH "Sensitivity and Specificity+") | 89,540 |
| S20 | TX (Validat$ or Predict$ or Rule$ or (Predict$ and (Outcome$ or Risk$ or Model$)) or ((History or Variable$ or Criteria or Scor$ or Characteristic$ or Finding$ or Factor$) and (Predict$ or Model$ or Decision$ or Identif$ or Prognos$)) or (Decision$ and ((Model$ or Clinical$) or Logistic Models/)) or (Prognostic and (History or Variable$ or Criteria or Scor$ or Characteristic$ or Finding$ or Factor$ or Model$))) | 806,127 |
| S19 | S9 OR S10 OR S11 OR S12 OR S13 OR S14 OR S15 OR S16 OR S17 OR S18 | 492,247 |
| S18 | (MH "Weight Loss") | 24,084 |
| S17 | (MH "Diarrhea") | 11,404 |
| S16 | (MH "Constipation") | 6,672 |
| S15 | (MH "Fatigue") | 20,073 |
| S14 | (MH "Anemia") | 11,014 |
| S13 | (MH "Abdominal Pain") | 9,881 |
| S12 | (MH "Intestinal Obstruction") | 4,347 |
| S11 | (MH "Gastrointestinal Hemorrhage") | 6,968 |
| S10 | TI (((bowel* or intestin*) N3 obstruction*) or ("abdominal pain" or fatigue or an#emia or constipation or diarrh#ea or "weight loss" or "gastrointestinal h#emorrhage" or h?ematochezia or "rectal bleed*")) OR AB (((bowel* or intestin*) N3 obstruction*) or ("abdominal pain" or fatigue or an?emia or constipation or diarrh#ea or "weight loss" or "gastrointestinal h#emorrhage" or h?ematochezia or "rectal bleed*")) | 121,849 |
| S9 | TI ("clinical feature" or symptom*) OR AB ("clinical feature" or symptom*) | 358,503 |
| S8 | S1 OR S2 OR S3 OR S4 OR S5 OR S6 OR S7 | 48,970 |
| S7 | TI ("colorectal polyp" or "serrated polyp") OR AB ("colorectal polyp" or "serrated polyp") | 239 |
| S6 | TI (((colorectal or rectal or rectum) N3 polyp*) or adenoma*) OR AB (((colorectal or rectal or rectum) N3 (polyp* or adenoma*) | 5,706 |
| S5 | (MH "Adenoma") AND (MH "Intestine, Large+") | 246 |
| S4 | (MH "Colonic Polyps") | 2,201 |
| S3 | TI ((colorectal or rectal or rectum) N3 (cancer* or carcinoma* or neoplas* or tumo#r* or malignan*)) OR AB ((colorectal or rectal or rectum) N3 (cancer* or carcinoma* or neoplas* or tumo#r* or malignan*)) | 35,837 |
| S2 | (MH "Rectal Neoplasms") | 5,584 |
| S1 | (MH "Colorectal Neoplasms") | 27,925 |

## Cochrane Libraries

| ID | Search | Hits |
|---|---|---|
| #1 | MeSH descriptor: [Colorectal Neoplasms] this term only | 5760 |
| #2 | MeSH descriptor: [Rectal Neoplasms] this term only | 1854 |
| #3 | ((colorectal or rectal or rectum) near/3 (cancer* or carcinoma* or neoplas* or tumo*r* or malignan*)):ti,ab,kw | 20496 |
| #4 | MeSH descriptor: [Colonic Polyps] this term only | 546 |
| #5 | MeSH descriptor: [Adenoma] this term only | 1079 |
| #6 | MeSH descriptor: [Intestine, Large] explode all trees | 3378 |
| #7 | (colorectal or rectal or rectum) near/3 (polyp* or adenoma*):ti,ab,kw | 1701 |
| #8 | ("colorectal polyp" or "serrated polyp"):ti,ab,kw | 406 |
| #9 | #5 AND #6 | 107 |
| #10 | #1 OR #2 OR #3 OR #4 OR #7 OR #8 OR #9 | 21261 |
| #11 | (((bowel* or intestin*) NEAR/3 obstruction*) or ("abdominal pain" or fatigue or an?emia or constipation or diarrh?ea or "weight loss" or "gastrointestinal h?emorrhage" or h?ematochezia or "rectal bleed*")):ti,ab,kw | 116141 |
| #12 | MeSH descriptor: [Gastrointestinal Hemorrhage] this term only | 1587 |
| #13 | MeSH descriptor: [Intestinal Obstruction] this term only | 361 |
| #14 | MeSH descriptor: [Abdominal Pain] this term only | 1141 |
| #15 | MeSH descriptor: [Anemia] this term only | 2562 |
| #16 | MeSH descriptor: [Fatigue] this term only | 3973 |
| #17 | MeSH descriptor: [Constipation] this term only | 1862 |
| #18 | MeSH descriptor: [Diarrhea] this term only | 3297 |
| #19 | MeSH descriptor: [Weight Loss] this term only | 6696 |
| #20 | {OR #11-#19} | 117609 |
| #21 | (Validat* or Predict* or Rule* or (Predict* and (Outcome* or Risk* or Model*)) or ((History or Variable* or Criteria or Scor* or Characteristic* or Finding* or Factor*) and (Predict* or Model* or Decision* or Identif* or Prognos*))or (Decision* and ((Model* or Clinical*) or Logistic Models)) or (Prognostic and (History or Variable* or Criteria or Scor* or Characteristic* or Finding* or Factor* or Model*))) | 322051 |
| #22 | MeSH descriptor: [Sensitivity and Specificity] explode all trees | 16114 |
| #23 | ("sensitivity"):ti,ab,kw | 63180 |
| #24 | ("specificity"):ti,ab,kw | 21470 |
| #25 | ((((pre-test or protest) NEAR probability))):ti,ab,kw | 87 |
| #26 | (post-test probability):ti,ab,kw | 115 |
| #27 | (predictive value*):ti,ab,kw | 17507 |
| #28 | (likelihood ratio*):ti,ab,kw | 3168 |
| #29 | {OR #22-#28} | 82294 |
| #30 | #21 OR #29 | 365055 |
| #31 | #10 AND #20 AND #30 | 673 |

## Scopus

( TITLE-ABS-KEY ( ( ( colorectal OR rectal OR rectum ) W/3 ( cancer* OR carcinoma* OR neoplas* OR tumour* OR tumor* OR malignan* OR polyp OR adenoma ) ) OR "colorectal polyp" OR "serrated polyp" ) ) AND ( TITLE-ABS-KEY ( "clinical feature" OR symptom* OR ( ( bowel* OR intestin* ) W/3 obstruction* ) OR "abdominal pain" OR fatigue OR an*emia OR constipation OR diarrh*ea OR "weight loss" OR "gastrointestinal h*emorrhage" OR h*ematochezia OR "rectal bleed*" ) ) AND ( TITLE-ABS-KEY ( ( validat* OR predict* OR rule* ) OR ( predict* AND ( outcome* OR risk* OR model* ) ) OR ( ( history OR variable* OR criteria OR scor* OR characteristic* OR finding* OR factor* ) AND ( predict* OR model* OR decision* OR identif* OR prognos* ) ) OR ( decisions AND ( model* OR clinical* ) ) OR ( logistic AND models ) OR ( prognostic AND ( history OR variable* OR criteria OR scor* OR characteristic* OR finding* OR factor* OR model* ) ) ) ) OR ( TITLE-ABS-KEY ( sensitivity OR specificity OR ( ( pre-test OR pretest ) W/1 probability ) OR ( post-test AND ( probability OR predictive AND value* OR likelihood AND ratio* ) ) ) ) ) AND ( not INDEX ( medline )

Supplementary file 3: Variables included in each paper and model

| | Stool tests+B1 | | | | | | Demographics | | | | Blood tests | | | | | | | | | | |
|---|---|---|---|---|---|---|---|---|---|---|---|---|---|---|---|---|---|---|---|---|---|
| **Author (year)** | **FIT** | **gFOBT** | **Faecal calprotectin** | **Bacterial markers** | **Faecal transfer** | **CRC markers** | **Age** | **Sex** | **BMI** | **Ethnicity** | **Hb** | **Plts** | **MCV** | **MCH** | **WBC** | **Ferritin** | **Protein biomarkers** | **Inflammatory markers** | **CEA** | **AFP** | **Ca19-9** |
| **Ayling, R (2021)** | | | | | | | | | | | | | | | | | | | | | |
| FAST | X | | | | | | X | X | | | | | | | | | | | | | |
| COLONFLAG AI (includes FBC but does not specify which elements; combines wit | X | | | | | | X | X | | | | | | | | | | | | | |
| **Cubiella, J (2017) FAST score** | X | | | | | | X | X | | | | | | | | | | | | | |
| **Cubiella, J 2016 (COLONPREDICT score)** | X | | | | | | X | X | | | | | | | | | | | X | | |
| **Digby, J (2019) (FAST score)** | X | | | | | | X | X | | | | | | | | | | | | | |
| **Fernandez-Banares, F. (2019) COLONOFIT** | X | | | | | | X | | | | | | | | | | | | | | |
| **Herrero, J M (2018) COLONPREDICT** | X | | | | | | X | X | | | | | | | | | | | X | | |
| **Hogberg, C (2020)** | | | | | | | | | | | | | | | | | | | | | |
| FIT & Hb | X | | | | | | | | | | X | | | | | | | | | | |
| FIT, Hb & Plts | X | | | | | | | | | | X | X | | | | | | | | | |
| **Hogberg, C (2017)** | | | | | | | | | | | | | | | | | | | | | |
| FIT and/or Faecal calpro. | X | | X | | | | | | | | | | | | | | | | | | |
| FIT and/or Hb | X | | | | | | | | | | X | | | | | | | | | | |
| FIT and/or and/Hb or Iron deficiency | X | | | | | | | | | | X | | | | | X | | | | | |
| **Jin, P (2012)** | X | | | | X | | | | | | | | | | | | | | | | |
| **Johansen, J S (2015) Review population could be exclude** | | | | | | | | | | | | | | | | | | | | | |
| 1) Combination of 2 tests CEA, Serum YKL-40 | | | | | | | | | | | | | | | | | YKL-40 | | X | | |
| 2) Model **(excludes patients with comorbidity)** | | | | | | | X | X | | | | | | | | | YKL-40 | | X | | |
| **Johnstone, M S (2022)** | X | | | | | | | | | | X | | | | | | | | | | |
| **Lue A (2020)** | X | | X | | | | | | | | | | | | | | | | | | |
| **Mahadavan, L (2012)** | | X | | | | | X | X | | | | | X | | | | | | X | | |
| **Malagon, M (2021)** | X | | | PTST, EUB, BCTF, BCTT | | | | | | | | | | | | | | | | | |
| **Mowat, C (2016)** | X | | X | | | | | | | | | | | | | | | | | | |
| **Parente F, (2012)** | | | | | | | | | | | | | | | | | | | | | |
| FIT & Faecal calprotectin (at least 1 positive) | X | | X | | | | | | | | | | | | | | | | | | |
| FIT, Faecal calprotectin & M2-PK (at least 1 positive) | X | | X | | M2-PK | | | | | | | | | | | | | | | | |
| FIT & M2-PK (at least 1 positive) | X | | | | M2-PK | | | | | | | | | | | | | | | | |
| Faecal calprotectin & M2-PK (at least 1 positive) | | | X | | M2-PK | | | | | | | | | | | | | | | | |
| **Rodriguez-Alonso, L (2015)** | X | | | | | | X | X | | | | | | | | | | | | | |
| **Turvill, J (2018)** | X | | X | | | | | | | | | | | | | | | | | | |
| **Widlak, M (2018)** | | | | | | | | | | | | | | | | | | | | | |
| FIT and Faecal calprotectin | X | | X | | | | | | | | | | | | | | | | | | |
| FIT and VOC | X | | | | | | | | | | | | | | | | | | | | |
| **Widlak, M (2017)** | X | | X | | | | | | | | | | | | | | | | | | |
| **Withrow (2022)** | | | | | | | | | | | | | | | | | | | | | |
| Model a | X | | | | | | X | X | | | | X | | X | | X | | X | | | |
| Model b | X | | | | | | | X | | | | | X | | | | | | | | |
| Model c | X | | | | | | | X | | | | | X | | | | | | | | |

| | PMHx | | | | | | | | Family Hx | Medications | | Lifestyle | | | | | |
|---|---|---|---|---|---|---|---|---|---|---|---|---|---|---|---|---|---|
| **Author (year)** | **Previous colonoscopy** | **Colonic polyps** | **Diverticular disease** | **Hypertension /BP** | **Relevant history (undefined)** | **Diabetes** | **Estrogen use** | **IBS** | **Gastrointestinal cancer** | **Aspirin use** | **NSAID use** | **Smoking Hx** | **Alcohol Hx** | **Red meat intake** | **Multivitamin use** | **Physical activity** | **Education level** |
| **Ayling, R (2021)** | | | | | | | | | | | | | | | | | |
| FAST | | | | | | | | | | | | | | | | | |
| COLONFLAG AI (includes FBC but does not specify which elements; combines wit | | | | | | | | | | | | | | | | | |
| **Cubiella, J (2017) FAST score** | | | | | | | | | | | | | | | | | |
| **Cubiella, J 2016 (COLONPREDICT score)** | X | | | | | | | | | X | | | | | | | |
| **Digby, J (2019) (FAST score)** | | | | | | | | | | | | | | | | | |
| **Fernandez-Banares, F. (2019) COLONOFIT** | X | | | | | | | | | | | X | | | | | |
| **Herrero, J M (2018) COLONPREDICT** | X | | | | | | | | | X | | | | | | | |
| **Hogberg, C (2020)** | | | | | | | | | | | | | | | | | |
| FIT & Hb | | | | | | | | | | | | | | | | | |
| FIT, Hb & Plts | | | | | | | | | | | | | | | | | |
| **Hogberg, C (2017)** | | | | | | | | | | | | | | | | | |
| FIT and/or Faecal calpro. | | | | | | | | | | | | | | | | | |
| FIT and/or Hb | | | | | | | | | | | | | | | | | |
| FIT and/or and/Hb or Iron deficiency | | | | | | | | | | | | | | | | | |
| **Jin, P (2012)** | | | | | | | | | | | | | | | | | |
| **Johansen, J S (2015) Review population could be exclude** | | | | | | | | | | | | | | | | | |
| 1) Combination of 2 tests CEA, Serum YKL-40 | | | | | | | | | | | | | | | | | |
| 2) Model **(excludes patients with comorbidity)** | | | | | | | | | | | | | | | | | |
| **Johnstone, M S (2022)** | | | | | | | | | | | | | | | | | |
| **Lue A (2020)** | | | | | | | | | | | | | | | | | |
| **Mahadavan, L (2012)** | | | | | | | | | | | | | | | | | |
| **Malagon, M (2021)** | | | | | | | | | | | | | | | | | |
| **Mowat, C (2016)** | | | | | | | | | | | | | | | | | |
| **Parente F, (2012)** | | | | | | | | | | | | | | | | | |
| FIT & Faecal calprotectin (at least 1 positive) | | | | | | | | | | | | | | | | | |
| FIT, Faecal calprotectin & M2-PK (at least 1 positive) | | | | | | | | | | | | | | | | | |
| FIT & M2-PK (at least 1 positive) | | | | | | | | | | | | | | | | | |
| Faecal calprotectin & M2-PK (at least 1 positive) | | | | | | | | | | | | | | | | | |
| **Rodriguez-Alonso, L (2015)** | | | | | | | | | | | | | | | | | |
| **Turvill, J (2018)** | | | | | | | | | | | | | | | | | |
| **Widlak, M (2018)** | | | | | | | | | | | | | | | | | |
| FIT and Faecal calprotectin | | | | | | | | | | | | | | | | | |
| FIT and VOC | | | | | | | | | | | | | | | | | |
| **Widlak, M (2017)** | | | | | | | | | | | | | | | | | |
| **Withrow (2022)** | | | | | | | | | | | | | | | | | |
| Model a | | | | | | | | | | | | | | | | | |
| Model b | | | | | | | | | | | | | | | | | |
| Model c | | | | | | | | | | | | | | | | | |

| | Symptoms/Signs | | | | | | | | | | | | | | | | | | | | | | | Other | | | |
|---|---|---|---|---|---|---|---|---|---|---|---|---|---|---|---|---|---|---|---|---|---|---|---|---|---|---|---|
| **Author (year)** | **Abdominal pain** | **Rectal bleeding** | **Change in bowel habit** | **Rectal mucous** | **Weight loss** | **Diarrhoea** | **Constipation** | **Peri-anal symptoms** | **Abdominal tenderness** | **Abdominal mass** | **Rectal mass** | **Abdominal symptoms (undefined)** | **Abnormal DRE** | **Benign anorectal lesion** | **Nausea or vomiting** | **Loss of appetite** | **Blood/Jelly stools** | **Harder stools** | **Anal pain** | **Fatigu e** | **Tenesmus/ emptying** | **Abnormal liver function** | **Anal swellin** | **Colonocyte DNA** | **VOC (urine)** | **DN A** | **Leucocyte adherance inhibition** |
| **Ayling, R (2021)** | | | | | | | | | | | | | | | | | | | | | | | | | | | |
| FAST | | | | | | | | | | | | | | | | | | | | | | | | | | | |
| COLONFLAG AI (includes FBC but does not specify which elements; combines wit | | | | | | | | | | | | | | | | | | | | | | | | | | | |
| **Cubiella, J (2017) FAST score** | | | | | | | | | | | | | | | | | | | | | | | | | | | |
| **Cubiella, J 2016 (COLONPREDICT score)** | | X | X | | | | | | | | | | | | | | | | | | | | | | | | |
| **Digby, J (2019) (FAST score)** | | | | | | | | | | | | | | | | | | | | | | | | | | | |
| **Fernandez-Banares, F. (2019) COLONOFIT** | | | | | | | | | | | | | | | | | | | | | | | | | | | |
| **Herrero, J M (2018) COLONPREDICT** | | X | X | | | | | | | | | | | | | | | | | | | | | | | | |
| **Hogberg, C (2020)** | | | | | | | | | | | | | | | | | | | | | | | | | | | |
| FIT & Hb | | | | | | | | | | | | | | | | | | | | | | | | | | | |
| FIT, Hb & Plts | | | | | | | | | | | | | | | | | | | | | | | | | | | |
| **Hogberg, C (2017)** | | | | | | | | | | | | | | | | | | | | | | | | | | | |
| FIT and/or Faecal calpro. | | | | | | | | | | | | | | | | | | | | | | | | | | | |
| FIT and/or Hb | | | | | | | | | | | | | | | | | | | | | | | | | | | |
| FIT and/or and/Hb or Iron deficiency | | | | | | | | | | | | | | | | | | | | | | | | | | | |
| **Jin, P (2012)** | | | | | | | | | | | | | | | | | | | | | | | | | | | |
| **Johansen, J S (2015) Review population could be exclude** | | | | | | | | | | | | | | | | | | | | | | | | | | | |
| 1) Combination of 2 tests CEA, Serum YKL-40 | | | | | | | | | | | | | | | | | | | | | | | | | | | |
| 2) Model **(excludes patients with comorbidity)** | | | | | | | | | | | | | | | | | | | | | | | | | | | |
| **Johnstone, M S (2022)** | | | | | | | | | | | | | | | | | | | | | | | | | | | |
| **Lue A (2020)** | | | | | | | | | | | | | | | | | | | | | | | | | | | |
| **Mahadavan, L (2012)** | | X | | | | | | | | | | | | | | | | | | | | | | X | | | |
| **Malagon, M (2021)** | | | | | | | | | | | | | | | | | | | | | | | | | | | |
| **Mowat, C (2016)** | | | | | | | | | | | | | | | | | | | | | | | | | | | |
| **Parente F, (2012)** | | | | | | | | | | | | | | | | | | | | | | | | | | | |
| FIT & Faecal calprotectin (at least 1 positive) | | | | | | | | | | | | | | | | | | | | | | | | | | | |
| FIT, Faecal calprotectin & M2-PK (at least 1 positive) | | | | | | | | | | | | | | | | | | | | | | | | | | | |
| FIT & M2-PK (at least 1 positive) | | | | | | | | | | | | | | | | | | | | | | | | | | | |
| Faecal calprotectin & M2-PK (at least 1 positive) | | | | | | | | | | | | | | | | | | | | | | | | | | | |
| **Rodriguez-Alonso, L (2015)** | | | | | | | | | | | | | | | | | | | | | | | | | | | |
| **Turvill, J (2018)** | | | | | | | | | | | | | | | | | | | | | | | | | | | |
| **Widlak, M (2018)** | | | | | | | | | | | | | | | | | | | | | | | | | | | |
| FIT and Faecal calprotectin | | | | | | | | | | | | | | | | | | | | | | | | | | | |
| FIT and VOC | | | | | | | | | | | | | | | | | | | | | | | | | X | | |
| **Widlak, M (2017)** | | | | | | | | | | | | | | | | | | | | | | | | | | | |
| **Withrow (2022)** | | | | | | | | | | | | | | | | | | | | | | | | | | | |
| Model a | | | | | | | | | | | | | | | | | | | | | | | | | | | |
| Model b | | | | | | | | | | | | | | | | | | | | | | | | | | | |
| Model c | | | | | | | | | | | | | | | | | | | | | | | | | | | |

Abbreviations:
FIT: Faecal immunochemical test
gFOBT: guaiac faecal occult blood testing
CRC: colorectal cancer
BMI: body mass index
Hb: Haemoglobin
MCV: Mean corpuscular volume
MCH: Mean corpuscular haemoglobin
WBC: white blood cell
CEA: Carcinoembryonic Antigen
BP: blood pressure
IBS: irrital bowel syndrome
NSAID: Non-steroidal anti-inflammatory drugs
Hx: history
DRE: digital rectal examination
VOC: volatile organic compound

| | Demographics | | | | Blood tests | | | | | | | | | | |
|---|---|---|---|---|---|---|---|---|---|---|---|---|---|---|---|
| **Author (year)** | **Age** | **Sex** | **BMI** | **Ethnicity** | **Hb** | **Plts** | **MCV** | **MCH** | **WBC** | **Ferritin** | **Protein biomarkers** | **Inflammatory markers e.g. CRP** | **CEA** | **AFP** | **Ca19-9** |
| **Abdelhady, S (2021)** | | | | | | | | | | | Golgi Protein 73 | | X | | |
| **Adelstein, B. A (2010)** | X | X | | | X | | | | | | | | | | |
| **Adelstein, B. A (2011)** | X | X | | | X | | | | | | | | | | |
| **Alatise, O. (2018)** | | | | | | | | | | | | | | | |
| **Ballal (2010)** | X | X | | | | | | | | | | | | | |
| **Blume, J E (2016)** | | | | | | | | | | | | | | | |
| CRC model 1 | | | | | | | | | | | AACT, CATD, CO3, CO9, MIF, PSGL, SEPR | | X | | |
| CRC model 2 | X | X | | | | | | | | | A1AG1, A1AT, CATD, CO9, OSTP, SEPR | | X | | |
| Advanced adenoma model | | | | | | | | | | | CATD, CLUS GDF15, SAA1 | | | | |
| **Collins G S (2012)** | | | | | | | | | | | | | | | |
| QCancer (Men) | X | | | | X | | | | | | | | | | |
| QCancer (Women) | X | | | | X | | | | | | | | | | |
| **Croner, L (2017)** | X | X | | | | | | | | | A1AG, CO9, DPPIV, MIF, PKM2, SAA, TFRC | | X | | |
| **Ellis, B (2005)** | | | | | | | | | | | | | | | |
| Rectal bleeding and CIBH | | | | | | | | | | | | | | | |
| Rectal bleeding and CIBH (loose) | | | | | | | | | | | | | | | |
| Rectal bleeding and CIBH (hard) | | | | | | | | | | | | | | | |
| Rectal bleeding and peri-anal symptoms | | | | | | | | | | | | | | | |
| Rectal bleeding, CIBH and abdominal pain | | | | | | | | | | | | | | | |
| **Ewing, M (2016)** | | | | | | | | | | | | | | | |
| CIBH & bleeding | | | | | | | | | | | | | | | |
| CIBH & Abdominal pain | | | | | | | | | | | | | | | |
| CIBH & Anaemia | | | | | X | | | | | | | | | | |
| Bleeding & Abdominal pain | | | | | | | | | | | | | | | |
| Bleeding & Anaemia | | | | | X | | | | | | | | | | |
| Weight loss & Anaemia | | | | | X | | | | | | | | | | |
| Abdominal pain & Anaemia | | | | | X | | | | | | | | | | |
| **Fijten, G. H (1995)** | X | | | | | | | | | | | | | | |
| **Hamilton, W. (2005)** | | | | | | | | | | | | | | | |
| **Hippersley-Cox J (2012)** | X | | | | X | | | | | | | | | | |
| QCancer (Males) | X | | | | X | | | | | | | | | | |
| QCancer (Females) | X | | | | X | | | | | | | | | | |
| **Hoogendoorn (2016) model 6 (most accurate)** | | | | | | | | | | | | | | | |
| **Johansen, J S (2015)** | | | | | | | | | | | | | | | |
| CEA and Serum YKL-40 | | | | | | | | | | | YKL-40 | | X | | |
| Model excluding patients with comorbidity | X | X | | | | | | | | | YKL-40 | | X | | |
| **Koning, N R (2015)** | X | X | | | | | | | | | | | | | |
| **Kop, R (2015)** | | | | | | | | | | | | | | | |
| **Kop, R (2016)** | | | | | | | | | | | | | | | |
| **Law, C W (2014)** | | | | | | | | | | | | | | | |
| CRC model | X | X | | X | | | | | | | | | | | |
| Colonic neoplasia model | X | | | X | | | | | | | | | | | |
| **Liu C (2021)** | | | | | | | | | | | SEPT9, SDC2, and SFRP2 | | | | |
| **Marshall, T (2011)** | | | | | | | | | | | | | | | |
| Birmingham Bristol Equation | | | | | X | | X | | | | | | | | |
| CAPER score | | | | | X | | | | | | | | | | |
| 3 Interpretations of NICE guidance + | | | | | | | | | | | | | | | |
| **Norrelund & Norrelund (1996)** | X | | | | | | | | | | | | | | |
| **Payne J (1983)** | | | | | | | | | | | | | X | | |
| **Rai (2008)** | | | | | | | | | | | | | | | |
| **Rasmussen (2017)** | X | X | | | | | | | | | | | X | | |
| **Rasmussen, L (2021)** | | | | | | | | | | | | | | | |
| CRC model | | | | | | | | | | | ANGPT2, ARG1, ICOSLG, IL8, CSF-1, Gal-9 | | | | |
| CRC/HRA model | | | | | | | | | | | ICOSLG, IL8 | | | | |
| **Selvachandran, S N (2002)** | | | | | | | | | | | | | | | |
| Weighted numerical score + (SELVA score) | X | X | | | | | | | | | | | | | |
| Malignancy risk score from Patient Questionnaire + | | | | | | | | | | | | | | | |
| Mailignancy risk score from GP letter + | | | | | | | | | | | | | | | |
| **Simpkins S (2017)** | | | | | | | | | | | | | | | |
| Age > 40, weight loss and abdo pain | X | | | | | | | | | | | | | | |
| Age > 50 and rectal bleeding | X | | | | | | | | | | | | | | |
| Age > 60 and anaemia | X | | | | X | | | | | | | | | | |
| Age > 60 and change in bowel habit | X | | | | | | | | | | | | | | |
| Age < 50, rectal bleeding and abdominal pain | X | | | | | | | | | | | | | | |
| Age < 50, rectal bleeding and change in bowel habit | X | | | | | | | | | | | | | | |
| Age < 50, rectal bleeding and weight loss | X | | | | | | | | | | | | | | |
| Age < 50, rectal bleeding and anaemia | X | | | | X | | | | | | | | | | |
| Any of NICE criteria | | | | | | | | | | | | | | | |
| **Stapley S (2017)** | | | | | | | | | | | | | | | |
| Rectal bleeding & CIBH | | | | | | | | | | | | | | | |
| Rectal bleeding & Diarrhoea | | | | | | | | | | | | | | | |
| Rectal bleeding & Abdominal pain | | | | | | | | | | | | | | | |
| Rectal bleeding & MCV | | | | | | | X | | | | | | | | |
| Rectal bleeding & WBC | | | | | | | | | X | | | | | | |
| Rectal bleeding & platelets | | | | | | X | | | | | | | | | |
| Rectal bleeding & Abnormal liver function | | | | | | | | | | | | | | | |
| Rectal bleeding & Hb | | | | | X | | | | | | | | | | |
| Rectal bleeding & Inflammatory markers | | | | | | | | | | | | X | | | |
| CIBH & Diarrhoea | | | | | | | | | | | | | | | |
| CIBH & Abdominal pain | | | | | | | | | | | | | | | |
| CIBH & MCV | | | | | | | X | | | | | | | | |
| CBH & WBC | | | | | | | | | X | | | | | | |
| CIBH & platelets | | | | | | X | | | | | | | | | |
| CIBH & Abnormal Liver function | | | | | | | | | | | | | | | |
| CIBH & Hb | | | | | X | | | | | | | | | | |
| CIBH & Inflammatory markers | | | | | | | | | | | | X | | | |
| Diarrhoea & Abdominal pain | | | | | | | | | | | | | | | |
| Diarrhoea & MCV | | | | | | | X | | | | | | | | |
| Diarrhoea & platelets | | | | | | X | | | | | | | | | |
| Diarrhoea & Abnormal liver function | | | | | | | | | | | | | | | |
| Diarrhoea & Hb | | | | | X | | | | | | | | | | |
| Diarrhoea & Inflammatory markers | | | | | | | | | | | | X | | | |
| Abdominal pain & MCV | | | | | | | X | | | | | | | | |
| Abdominal pain & WBC | | | | | | | | | X | | | | | | |
| Abdominal pain & platelets | | | | | | X | | | | | | | | | |
| Abdominal pain & Abnormal liver function | | | | | | | | | | | | | | | |
| Abdominal pain & Hb | | | | | X | | | | | | | | | | |
| Abdominal pain & Inflammatory markers | | | | | | | | | | | | X | | | |
| MCV & WBC | | | | | | | X | | X | | | | | | |
| MCV & Platelets | | | | | | X | X | | | | | | | | |
| MCV & Abnormal liver function | | | | | | | X | | | | | | | | |
| MCV & Hb | | | | | X | | X | | | | | | | | |
| MCV & Inflammatory markers | | | | | | | X | | | | | X | | | |
| WBC & Platelets | | | | | | X | | | X | | | | | | |
| WBC & Abnormal liver function | | | | | | | | | X | | | | | | |
| WBC & Hb | | | | | X | | | | X | | | | | | |
| WBC & Inflammatory markers | | | | | | | | | X | | | X | | | |
| Platelets & Abnormal liver function | | | | | | X | | | | | | | | | |
| Platelets & Hb | | | | | X | X | | | | | | | | | |
| Platelets & Inflammatory markers | | | | | | X | | | | | | X | | | |
| Abnormal liver function & Hb | | | | | X | | | | | | | | | | |
| Abnormal liver function & Inflammatory markers | | | | | | | | | | | | X | | | |
| Hb & Inflammatory markers | | | | | X | | | | | | | X | | | |
| **Steffen (2014)** | X | X | X | | | | | | | | | | | | |
| **Thompson, M (2017)** | X | X | | | X | | | | | X | | | | | |
| **Wells (2014)** | | | | | | | | | | | | | | | |
| Male | X | | X | X | | | | | | | | | | | |
| Female | X | | X | X | | | | | | | | | | | |
| **Whitfield, A (2018)** | X | | | | | X | | X | X | X | | | | | |
| **Wilhelmson, M (2018)** | | | | | | | | | | | | | | | |
| 2 models for CRC | | | | | | | | | | | | | | | |
| Model 1 | X | X | | | | | | | | X | Pepsinogen 2, HE4, CyFra21-1 | X (hs-CRP) | X | | |
| Model 2 | X | X | | | | | | | | X | HE4, CyFra21-1 | | X | | |
| **Wilhelmson, M (2017)** | | | | | | | | | | | | | | | |
| 2 models for CRC (AUROC for CRC and CRC/HRA) | | | | | | | | | | | | | | | |
| Model 1 - 4 biomarkers | X | X | | | | | | | | X | CyFra21-1 | X (hs-CRP) | X | | |
| Model 2 - 8 biomarkers | X | X | | | | | | | | X | CyFra21-1, Galectin-3, TIMP-1 | X (hs-CRP) | X | X | X |
| **Wilson, S (2012)** | X | X | | | | | | | X | | MMP-9 | | | | |

| | PMHx | | | | | | | | Family Hx | Medications | | Lifestyle | | | | | |
|---|---|---|---|---|---|---|---|---|---|---|---|---|---|---|---|---|---|
| **Author (year)** | **Previous colonoscopy** | **Colonic polyps** | **Diverticular disease** | **Hypertension/BP** | **Relevant history (undefined)** | **Diabetes** | **Estrogen use** | **IBS** | **Gastrointestinal cancer** | **Aspirin use** | **NSAID use** | **Smoking Hx** | **Alcohol Hx** | **Red meat intake** | **multivitamin use** | **Physical activity** | **Education level** |
| **Abdelhady, S (2021)** | | | | | | | | | | | | | | | | | |
| **Adelstein, B. A (2010)** | X | | X | | | | | | | X | X | | | | | | |
| **Adelstein, B. A (2011)** | X | X | | | | | | X | | X | X | X | | | | | X |
| **Alatise, O. (2018)** | | | | | | | | | | | | | | | | | |
| **Ballal (2010)** | | | | | X | | | | X | | | | | | | | |
| **Blume, J E (2016)** | | | | | | | | | | | | | | | | | |
| CRC model 1 | | | | | | | | | | | | | | | | | |
| CRC model 2 | | | | | | | | | | | | | | | | | |
| Advanced adenoma model | | | | | | | | | | | | | | | | | |
| **Collins G S (2012)** | | | | | | | | | | | | | | | | | |
| QCancer (Men) | | | | | | | | | X | | | | X | | | | |
| QCancer (Women) | | | | | | | | | X | | | | | | | | |
| **Croner, L (2017)** | | | | | | | | | | | | | | | | | |
| **Ellis, B (2005)** | | | | | | | | | | | | | | | | | |
| Rectal bleeding and CIBH | | | | | | | | | | | | | | | | | |
| Rectal bleeding and CIBH (loose) | | | | | | | | | | | | | | | | | |
| Rectal bleeding and CIBH (hard) | | | | | | | | | | | | | | | | | |
| Rectal bleeding and peri-anal symptoms | | | | | | | | | | | | | | | | | |
| Rectal bleeding, CIBH and abdominal pain | | | | | | | | | | | | | | | | | |
| **Ewing, M (2016)** | | | | | | | | | | | | | | | | | |
| CIBH & bleeding | | | | | | | | | | | | | | | | | |
| CIBH & Abdominal pain | | | | | | | | | | | | | | | | | |
| CIBH & Anaemia | | | | | | | | | | | | | | | | | |
| Bleeding & Abdominal pain | | | | | | | | | | | | | | | | | |
| Bleeding & Anaemia | | | | | | | | | | | | | | | | | |
| Weight loss & Anaemia | | | | | | | | | | | | | | | | | |
| Abdominal pain & Anaemia | | | | | | | | | | | | | | | | | |
| **Fijten, G. H (1995)** | | | | | | | | | | | | | | | | | |
| **Hamilton, W. (2005)** | | | | | | | | | | | | | | | | | |
| **Hippersley-Cox J (2012)** | | | | | | | | | X | | | | | | | | |
| QCancer (Males) | | | | | | | | | X | | | | X | | | | |
| QCancer (Females) | | | | | | | | | X | | | | | | | | |
| **Hoogendoorn (2016) model 6 (most accurate)** | | | | | | | | | | | | | | | | | |
| **Johansen, J S (2015)** | | | | | | | | | | | | | | | | | |
| CEA and Serum YKL-40 | | | | | | | | | | | | | | | | | |
| Model excluding patients with comorbidity | | | | | | | | | | | | | | | | | |
| **Koning, N R (2015)** | | | | X | | | | | | | | | | | | | |
| **Kop, R (2015)** | | | | | | | | | | | | | | | | | |
| **Kop, R (2016)** | | | | | | | | | | | | | | | | | |
| **Law, C W (2014)** | | | | | | | | | | | | | | | | | |
| CRC model | | | | | | | | | | X | | X | | | | | X |
| Colonic neoplasia model | | | | | | | | | | | | X | | | | | X |
| **Liu C (2021)** | | | | | | | | | | | | | | | | | |
| **Marshall, T (2011)** | | | | | | | | | | | | | | | | | |
| Birmingham Bristol Equation | | | | | | | | | | | | | | | | | |
| CAPER score | | | | | | | | | | | | | | | | | |
| 3 Interpretations of NICE guidance + | | | | | | | | | | | | | | | | | |
| **Norrelund & Norrelund (1996)** | | | | | | | | | | | | | | | | | |
| **Payne J (1983)** | | | | | | | | | | | | | | | | | |
| **Rai (2008)** | | | | | | | | | | | | | | | | | |
| **Rasmussen (2017)** | | | | | | | | | | | | | | | | | |
| **Rasmussen, L (2021)** | | | | | | | | | | | | | | | | | |
| CRC model | | | | | | | | | | | | | | | | | |
| CRC/HRA model | | | | | | | | | | | | | | | | | |
| **Selvachandran, S N (2002)** | | | | | | | | | | | | | | | | | |
| Weighted numerical score + (SELVA score) | | | | | X | | | | X | | | | | | | | |
| Malignancy risk score from Patient Questionnaire + | | | | | | | | | | | | | | | | | |
| Mailignancy risk score from GP letter + | | | | | | | | | | | | | | | | | |
| **Simpkins S (2017)** | | | | | | | | | | | | | | | | | |
| Age > 40, weight loss and abdo pain | | | | | | | | | | | | | | | | | |
| Age > 50 and rectal bleeding | | | | | | | | | | | | | | | | | |
| Age > 60 and anaemia | | | | | | | | | | | | | | | | | |
| Age > 60 and change in bowel habit | | | | | | | | | | | | | | | | | |
| Age < 50, rectal bleeding and abdominal pain | | | | | | | | | | | | | | | | | |
| Age < 50, rectal bleeding and change in bowel habit | | | | | | | | | | | | | | | | | |
| Age < 50, rectal bleeding and weight loss | | | | | | | | | | | | | | | | | |
| Age < 50, rectal bleeding and anaemia | | | | | | | | | | | | | | | | | |
| Any of NICE criteria | | | | | | | | | | | | | | | | | |
| **Stapley S (2017)** | | | | | | | | | | | | | | | | | |
| Rectal bleeding & CIBH | | | | | | | | | | | | | | | | | |
| Rectal bleeding & Diarrhoea | | | | | | | | | | | | | | | | | |
| Rectal bleeding & Abdominal pain | | | | | | | | | | | | | | | | | |
| Rectal bleeding & MCV | | | | | | | | | | | | | | | | | |
| Rectal bleeding & WBC | | | | | | | | | | | | | | | | | |
| Rectal bleeding & platelets | | | | | | | | | | | | | | | | | |
| Rectal bleeding & Abnormal liver function | | | | | | | | | | | | | | | | | |
| Rectal bleeding & Hb | | | | | | | | | | | | | | | | | |
| Rectal bleeding & Inflammatory markers | | | | | | | | | | | | | | | | | |
| CIBH & Diarrhoea | | | | | | | | | | | | | | | | | |
| CIBH & Abdominal pain | | | | | | | | | | | | | | | | | |
| CIBH & MCV | | | | | | | | | | | | | | | | | |
| CBH & WBC | | | | | | | | | | | | | | | | | |
| CIBH & platelets | | | | | | | | | | | | | | | | | |
| CIBH & Abnormal Liver function | | | | | | | | | | | | | | | | | |
| CIBH & Hb | | | | | | | | | | | | | | | | | |
| CIBH & Inflammatory markers | | | | | | | | | | | | | | | | | |
| Diarrhoea & Abdominal pain | | | | | | | | | | | | | | | | | |
| Diarrhoea & MCV | | | | | | | | | | | | | | | | | |
| Diarrhoea & platelets | | | | | | | | | | | | | | | | | |
| Diarrhoea & Abnormal liver function | | | | | | | | | | | | | | | | | |
| Diarrhoea & Hb | | | | | | | | | | | | | | | | | |
| Diarrhoea & Inflammatory markers | | | | | | | | | | | | | | | | | |
| Abdominal pain & MCV | | | | | | | | | | | | | | | | | |
| Abdominal pain & WBC | | | | | | | | | | | | | | | | | |
| Abdominal pain & platelets | | | | | | | | | | | | | | | | | |
| Abdominal pain & Abnormal liver function | | | | | | | | | | | | | | | | | |
| Abdominal pain & Hb | | | | | | | | | | | | | | | | | |
| Abdominal pain & Inflammatory markers | | | | | | | | | | | | | | | | | |
| MCV & WBC | | | | | | | | | | | | | | | | | |
| MCV & Platelets | | | | | | | | | | | | | | | | | |
| MCV & Abnormal liver function | | | | | | | | | | | | | | | | | |
| MCV & Hb | | | | | | | | | | | | | | | | | |
| MCV & Inflammatory markers | | | | | | | | | | | | | | | | | |
| WBC & Platelets | | | | | | | | | | | | | | | | | |
| WBC & Abnormal liver function | | | | | | | | | | | | | | | | | |
| WBC & Hb | | | | | | | | | | | | | | | | | |
| WBC & Inflammatory markers | | | | | | | | | | | | | | | | | |
| Platelets & Abnormal liver function | | | | | | | | | | | | | | | | | |
| Platelets & Hb | | | | | | | | | | | | | | | | | |
| Platelets & Inflammatory markers | | | | | | | | | | | | | | | | | |
| Abnormal liver function & Hb | | | | | | | | | | | | | | | | | |
| Abnormal liver function & Inflammatory markers | | | | | | | | | | | | | | | | | |
| Hb & Inflammatory markers | | | | | | | | | | | | | | | | | |
| **Steffen (2014)** | X | | | | | X | | | | | | X | X | | | | |
| **Thompson, M (2017)** | | | | | | | | | | | | | | | | | |
| **Wells (2014)** | | | | | | | | | | | | | | | | | |
| Male | | | | | | X | | | X | X | | X | X | X | X | X | X |
| Female | | | | | | X | X | | X | | X | X | X | | X | | X |
| **Whitfield, A (2018)** | | | | | | | | | | | | | | | | | |
| **Wilhelmson, M (2018)** | | | | | | | | | | | | | | | | | |
| 2 models for CRC | | | | | | | | | | | | | | | | | |
| Model 1 | | | | | | | | | | | | | | | | | |
| Model 2 | | | | | | | | | | | | | | | | | |
| **Wilhelmson, M (2017)** | | | | | | | | | | | | | | | | | |
| 2 models for CRC (AUROC for CRC and CRC/HRA) | | | | | | | | | | | | | | | | | |
| Model 1 - 4 biomarkers | | | | | | | | | | | | | | | | | |
| Model 2 - 8 biomarkers | | | | | | | | | | | | | | | | | |
| **Wilson, S (2012)** | | | | X | | | | | | | | X | X | | | | |

| | Symptoms/Signs | | | | | | | | | | | | | | | | | | | | | | | Other | | |
|---|---|---|---|---|---|---|---|---|---|---|---|---|---|---|---|---|---|---|---|---|---|---|---|---|---|---|
| **Author (year)** | **Abdominal pain** | **Rectal bleeding** | **Change in bowel habit** | **Rectal mucous** | **Weight loss** | **Diarrhoea** | **Constipation** | **Peri-anal symptoms** | **Abdominal tenderness** | **Abdominal mass** | **Rectal mass** | **Abdominal symptoms (undefined)** | **Abnormal DRE** | **Benign anorectal lesion** | **Nausea or vomiting** | **Loss of appetite** | **Blood/Jelly stools** | **Harder stools** | **Anal pain** | **Fatigue** | **Tenesmus/emptying** | **Abnormal liver function** | **Anal swelling** | **Colonocyte DNA** | **VOC (urine)** | **DNA** |
| **Abdelhady, S (2021)** | | | | | | | | | | | | | | | | | | | | | | | | | | |
| **Adelstein, B. A (2010)** | X | X | | X | | | | | | | | | | | | | | | | | | | | | | |
| **Adelstein, B. A (2011)** | | X | | X | | | | | | | | | | | | | | | | X | | | | | | |
| **Alatise, O. (2018)** | | | X | | X | | | | | | | | | | | | | | | | | | | | | |
| **Ballal (2010)** | | X | X | | X | | | X | | | | X | | | | X | | | | X | X | | | | | |
| **Blume, J E (2016)** | | | | | | | | | | | | | | | | | | | | | | | | | | |
| CRC model 1 | | | | | | | | | | | | | | | | | | | | | | | | | | |
| CRC model 2 | | | | | | | | | | | | | | | | | | | | | | | | | | |
| Advanced adenoma model | | | | | | | | | | | | | | | | | | | | | | | | | | |
| **Collins G S (2012)** | | | | | | | | | | | | | | | | | | | | | | | | | | |
| QCancer (Men) | X | X | X | | X | | | | | | | | | | | X | | | | | | | | | | |
| QCancer (Women) | X | X | | | X | | | | | | | | | | | X | | | | | | | | | | |
| **Croner, L (2017)** | | | | | | | | | | | | | | | | | | | | | | | | | | |
| **Ellis, B (2005)** | | | | | | | | | | | | | | | | | | | | | | | | | | |
| Rectal bleeding and CIBH | | X | X | | | | | | | | | | | | | | | | | | | | | | | |
| Rectal bleeding and CIBH (loose) | | X | | | | X | | | | | | | | | | | | | | | | | | | | |
| Rectal bleeding and CIBH (hard) | | X | | | | | | | | | | | | | | | | X | | | | | | | | |
| Rectal bleeding and peri-anal symptoms | | X | | | | | | X | | | | | | | | | | | | | | | | | | |
| Rectal bleeding, CIBH and abdominal pain | X | X | X | | | | | | | | | | | | | | | | | | | | | | | |
| **Ewing, M (2016)** | | | | | | | | | | | | | | | | | | | | | | | | | | |
| CIBH & bleeding | | X | X | | | | | | | | | | | | | | | | | | | | | | | |
| CIBH & Abdominal pain | X | | X | | | | | | | | | | | | | | | | | | | | | | | |
| CIBH & Anaemia | | | X | | | | | | | | | | | | | | | | | | | | | | | |
| Bleeding & Abdominal pain | X | X | | | | | | | | | | | | | | | | | | | | | | | | |
| Bleeding & Anaemia | | X | | | | | | | | | | | | | | | | | | | | | | | | |
| Weight loss & Anaemia | | | | | X | | | | | | | | | | | | | | | | | | | | | |
| Abdominal pain & Anaemia | X | | | | | | | | | | | | | | | | | | | | | | | | | |
| **Fijten, G. H (1995)** | | X | X | | | | | | | | | | | | | | | | | | | | | | | |
| **Hamilton, W. (2005)** | X | X | | | X | X | X | | x | | | | X | | | | | | | | | | | | | |
| **Hippersley-Cox J (2012)** | X | X | | | X | | | | | | | | | | | X | | | | | | | | | | |
| QCancer (Males) | X | X | X | | X | | | | | | | | | | | X | | | | | | | | | | |
| QCancer (Females) | X | X | | | X | | | | | | | | | | | X | | | | | | | | | | |
| **Hoogendoorn (2016) model 6 (most accurate)** | | | | | | | | | | | | | | | | | | | | | | | | | | |
| **Johansen, J S (2015)** | | | | | | | | | | | | | | | | | | | | | | | | | | |
| CEA and Serum YKL-40 | | | | | | | | | | | | | | | | | | | | | | | | | | |
| Model excluding patients with comorbidity | | | | | | | | | | | | | | | | | | | | | | | | | | |
| **Koning, N R (2015)** | X | | | | | | | | | | | | | | | | | | | | | | | | | |
| **Kop, R (2015)** | | | | | | | | | | | | | | | | | | | | | | | | | | |
| **Kop, R (2016)** | | | | | | | | | | | | | | | | | | | | | | | | | | |
| **Law, C W (2014)** | | | | | | | | | | | | | | | | | | | | | | | | | | |
| CRC model | X | | X | | X | | | | | | | | | | | X | X | | | | | | X | | | |
| Colonic neoplasia model | | | X | | X | | | | | | | | | | | X | X | | | | | | | | | |
| **Liu C (2021)** | | | | | | | | | | | | | | | | | | | | | | | | | | |
| **Marshall, T (2011)** | | | | | | | | | | | | | | | | | | | | | | | | | | |
| Birmingham Bristol Equation | X | X | X | | X | X | X | | | X | | | X | | | | | | | | | | | | | |
| CAPER score | X | X | | | X | X | X | | | X | | | X | | | | | | | | | | | | | |
| 3 Interpretations of NICE guidance + | | | | | | | | | | | | | | | | | | | | | | | | | | |
| **Norrelund & Norrelund (1996)** | | | X | | | | | | | | | | | | | | | | | | | | | | | |
| **Payne J (1983)** | | | | | | | | | | | | | | | | | | | | | | | | | | |
| **Rai (2008)** | | | | | | | | | | | | | | | | | | | | | | | | | | |
| **Rasmussen (2017)** | | | | | | | | | | | | | | | | | | | | | | | | | | ccfn containing 5-methylcytosine DNA (5 mC) |
| **Rasmussen, L (2021)** | | | | | | | | | | | | | | | | | | | | | | | | | | |
| CRC model | | | | | | | | | | | | | | | | | | | | | | | | | | |
| CRC/HRA model | | | | | | | | | | | | | | | | | | | | | | | | | | |
| **Selvachandran, S N (2002)** | | | | | | | | | | | | | | | | | | | | | | | | | | |
| Weighted numerical score + (SELVA score) | | X | X | | X | | | X | | | | X | | | | X | | | | X | X | | | | | |
| Malignancy risk score from Patient Questionnaire + | | | | | | | | | | | | | | | | | | | | | | | | | | |
| Mailignancy risk score from GP letter + | | | | | | | | | | | | | | | | | | | | | | | | | | |
| **Simpkins S (2017)** | | | | | | | | | | | | | | | | | | | | | | | | | | |
| Age > 40, weight loss and abdo pain | X | | | | X | | | | | | | | | | | | | | | | | | | | | |
| Age > 50 and rectal bleeding | | X | | | | | | | | | | | | | | | | | | | | | | | | |
| Age > 60 and anaemia | | | | | | | | | | | | | | | | | | | | | | | | | | |
| Age > 60 and change in bowel habit | | | X | | | | | | | | | | | | | | | | | | | | | | | |
| Age < 50, rectal bleeding and abdominal pain | X | X | | | | | | | | | | | | | | | | | | | | | | | | |
| Age < 50, rectal bleeding and change in bowel habit | | X | X | | | | | | | | | | | | | | | | | | | | | | | |
| Age < 50, rectal bleeding and weight loss | | X | | | X | | | | | | | | | | | | | | | | | | | | | |
| Age < 50, rectal bleeding and anaemia | | X | | | | | | | | | | | | | | | | | | | | | | | | |
| Any of NICE criteria | | | | | | | | | | | | | | | | | | | | | | | | | | |
| **Stapley S (2017)** | | | | | | | | | | | | | | | | | | | | | | | | | | |
| Rectal bleeding & CIBH | | X | X | | | | | | | | | | | | | | | | | | | | | | | |
| Rectal bleeding & Diarrhoea | | X | | | | X | | | | | | | | | | | | | | | | | | | | |
| Rectal bleeding & Abdominal pain | X | X | | | | | | | | | | | | | | | | | | | | | | | | |
| Rectal bleeding & MCV | | X | | | | | | | | | | | | | | | | | | | | | | | | |
| Rectal bleeding & WBC | | X | | | | | | | | | | | | | | | | | | | | | | | | |
| Rectal bleeding & platelets | | X | | | | | | | | | | | | | | | | | | | | | | | | |
| Rectal bleeding & Abnormal liver function | | X | | | | | | | | | | | | | | | | | | | | X | | | | |
| Rectal bleeding & Hb | | X | | | | | | | | | | | | | | | | | | | | | | | | |
| Rectal bleeding & Inflammatory markers | | X | | | | | | | | | | | | | | | | | | | | | | | | |
| CIBH & Diarrhoea | | | X | | | X | | | | | | | | | | | | | | | | | | | | |
| CIBH & Abdominal pain | X | | X | | | | | | | | | | | | | | | | | | | | | | | |
| CIBH & MCV | | | X | | | | | | | | | | | | | | | | | | | | | | | |
| CBH & WBC | | | X | | | | | | | | | | | | | | | | | | | | | | | |
| CIBH & platelets | | | X | | | | | | | | | | | | | | | | | | | | | | | |
| CIBH & Abnormal Liver function | | | X | | | | | | | | | | | | | | | | | | | X | | | | |
| CIBH & Hb | | | X | | | | | | | | | | | | | | | | | | | | | | | |
| CIBH & Inflammatory markers | | | X | | | | | | | | | | | | | | | | | | | | | | | |
| Diarrhoea & Abdominal pain | X | | | | | X | | | | | | | | | | | | | | | | | | | | |
| Diarrhoea & MCV | | | | | | X | | | | | | | | | | | | | | | | | | | | |
| Diarrhoea & platelets | | | | | | X | | | | | | | | | | | | | | | | | | | | |
| Diarrhoea & Abnormal liver function | | | | | | X | | | | | | | | | | | | | | | | X | | | | |
| Diarrhoea & Hb | | | | | | X | | | | | | | | | | | | | | | | | | | | |
| Diarrhoea & Inflammatory markers | | | | | | X | | | | | | | | | | | | | | | | | | | | |
| Abdominal pain & MCV | X | | | | | | | | | | | | | | | | | | | | | | | | | |
| Abdominal pain & WBC | X | | | | | | | | | | | | | | | | | | | | | | | | | |
| Abdominal pain & platelets | X | | | | | | | | | | | | | | | | | | | | | | | | | |
| Abdominal pain & Abnormal liver function | X | | | | | | | | | | | | | | | | | | | | | X | | | | |
| Abdominal pain & Hb | X | | | | | | | | | | | | | | | | | | | | | | | | | |
| Abdominal pain & Inflammatory markers | X | | | | | | | | | | | | | | | | | | | | | | | | | |
| MCV & WBC | | | | | | | | | | | | | | | | | | | | | | | | | | |
| MCV & Platelets | | | | | | | | | | | | | | | | | | | | | | | | | | |
| MCV & Abnormal liver function | | | | | | | | | | | | | | | | | | | | | | X | | | | |
| MCV & Hb | | | | | | | | | | | | | | | | | | | | | | | | | | |
| MCV & Inflammatory markers | | | | | | | | | | | | | | | | | | | | | | | | | | |
| WBC & Platelets | | | | | | | | | | | | | | | | | | | | | | | | | | |
| WBC & Abnormal liver function | | | | | | | | | | | | | | | | | | | | | | X | | | | |
| WBC & Hb | | | | | | | | | | | | | | | | | | | | | | | | | | |
| WBC & Inflammatory markers | | | | | | | | | | | | | | | | | | | | | | | | | | |
| Platelets & Abnormal liver function | | | | | | | | | | | | | | | | | | | | | | | | | | |
| Platelets & Hb | | | | | | | | | | | | | | | | | | | | | | | | | | |
| Platelets & Inflammatory markers | | | | | | | | | | | | | | | | | | | | | | | | | | |
| Abnormal liver function & Hb | | | | | | | | | | | | | | | | | | | | | | X | | | | |
| Abnormal liver function & Inflammatory markers | | | | | | | | | | | | | | | | | | | | | | X | | | | |
| Hb & Inflammatory markers | | | | | | | | | | | | | | | | | | | | | | | | | | |
| **Steffen (2014)** | | | | | | | | | | | | | | | | | | | | | | | | | | |
| **Thompson, M (2017)** | X | X | X | | X | | | X | | X | X | | | | | | | | | | | | | | | |
| **Wells (2014)** | | | | | | | | | | | | | | | | | | | | | | | | | | |
| Male | | | | | | | | | | | | | | | | | | | | | | | | | | |
| Female | | | | | | | | | | | | | | | | | | | | | | | | | | |
| **Whitfield, A (2018)** | | X | | | | | | | | | | | | | | | | | | | | | | | | |
| **Wilhelmson, M (2018)** | | | | | | | | | | | | | | | | | | | | | | | | | | |
| 2 models for CRC | | | | | | | | | | | | | | | | | | | | | | | | | | |
| Model 1 | | | | | | | | | | | | | | | | | | | | | | | | | | |
| Model 2 | | | | | | | | | | | | | | | | | | | | | | | | | | |
| **Wilhelmson, M (2017)** | | | | | | | | | | | | | | | | | | | | | | | | | | |
| 2 models for CRC (AUROC for CRC and CRC/HRA) | | | | | | | | | | | | | | | | | | | | | | | | | | |
| Model 1 - 4 biomarkers | | | | | | | | | | | | | | | | | | | | | | | | | | |
| Model 2 - 8 biomarkers | | | | | | | | | | | | | | | | | | | | | | | | | | |
| **Wilson, S (2012)** | | | | | X | | | | | | | | | | | | X | X | X | | | | | | | |

| Leucocyte adherance inhibition |
| --- |
| |
| |
| |
| |
| |
| |
| |
| |
| |
| |
| |
| |
| |
| |
| |
| |
| |
| |
| |
| |
| |
| |
| |
| |
| |
| |
| |
| |
| |
| |
| |
| |
| |
| |
| |
| |
| |
| |
| |
| |
| |
| |
| |
| |
| |
| |
| |
| |
| x |
| |
| |
| |
| |
| |
| |
| |
| |
| |
| |
| |
| |
| |
| |
| |
| |
| |
| |
| |
| |
| |
| |
| |
| |
| |
| |
| |
| |
| |
| |
| |
| |
| |
| |
| |
| |
| |
| |
| |
| |
| |
| |
| |
| |
| |
| |
| |
| |
| |
| |
| |
| |
| |
| |
| |
| |
| |
| |
| |
| |
| |
| |
| |
| |
| |
| |
| |
| |
| |
| |
| |

Supplementary file 4: Excluded studies at full text screening with reason

| Reference | Reason for exclusion |
|---|---|
| Kinar, Y.; Kalkstein, N.; Akiva, P.; Levin, B.; Half, E. E.; Goldshtein, I.; Chodick, G.; Shalev, V. 2016 Development and validation of a predictive model for detection of colorectal cancer in primary care by analysis of complete blood counts: a binational retrospective study. Journal of the American Medical Informatics Association 23:5 | Wrong population |
| Hamilton, W.; Green, T.; Martins, T.; Elliott, K.; Rubin, G.; Macleod, U. 2013 Evaluation of risk assessment tools for suspected cancer in general practice: a cohort study. British Journal of General Practice 63 | Wrong study design |
| Anonymous 2017 Assessment tool accurately predicts risk of bowel disease. Practice Nurse 47:4 | Wrong publication type |
| Abdullah, M. and Simadibrata, M. and Syam, A. F. and Wijayadi, T. and Fauzi, A. and Santi, A. and Rani, A. A. 2010 The accuracy of Fecal Immunochemical Test in early detection of colorectal cancer. Journal of Gastroenterology and Hepatology 2 | Wrong publication type |
| Adams, K. and Sideris, M. and Papagrigoriadis, S. 2014 Can we make 'Straight to Test' decisions in Two Week Wait (2WW) patients with the help of an Artificial Neural Network (ANN)? Colorectal Disease 2 | Wrong publication type |
| Adelstein, B. and Macaskill, P. and Turner, R. M. and Katelaris, P. H. and Irwig, L. 2014 How well do common symptoms predict colorectal cancer? Asia-Pacific Journal of Clinical Oncology 4 | Wrong publication type |
| Adelstein, B. A. and Irwig, L. and Macaskill, P. and Turner, R. M. and Chan, S. and Katelaris, P. H. 2009 Can bowel symptoms be used to identify patients with colorectal cancer? Journal of Gastroenterology and Hepatology | Wrong publication type |
| Ain, Q. and Widlak, M. and McCullough, P. and Bajwa, A. and Evans, C. and Arasaradnam, R. P. 2018 Faecal immunochemical testing (FIT) to diagnose significant disease in patients with lower gastrointestinal symptoms - does NICE NG 12 and DG 30 FIT all? Colorectal Disease 20 | Wrong publication type |
| Akolkar, D. B. and Patil, D. and Fulmali, P. et al. 2021 Analytical and clinical validation of the TruCheck platform for diagnostic triaging of symptomatic cases suspected of colorectal cancer. Journal of Clinical Oncology. Conference 39:3 | Wrong publication type |
| Alatise, O. I. and Bello, I. S. and Komolafe, A. O. and Adekanle, O. and Kingham, T. P. 2015 Identifying patients at high risk for colorectal cancer: A pilot study of colonoscopy in patients with rectal bleeding in a low-income country. Journal of Clinical Oncology. Conference 33:3 | Wrong publication type |
| Ardizzoia, A. and Parente, F. and Marino, B. et al. 2011 A combination of fecal tests for the detection of colon cancer: A new strategy for appropriate prioritization of referrals to colonscopy-A prospective Italian study. Journal of Clinical | Wrong publication type |

| | |
|---|---|
| Oncology. Conference: ASCO Annual Meeting 29:15 | |
| Arroll, B. 2011 The diagnostic value of symptoms for colorectal cancer in primary care. British Journal of General Practice 61 | Wrong publication type |
| Astin, M. and Griffin, T. and Neal, R. D. and Rose, P. and Hamilton, W. 2011 The diagnostic value of symptoms for colorectal cancer in primary care: a systematic review. British Journal of General Practice 61 | Wrong publication type |
| Au, S. and Miller, J. and Porteous, L. and Dunlop, M. G. and Maeda, Y. and Din, F. V. N. 2020 QCancer as a tool to stratify risk during the COVID-19 pandemic. Colorectal Disease 22 | Wrong publication type |
| Auge Fradera, J. M. and Roset, A. and Escudero, J. M. and Foj, L. and Filella, X. and Molina, R. 2014 Clinical utility of HM JACKarc for the detection of colorectal cancer and high-risk adenomas. Tumor Biology 1 | Wrong publication type |
| Auge, J. M. M. and Sandalinas, S. and Fernadez, E. 2017 Using fecal immunochemical test for hemoglobin in a quantitative way for the diagnosis of colorectal neoplasia. Tumour Biology 39 | Wrong publication type |
| Bai, Y. and Xu, C. and Zou, D. W. and Gao, J. and Li, Z. S. 2011 Diagnostic accuracy of features predicting lower gastrointestinal malignancy: a colonoscopy database review of 10,603 Chinese patients. Colorectal Disease 13:6 | Wrong analysis |
| Bailey, J. A. and Ibrahim, H. and Bunce, J. and Chapman, C. J. and Morling, J. R. and Simpson, J. A. and Humes, D. J. and Banerjea, A. 2021 Quantitative FIT stratification is superior to NICE referral criteria NG12 in a high-risk colorectal cancer population. Techniques in Coloproctology 25:10 | Wrong analysis |
| Bailey, J. A. and Weller, J. and Chapman, C. J. and Ford, A. and Hardy, K. and Oliver, S. and Morling, J. R. and Simpson, J. A. and Humes, D. J. and Banerjea, A. 2021 Faecal immunochemical testing and blood tests for prioritization of urgent colorectal cancer referrals in symptomatic patients: a 2-year evaluation. Bjs Open 5:2 | Wrong study design |
| Bailey, S. E. R. and Abel, G. A. and Atkins, A. and Byford, R. and Davies, S. J. and Mays, J. and McDonald, T. J. and Miller, J. and Neck, C. and Renninson, J. and Thomas, P. and Walter, F. M. and Warren, S. and Hamilton, W. 2021 Diagnostic performance of a faecal immunochemical test for patients with low-risk symptoms of colorectal cancer in primary care: an evaluation in the South West of England. British Journal of Cancer 124 | Wrong analysis |
| Barraclough, K. 2010 The predictive value of cancer symptoms in primary care. British Journal of General Practice 60 | Wrong publication type |
| Baty, V. and Soler-Michel, P. and Ponchon, T. 2017 Colorectal Cancer Screening, Fecal Immunochemical Tests, and New Oral Anticoagulants. American Journal of Medicine 130 | Wrong publication type |
| Bjerregaard, N. C. and Tottrup, A. and Sorensen, H. T. and Laurberg, S. 2007 Diagnostic value of self-reported symptoms in Danish outpatients referred | Wrong analysis |

| | |
|---|---|
| with symptoms consistent with colorectal cancer. Colorectal Disease 9:5 | |
| Bjerregaard, N. C. and Tottrup, A. and Sorensen, H. T. and Laurberg, S. 2009 Detection of colorectal cancer in symptomatic outpatients without visible rectal bleeding: Validity of the fecal occult blood test. Clinical Epidemiology 1 | Wrong analysis |
| Bond, A. and Greenwood, R. and Lewis, S. and Corfe, B. et al. 2019 Volatile organic compounds emitted from faeces as a biomarker for colorectal cancer. Alimentary Pharmacology & Therapeutics 49:8 | Wrong population |
| Bond, A. and Greenwood, R. and Lewis, S. and Corfe, B. and Sarkar, S. and Rooney, P. and Probert, C. 2016 The use of volatile organic compounds emitted from stool as a biomarker for colonic neoplasia. Gut 65 | Wrong population |
| Bond, A. D. and Burkitt, M. D. and Sawbridge, D. and Corfe, B. M. and Probert, C. S. 2016 Correlation between Faecal Tumour M2 Pyruvate Kinase and Colonoscopy for the Detection of Adenomatous Neoplasia in a Secondary Care Cohort. Journal of Gastrointestinal & Liver Diseases 25:1 | Wrong analysis |
| Bunce, J. and Ng, O. and Tangri, A. and Rogers, R. and Oliver, S. and Chapman, C. and Logan, R. A. and Humes, D. J. and Banerjea, A. 2018 Stratification of colorectal cancer risk in the two week wait pathway using FIT and anaemia: A service evaluation of the -getting FIT- pilot in Nottingham. Colorectal Disease 20 | Wrong publication type |
| Cade, D. 2008 Predictive value of common symptom combinations in diagnosing colorectal cancer. British Journal of Surgery 95:4 | Wrong publication type |
| Calanzani, N. and Pannebakker, M. M. and Tagg, M. and Walford, H. and Holloway, P. and De Wit, N. and Hamilton, W. and Walter, F. M. 2021 Use of the faecal immunochemical test for patients with lower risk symptoms of colorectal cancer in primary care: A pragmatic prospective study in the east of England. United European Gastroenterology Journal 9 | Wrong outcome |
| Calogero, A. and Cirillo, M. and Gennarelli, N. and Maio, N. and Buonomo, N. and Lobello, R. 2012 Cancer colon test: Evolution and prospects in colorectal cancer screening. Annals of Oncology 4 | Wrong publication type |
| Caron, M. and Lamarre, G. and Gregoire, P. and Simonyan, D. and Laflamme, N. 2018 The fecal immunochemical test (fit): Selected aspects regarding its effectiveness for colorectal cancer screening in Quebec City. Preventive Medicine Reports 12 | Wrong population |
| Chak, A. and Post, A. B. and Cooper, G. S. 1996 Clinical variables associated with colorectal cancer on colonoscopy: a prediction model. American Journal of Gastroenterology 91:12 | Wrong population |
| Chandrapalan, S. and Bosch, S. and Cubiella, J. and Guardiola, J. et al. 2021 Systematic review with meta-analysis: volatile organic compound analysis to improve faecal immunochemical testing in the | Wrong publication type |

| | |
|---|---|
| detection of colorectal cancer. Alimentary Pharmacology and Therapeutics 54 | |
| Chang, L. C. and Herzog, M. and Hardat, N. and Pamart, D. and Chiu, H. M. 2022 Association of circulating nucleosomes levels with FIT performance for advanced adenomas in a symptomatic population. Journal of Clinical Oncology. Conference 40:4 | Wrong publication type |
| Chapman, C. and Thomas, C. and Morling, J. and Tangri, A. and Oliver, S. and Simpson, J. A. and Humes, D. J. and Banerjea, A. 2020 Early clinical outcomes of a rapid colorectal cancer diagnosis pathway using faecal immunochemical testing in Nottingham. Colorectal Disease 22 | Wrong study design |
| Chapuis, P. H. and Goulston, K. J. and Dent, O. F. and Tait, A. D. 1985 Predictive value of rectal bleeding in screening for rectal and sigmoid polyps. British Medical Journal Clinical Research Ed. 22 | Wrong study design |
| Chen, C. and Chen, L. and Yang, G. and Li, Y. 2008 The application of C12 biochip in the diagnosis and monitoring of colorectal cancer: Systematic evaluation and suggestion for improvement. Journal of Postgraduate Medicine 54:3 | Wrong study design |
| Chen, K. C. and Chung, C. S. and Hsu, W. F. and Huang, T. Y. et al. 2018 Identification of risk factors for neoplastic colonic polyps in young adults with bloody stool in comparison with those without symptom. Journal of Gastroenterology & Hepatology 33:7 | Wrong analysis |
| Chen, Y. and Sun, H. and Sun, M. and Shi, C. and Sun, H. and Shi, X. and Ji, B. and Cui, J. 2021 Finding Colon Cancer- and Colorectal Cancer-Related Microbes Based on Microbe-Disease Association Prediction. Frontiers in Microbiology 12 | Wrong study design |
| Chisholm, E. M. and Marshall, R. J. and Brown, D. and Cooper, E. H. and Giles, G. R. 1986 The role of a questionnaire and four biochemical markers to detect cancer risk in a symptomatic population. British Journal of Cancer 53:1 | Wrong outcome |
| Christensen, I. J. and Brunner, N. and Dowell, B. and Davis, G. and Nielsen, H. J. and Newstead, G. and King, D. 2015 Plasma TIMP-1 and CEA as Markers for Detection of Primary Colorectal Cancer: A Prospective Validation Study Including Symptomatic and Non-symptomatic Individuals. Anticancer Research 35:9 | Wrong population |
| Christensen, I. J. and Rasmussen, L. and Ferm, L. and Nielsen, H. J. 2020 A Novel Panel of 92 Blood-Based Biomarkers for the Detection of Colorectal Cancer and High-Risk Adenomas in 784 Symptomatic Subjects. Gastroenterology 158 | Wrong publication type |
| Christensen, I. J. and Wilhelmsen, M. and Gawel, S. et al. 2016 Early detection of colorectal neoplasia: Combination of eight cancer-associated blood-based protein biomarkers. Journal of Clinical Oncology. Conference 34:4 | Wrong publication type |
| Clackett, W. and Barclay, S. T. and Stanley, A. J. and Cahill, A. 2021 The Value of Quantitative Faecal Immunochemical Testing as a Prioritisation Tool for the Endoscopic Investigation of Patients With Iron Deficiency. Frontiers in Medicine 8 | Wrong study design |

| | |
|---|---|
| Clackett, W. and Cahill, A. 2021 Id: 3526093 the Value of Quantitative Faecal Immunochemical Test (Qfit) as a Prioritisation Tool for Patients with Iron Deficiency Requiring Urgent Outpatient Investigation. Gastrointestinal Endoscopy 93 | Wrong analysis |
| Cooper, J. A. and Ryan, R. and Parsons, N. and Stinton, C. and Marshall, T. and Taylor-Phillips, S. 2020 The use of electronic healthcare records for colorectal cancer screening referral decisions and risk prediction model development. BMC Gastroenterology 20:1 | Wrong population |
| Corte, C. and Zhang, L. and Chen, J. and Westbury, S. and Shaw, J. and Yeoh, K. G. and Leong, R. 2016 Validation of the Asia Pacific Colorectal Screening (APCS) score in a Western population: An alternative screening tool. Journal of Gastroenterology & Hepatology 31:2 | Wrong population |
| Cowley, J. and Gunn, J. and Duthie, G. S. and Hartley, J. E. 2013 The use of knowledge discovery databases in the identification of patients with colorectal cancer. Colorectal Disease 1 | Wrong publication type |
| Croner, L. J. and Kao, A. and Benz, R. and Blume, J. E. and Dillon, R. and Wilcox, B. and Kairs, S. N. 2017 A new blood test for colorectal cancer in high-risk subjects. Clinical Chemistry 63 | Wrong publication type |
| Cruz, A. and Carvalho, C. M. and Cunha, A. and Crespo, A. and Iglesias, A. and Garcia-Nimo, L. and Freitas, P. P. and Cubiella, J. 2021 Faecal Diagnostic Biomarkers for Colorectal Cancer. Cancers 13:21 | Wrong population |
| Cubiella Fernandez, J. and Hernandez-Gomez, M. and Fernandez-Dominguez, M. J. et al. 2020 Validation of colorectal cancer prediction models in primary healthcare. United European Gastroenterology Journal 8 | Wrong publication type |
| Cubiella, J. and Digby, J. and Rodriguez-Moranta, F. and Bujanda, L. et al 2015 The fas (fecal hemoglobin concentration, age and sex) model: Development and external validation of a simple predictive model for colorectal cancer detection in symptomatic patients. United European Gastroenterology Journal 1 | Wrong publication type |
| Cubiella, J. and Vega, P. and Alves, M. T. and Salve, M. and Diaz-Ondina, M. et al 2014 Colonpredict study: Development and validation of a predictive model for colorectal cancer detection in symptomatic patients. United European Gastroenterology Journal 1 | Wrong publication type |
| Cunningham, A. and Jenkins, R. and Jenkins, C. and Davies, M. et al 2018 Raman spectroscopy as a blood based primary care diagnostic for colorectal cancer. Colorectal Disease 20 | Wrong publication type |
| D'Hondt, L. and Herzog, M. and Rahier, J. F. and Faugeras, L. et al. 2016 Detection of colorectal cancer and adenomas by epigenetic profiles of circulating nucleosomes: A pilot study with 58 subjects. European Journal of Cancer 61 | Wrong publication type |
| D'Souza, N. and Delisle, T. G. and Benton, S. and Chen, M. and Abulafi, M. 2021 Can fit rule out colorectal cancer in symptomatic patients? Results from the nice fit study. Gut 70. | Wrong publication type |

| | |
|---|---|
| D'Souza, N. and Georgiou Delisle, T. and Benton, S. and Chen, M. and Abulafi, M. 2020 FIT can rule out colorectal cancer in patients with high risk symptoms? diagnostic accuracy results of the faecal immunochemical test in 9822 patients in the NICE FIT study. Colorectal Disease 22 | Wrong publication type |
| D'Souza, N. and Georgiou Delisle, T. and Chen, M. and Benton, S. and Abulafi, M. and Group, Nice Fit Steering 2021 Faecal immunochemical test is superior to symptoms in predicting pathology in patients with suspected colorectal cancer symptoms referred on a 2WW pathway: a diagnostic accuracy study. Gut 70:6 | Wrong analysis |
| D'Souza, N. and Hicks, G. and Benton, S. and Abulafi, M. 2018 The Faecal Immunochemical Test - FIT - to rule out Bowel cancer in symptomatic patients. Colorectal Disease 20 | Wrong publication type |
| D'Souza, N. and Monahan, K. and Benton, S. C. and Wilde, L. and Abulafi, M. and Group, Nice Fit Steering 2021 Finding the needle in the haystack: the diagnostic accuracy of the faecal immunochemical test for colorectal cancer in younger symptomatic patients. Colorectal disease 23:10 | Wrong analysis |
| Dabbous, H. K. and Mohamed, Y. A. E. and El-Folly, R. F. and El-Talkawy, M. D. and Seddik, H. E. and Johar, D. and Sarhan, M. A. 2019 Evaluation of Fecal M2PK as a Diagnostic Marker in Colorectal Cancer. Journal of Gastrointestinal Cancer 50:3 | Wrong analysis |
| Dabos, K. and Gousi, T. and Navrozoglou, A. and Kodogiorgou, E. and Papadopoulos, M. 2010 The absence of anaemia and bleeding per rectum in patients over 60 years of age exclude a diagnosis of colorectal cancer. Annals of Oncology 6 | Wrong publication type |
| Davis, G. and Gawel, S. and Wilhelmsen, M. and Rassussen, L. and Martens, F. and Heiboer, A. and Nielsen, H. 2015 Prototype biomarker panel algorithm performance for aid in early colorectal cancer detection. Tumor Biology 36 | Wrong publication type |
| Davis, G. J. and Gawel, S. and Wilhelmsen, M. and Rasmussen, L. and Christensen, I. J. and Martens, F. and Heijboer, A. and Nielsen, H. J. 2015. Prototype biomarker panel algorithms for aiding in early colorectal cancer (CRC) detection. Tumor Biology 37 | Wrong publication type |
| Debernardi, C. and Libera, L. and Berrino, E. and Sahnane, N. and Chiaravalli, A. M. and Laudi, C. and Berselli, M. and Sapino, A. and Sessa, F. and Venesio, T. and Furlan, D. 2021 Evaluation of global and intragenic hypomethylation in colorectal adenomas improves patient stratification and colorectal cancer risk prediction. Clinical Epigenetics 13:1 | Wrong population |
| Delisle, T. G. and D'Souza, N. and Chen, M. and Benton, S. and Abulafi, M. 2020 Can FIT rule out colorectal cancer in symptomatic patients? Diagnostic test accuracy results from 9,822 patients in the NICE FIT study. Journal of Clinical Oncology. Conference 38:15 | Wrong publication type |
| Diaz Ondina, M. and Blanco Vila, M. I. and Ceballos Ogando, S. and Salve Bouzo, M. and Macia | Wrong publication type |

| | |
|---|---|
| Cortinas, P. and Cubiella Fernandez, J. 2014 Clinical or analytical criteria for colorectal cancer (CRC) detection in symptomatic patients? A diagnostic tests study. Clinical Chemistry and Laboratory Medicine 1 | |
| Digby, J. and Steele, R. J. C. and Strachan, J. A. and Mowat, C. 2014 Stool tests can potentially rule out significant bowel disease in symptomatic patients in primary care. United European Gastroenterology Journal 1 | Wrong publication type |
| Digby, J. and Strachan, J. A. and Fraser, C. G. and Steele, R. and Mowat, C. 2017 Validation of the utility of a faecal immunochemical test for haemoglobin (FIT) in patients presenting to primary care with new bowel symptoms. United European Gastroenterology Journal 5 | Wrong publication type |
| Dillon, R. and Blimline, M. and You, J. and Yee, A. and Levy, J. et al. 2017 Analytical validation of a blood-based colorectal cancer and advanced adenoma risk assessment LDT. Clinical Chemistry 63 | Wrong publication type |
| Druce, P. and Calanzani, N. and Snudden, C. and Milley, K. et al. 2021 Identifying Novel Biomarkers Ready for Evaluation in Low-Prevalence Populations for the Early Detection of Lower Gastrointestinal Cancers: A Systematic Review and Meta-Analysis. Advances in Therapy 38 | Wrong publication type |
| du Toit, J. 2006 Assessing patients for risk of colorectal cancer in primary care. Practitioner 250 | Wrong publication type |
| Elbeltagi, A. H. and Boxall, P. and Salama, M. and Roos, J. 2021 The yield of FIT in the detection of colorectal cancer within a fast track pathway at York and Scarborough Teaching Hospitals. Colorectal Disease 23 | Wrong publication type |
| Elbeltagi, A. H. and Boxall, P. and Salama, M. and Roos, J. and Lim, M. 2021 The yield of FIT in the detection of colorectal cancer within a fast track pathway at York. Colorectal Disease 23 | Wrong publication type |
| Ellis, B. G. and Flashman, K. G. and Asiimwe, A. and Thompson, M. R. 2021 Simple clinical algorithm to stratify the risk of having bowel cancer in primary care-better than FIT? Colorectal disease 23 | Wrong publication type |
| Farrugia, A. and Widlak, M. and Evans, C. and Smith, S. C. and Arasaradnam, R. 2020 Faecal immunochemical testing (FIT) in symptomatic patients: what are we missing? Frontline Gastroenterology 11:1 | Wrong study design |
| Fauzi, A. and Abdullah, M. and Simadibrata, M. and Syam, A. F. and Wijayadi, T. and Rani, A. A. 2011 The accuracy of fecal immunochemical test in early detection of colorectal cancer. Digestion 83 | Wrong publication type |
| Fernandez-Banares, F. and Cleries, R. and Boadas, J. and Ribes, J. et al. 2018 Prediction of advanced colonic neoplasm in symptomatic patients: A scoring system based on the faecal immunological test to prioritize colonoscopy in the fast-track referral system (colonofit study). United European Gastroenterology Journal 6 | Wrong publication type |
| Forbes, N. and Cooray, M. and Hackett, M. and Shah, N. 2020 An Electronic Clinical Decision-Making Tool for Patients with Suspected Colorectal | Wrong analysis |

| | |
|---|---|
| Cancer-Preliminary Evaluation in Patients Presenting with Rectal Bleeding. Journal of the Canadian Association of Gastroenterology 3:5 | |
| Forbes, Nauzer and Hilsden, Robert J. and Heitman, Steven J. 2020 The appropriate use of fecal immunochemical testing. CMAJ 192:3 | Wrong publication type |
| Fraser, C. G. 2020 Use of fecal immunochemical testing in patients presenting in primary care with lower GI symptoms. CMAJ 192:14 | Wrong publication type |
| Fraser, C. G. and Strachan, J. A. 2016 A nicer approach to the use of â€˜faecal occult blood testing in assessment of the symptomatic. Annals of Clinical Biochemistry 53:1 | Wrong publication type |
| Friedland, S. and Pan, J. Y. and Watson, D. and Chen, Y. et al. 2021 Development and clinical validation of a blood test for early detection of colorectal adenomas and cancer. Journal of Clinical Oncology. Conference 39:3 | Wrong population |
| Gao, J. and Zhang, L. and Yu, G. and Qu, G. and Li, Y. and Yang, X. 2020 Model with the GBDT for colorectal adenoma risk diagnosis. Current Bioinformatics 15 | Wrong population |
| Garside, H. and Ball, G. and Thom, F. and Burton, T. and Gunn, R. and Maeda, Y. and Dunlop, M. and Din, F. 2020 Cancer detection using quantitative faecal immunochemical test in symptomatic patients. Colorectal disease 22 | Wrong publication type |
| Gawel, S. 2015 Statistical approaches in developing screening-signature positive algorithms for risk stratification: Application to the early detection of colorectal cancer trial. Tumor Biology 36 | Wrong publication type |
| Gerrard, A. D. and Maeda, Y. and Gunn, F. and Miller, J. and Pattenden, R. and Dunlop, M. G. and Din, F. V. N. 2021 Double Quantitative Faecal Immunochemical Testing (qFIT) in Symptomatic Patients. Colorectal Disease 23 | Wrong publication type |
| Ghajari, H. and Sadeghi, A. and Khodakarim, S. and Zali, M. and Nazari, S. S. H. 2021 Designing a Predictive Model for Colorectal Neoplasia Diagnosis Based on Clinical and Laboratory Findings in Colonoscopy Candidate Patients. Journal of Gastrointestinal Cancer 1 | Wrong population |
| Griffiths, E. K. and Schapira, D. V. 1991 Serum ferritin and stool occult blood and colon cancer screening. Cancer Detection & Prevention 15:4 | Wrong population |
| Guery, E. and Canoui-Poitrine, F. and Costes, L. and Cavicchi, M. and Peyvandi, S. and Barral, J. C. and Gornet, J. M. and Amiot, A. and Sobhani, I. 2015 Performance of a blood methylated mutitarget DNA test (colohybritest) for the diagnosis of colorectal (CRC) cancer: A transversal study on 878 individuals. Gastroenterology 1 | Wrong population |
| Guo, S. and Li, L. and Xu, B. and Li, M. and Zeng, Q. and Xiao, H. and Xue, Y. and Wu, Y. and Wang, Y. and Liu, W. and Zhang, G. 2018 A simple and novel fecal biomarker for colorectal cancer: Ratio of Fusobacterium nucleatum to probiotics populations, based on their antagonistic effect. Clinical Chemistry 64:9 | Wrong population |

| | |
|---|---|
| Hamarneh, Z. and Symonds, E. L. and Cock, C. 2018 Does the presence of symptoms increase the risk of colorectal cancer in patients with a positive fecal immunochemical test result? Journal of Gastroenterology and Hepatology 33 | Wrong publication type |
| Hamilton, W. and Lancashire, R. and Sharp, D. and Peters, T. J. and Cheng, K. and Marshall, T. 2009 The risk of colorectal cancer with symptoms at different ages and between the sexes: a case-control study. BMC Medicine 7 | Wrong study design |
| Hamilton, W. and Lancashire, R. and Sharp, D. and Peters, T. J. and Cheng, K. K. and Marshall, T. 2008 The importance of anaemia in diagnosing colorectal cancer: a case-control study using electronic primary care records. British Journal of Cancer 98:2 | Wrong analysis |
| Hardcastle, J. D. and Pye, G. 1989 Screening for colorectal cancer: a critical review. World Journal of Surgery 13:1 | Wrong publication type |
| Hatzidaki, E. and Iliopoulos, A. and Papasotiriou, I. 2021 A Novel Method for Colorectal Cancer Screening Based on Circulating Tumor Cells and Machine Learning. Entropy 23:10 | Wrong population |
| Herzog, M. and Rasmussen, L. and Christensen, I. J. et al. 2014 One thousand-patient study of detection of colorectal cancer and colorectal polyps by serum ELISA of altered epigenetic signatures in circulating cell-free nucleosomes. Anticancer Research 34 | Wrong publication type |
| Hicks, G. and D'Souza, N. and Georgiou Delisle, T. and Chen, M. and Benton, S. C. and Abulafi, M. and group, Nice Fit steering 2021 Using the faecal immunochemical test in patients with rectal bleeding: evidence from the NICE FIT study. Colorectal Disease 23:7 | Wrong study design |
| Hijos, G. and Lue, A. and Sostres, C. and Barra, M. V. and Mascialino, B. and Andalucia, C. and Puente, J. J. and Lanas, A. and Gomollon, F. 2018 Combination of Quantitative Faecal Occult Blood Test and Fecal Calprotectin is a Cost-Effective Strategy to Avoid Non Pathological Colonoscopies in Symptomatic Patients. Gastroenterology 154 | Wrong outcome |
| Hijos, G. and Velamazan, R. and Navarro, M. and Arechavaleta, S. and Chueca, E. and Lue, A. and Sostres, C. and Gomollon, F. and Lanas, A. 2019 A patient self-made one step quick faecal test reduces unnecessary colonoscopies and prioritizes high risk symptomatic patients. United European Gastroenterology Journal 7 | Wrong publication type |
| Hijos, G. and Velamazan, R. and Navarro, M. and Chueca, E. and Arechavaleta, S. and Lue, A. and Sostres, C. and Gomollon, F. and Lanas, A. 2019 A Patient Self-Made One Step Quick Faecal Test Reduces Unnecessary Colonosopies and Prioritizes High Risk Symptomatic Patients. Gastroenterology 156 | Wrong publication type |
| Hirata, I. 2020 Evaluation of the usefulness of the simultaneous assay of fecal hemoglobin (Hb) and transferrin (Tf) in colorectal cancer screening - for the establishment of the Hb and Tf two-step cutoff assay (HTTC assay). Diagnosis 7:2 | Wrong population |

| | |
|---|---|
| Hirayama, Y. and Watanabe, O. and Nakamura, M. and Yamamura, T. and Goto, H. 2015 Predictive factor of colorectal cancer for the elderly aged 85 years and older with positive fecal occult blood test. Gastrointestinal Endoscopy 1 | Wrong publication type |
| Hogberg, C. and Gunnarsson, U. and Cronberg, O. and Thulesius, H. and Lilja, M. and Jansson, S. 2020 Qualitative faecal immunochemical tests (FITs) for diagnosing colorectal cancer in patients with histories of rectal bleeding in primary care: a cohort study. International Journal of Colorectal Disease 35:11 | Wrong study design |
| Hogberg, C. and Karling, P. and Rutegard, J. and Lilja, M. 2020 BMC Family Practice 21:1 | Wrong outcome |
| Holtedahl, K. and Borgquist, L. and Donker, G. A. and Buntinx, F. et al. Symptoms and signs of colorectal cancer, with differences between proximal and distal colon cancer: a prospective cohort study of diagnostic accuracy in primary care. BMC Family Practice 22:1 | Wrong analysis |
| Holtedahl, K. and Hjertholm, P. and Borgquist, L. et al. 2018 Abdominal symptoms and cancer in the abdomen: prospective cohort study in European primary care. British Journal of General Practice 68 | Wrong analysis |
| Hurst, N. G. and Stocken, D. D. and Wilson, S. and Keh, C. and Wakelam, M. J. and Ismail, T. 2007 Elevated serum matrix metalloproteinase 9 (MMP-9) concentration predicts the presence of colorectal neoplasia in symptomatic patients. British Journal of Cancer 97:7 | Wrong outcome |
| Hussan, H. and Zhao, J. and Stanich, P. P. and Tabung, F. K. and Gray, D. M. and Ma, Q. and Clinton, S. 2021 Application Of Machine Learning To Enhance Colorectal Cancer Diagnosis And Prevention Strategies For Young Adults Aged 35-50 Years. Gastroenterology 160 | Wrong publication type |
| Ibrahim, J. and Op de Beeck, K. and Fransen, E. and Croes, L. et al 2019 Methylation analysis of Gasdermin E shows great promise as a biomarker for colorectal cancer. Cancer Medicine 8:5 | Wrong population |
| Imperiale, T. F. and Monahan, P. O. and Stump, T. E. and Ransohoff, D. F. 2021 Derivation and validation of a predictive model for advanced colorectal neoplasia in asymptomatic adults. Gut 70:6 | Wrong population |
| Jensen, S. O. and Orntoft, M. B. W. and Ogaard, N. and Kristensen, H. et al 2018 Novel DNA methylation biomarkers show high sensitivity and specificity for blood-based detection of colorectal cancer - A clinical biomarker discovery and validation study. Cancer Research. Conference 78:13 | Wrong publication type |
| Jiang, P. and Wu, S. and Luo, Q. and Zhao, X. M. and Chen, W. H. 2021 Metagenomic Analysis of Common Intestinal Diseases Reveals Relationships among Microbial Signatures and Powers Multidisease Diagnostic Models. Msystems 6:3 | Wrong population |
| Johnstone, M. and Burton, P. and Kourounis, G. and Winter, J. et al. 2022 Combining the quantitative fecal immunochemical test and full blood count to rule out colorectal cancer in a symptomatic patient | Wrong publication type |

| | |
|---|---|
| referral pathway. Journal of Clinical Oncology. Conference 40:4 | |
| Jones, J. J. and Wilcox, B. E. and Benz, R. W. and Babbar, N. et al. 2016 A plasma-based protein marker panel for colorectal cancer detection identified by multiplex targeted mass spectrometry. Clinical Colorectal Cancer 15:2 | Wrong population |
| Jones, R. and Latinovic, R. and Charlton, J. and Gulliford, M. C. 2007 Alarm symptoms in early diagnosis of cancer in primary care: cohort study using General Practice Research Database. BMJ 334 | Wrong analysis |
| Karimi, N. and Feizi, M. A. H. and Safaralizadeh, R. et al. 2019 Serum overexpression of miR-301a and miR-23a in patients with colorectal cancer. Journal of the Chinese Medical Association 82:3 | Wrong analysis |
| Kilincalp, S. and Coban, S. and Akinci, H. and Hamamc, M. et al. 2015 Neutrophil/lymphocyte ratio, platelet/lymphocyte ratio, and mean platelet volume as potential biomarkers for early detection and monitoring of colorectal adenocarcinoma. European Journal of Cancer Prevention 24 | Wrong analysis |
| Kinar, Y. and Akiva, P. and Choman, E. and Kariv, R. and Shalev, V. and Levin, B. and Narod, S. A. and Goshen, R. 2017 Performance analysis of a machine learning flagging system used to identify a group of individuals at a high risk for colorectal cancer. PLoS ONE 12:2 | Wrong study design |
| Kleif, J. and Jorgensen, L. N. and Hendel, J. W. and Madsen, M. R. et al. 2022 Early detection of colorectal neoplasia: application of a blood-based serological protein test on subjects undergoing population-based screening. British Journal of Cancer 28 | Wrong population |
| Klimovskij, M. and Khan, A. and Harshen, R. 2019 Is faecal immunochemical test (FIT) better than symptoms described in NG12 guidelines to prioritize colorectal cancer referrals? Colorectal Disease 21 | Wrong publication type |
| Kromodikoro, I. and Nyhlin, N. and Van Nieuwenhoven, M. 2020 The swedish standardized course of care-colorectal cancer prevalence and predictive value of entry criteria. United European Gastroenterology Journal 8 | Wrong publication type |
| Lacy-Colson, J. and Norwood, M. and Murray, C. and Booth, J. 2015 Protein biomarkers in the rectal mucosa: A novel test for colorectal cancer? Gut 1 | Wrong publication type |
| Lanas, A. and Quintero, E. and Cubiella, J. and Martinez, R. J. and Cacho Acosta, G. and Poves, C. and Diaz Tasende, J. and Medeiros, I. and Balaguer, F. 2020 Simultaneous testing of fecal hemoglobin and calprotectin predicts the detection of significant colorectal disease in the work-up of symptomatic primary-care patients: The advantage study. United European Gastroenterology Journal 8 | Wrong publication type |
| Laurell, A. A. and Miller, J. and Garside, H. and Douglas, S. L. and McTeir, K. E. and Maeda, M. Y. and Dunlop, G. and Din, F. V. N. 2020 Cancer detection using the quantitative faecal immunochemical test in symptomatic patients. Colorectal disease 22 | Wrong publication type |

| | |
|---|---|
| Lawrenson, R. and Logie, J. and Marks, C. 2006 Risk of colorectal cancer in general practice patients presenting with rectal bleeding, change in bowel habit or anaemia. European Journal of Cancer Care 15:3 | Wrong study design |
| Lazebnik, L. and Prilepskaya, S. and Baryshnikov, E. and Parfenov, A. and Kosacheva, T. 2010 Prevalence of colon diverticulosis, colorectal polyps and colorectal cancer among adult population of Moscow city with chronic constipation and alarm symptoms (Results of the epidemiological study MUZA). Journal of Gastroenterology and Hepatology | Wrong publication type |
| Leis, V. M. and Hughes, M. L. and Williams, C. B. and Neumaster, T. D. and Ludwig, D. J. and Fontenelle, L. J. 2001 Risk factors predictive of positive findings at colonoscopy. Current Surgery 58:2 | Wrong study design |
| Li, J. B. and Qiu, Z. Y. and Deng, Y. X. and Li, Y. and Lin, Z. C. et al. 2022 Factors associated with positive predictive value of preliminary screening in a two-step screening strategy for colorectal neoplasms in China. Discover Oncology 13 | Wrong population |
| Li, W. and Zhao, L. Z. and Ma, D. W. and Wang, D. Z. and Shi, L. et al. 2018 Predicting the risk for colorectal cancer with personal characteristics and fecal immunochemical test. Medicine 97:18 | Wrong population |
| Liang, J. Q. and Wong, S. H. and Szeto, C. H. and Chu, E. S. et al. 2021 Fecal microbial DNA markers serve for screening colorectal neoplasm in asymptomatic subjects. Journal of Gastroenterology & Hepatology 36:4 | Wrong population |
| Liang, J. Q. and Wong, S. H. and Szeto, C. H. and Chu, E. S.et al 2019 Fecal Microbial Markers in Colorectal Cancer Screening for Symptomatic and Asymptomatic Subjects. Gastroenterology 156 | Wrong publication type |
| Lidgard, G. P. and Domanico, M. J. and Bruinsma, J. J. and Light, J. et al. 2012 An optimized molecular stool test for colorectal cancer screening: Evaluation of an automated analytic platform and logistic algorithm. Cancer Prevention Research. Conference: 11th Annual AACR International Conference on Frontiers in Cancer Prevention Research. Anaheim, CA United States. Conference Publication. 5:11 | Wrong publication type |
| Liu, F. and Long, Q. and He, H. and Dong, S. and Zhao, L. and Zou, C. and Wu, W. 2021 Combining the Fecal Immunochemical Test with a Logistic Regression Model for Screening Colorectal Neoplasia. Frontiers in Pharmacology 12 | Wrong population |
| Loktionov, A. and Soubieres, A. and Bandaletova, T. and Francis, N. and Allison, J. and Sturt, J. and Mathur, J. and Poullis, A. 2020 Biomarker measurement in non-invasively sampled colorectal mucus as a novel approach to colorectal cancer detection: screening and triage implications. British Journal of Cancer 123:2 | Wrong analysis |
| Loo, K. F. and Cock, C. and Young, G. and Fraser, R. and Symonds, E. 2020 Predicting advanced colorectal neoplasia using quantitative fecal immunochemical testing in symptomatic patients in | Wrong publication type |

| | |
|---|---|
| the Australian population. Journal of Gastroenterology and Hepatology (Australia) 35 | |
| Lord, A. R. and Simms, L. A. and Brown, A. and Hanigan, K. et al. 2018 Development and evaluation of a risk assessment tool to improve clinical triage accuracy for colonoscopic investigations. BMC Cancer 18:1 | Wrong study design |
| Lowenmark, T. and Lofgren-Burstrom, A. and Zingmark, C. et al 2020 Parvimonas micra as a putative non-invasive faecal biomarker for colorectal cancer. Scientific Reports 10:1 | Wrong analysis |
| Lumachi, F. and Chiara, G. and Tozzoli, R. and Lo Re, G. and Basso, S. 2016 Carcinoembryonic antigen (CEA) and the carcinoma antigen 19-9 (CA 19-9) together in early diagnosis of Stage I-II colorectal adenocarcinoma. A case-control study. Annals of Oncology 27 | Wrong publication type |
| Luo, D. and Yang, J. and Liu, J. and Yong, X. and Wang, Z. 2022 Identification of four novel hub genes as monitoring biomarkers for colorectal cancer. Hereditas 159:1 | Wrong study design |
| Luo, H. and Shen, K. and Li, B. and Li, R. and Wang, Z. and Xie, Z. 2020 Clinical significance and diagnostic value of serum NSE, CEA, CA19-9, CA125 and CA242 levels in colorectal cancer. Oncology Letters 20:1 | Wrong study design |
| MacDonald, S. and MacDonald, L. and Godwin, J. and Macdonald, A. and Thornton, M. 2021 The diagnostic accuracy of the faecal immunohistochemical test in identifying significant bowel disease in a symptomatic population. Colorectal Disease 19 | Wrong analysis |
| Mahadavan, L. and Daniels, I. and Cotter, D. and Loktionov, A. and Llewelyn, A. and Shore, A. and Hamilton, W. 2010 The diagnostic accuracy of colonocyte DNA quantification in colorectal cancer diagnosis using a novel device. Gut 59 | Wrong publication type |
| Mahadavan, L. and Daniels, I. and Loktionov, A. and Llewelyn, A. and Shore, A. and Hamilton, W. 2010 Preliminary results of colonocyte quantification in colorectal cancer diagnosis. Diseases of the Colon and Rectum 53 | Wrong publication type |
| Majumdar, S. R. and Fletcher, R. H. and Evans, A. T. 1999 How does colorectal cancer present? Symptoms, duration, and clues to location. American Journal of Gastroenterology 94:10 | Wrong population |
| Malagon, M. and Ramio-Pujol, S. and Serrano, M. et al. 2018 Raid-CRC: A novel non-invasive tool for colorectal cancer screening based on bacterial signatures capable of reducing the faecal immunochemical test false-positive results. United European Gastroenterology Journal 6 | Wrong publication type |
| Mant, A. and Bokey, E. L. and Chapuis, P. H. and Killingback, M. and Hughes, W. and Koorey, S. G. and Cook, I. and Goulston, K. J. and Dent, O. F. 1989 Rectal bleeding. Do other symptoms aid in diagnosis? Diseases of the Colon & Rectum 32:3 | Wrong study design |
| Meng, W. and Cai, S. R. and Zhou, L. and Dong, Q. and Zheng, S. and Zhang, S. Z. 2009 Performance value of high risk factors in colorectal cancer | Wrong population |

| | |
|---|---|
| screening in China. World Journal of Gastroenterology 15:48 | |
| Miyoshi, H. and Oka, M. and Sugi, K. and Saitoh, O. and Katsu, K. and Uchida, K. 2000 Accuracy of detection of colorectal neoplasia using an immunochemical occult blood test in symptomatic referred patients: comparison of retrospective and prospective studies. Internal Medicine 39:9 | Wrong study design |
| Morales Arraez, D. E. and Hernandez, G. and Carrillo, M. and Adrian, Z. and Gimeno, A. Z. and Quintero, E. 2018 Role of faecal immunochemical testing in the diagnostic workup of patients with iron deficiency anaemia. United European Gastroenterology Journal 6. | Wrong outcome |
| Nakama, H. and Kayano, T. and Katsuura, T. et al. 1999 Comparison of predictive value for colorectal cancer in subjects with and without rectal bleeding. Hepato-Gastroenterology 46:27 | Wrong population |
| Nakama, H. and Zhang, B. and Abdul Fattah, A. S. and Kamijo, N. and Fukazawa, K. 2000 Relationships between a sign of rectal bleeding and the results of an immunochemical occult blood test, and colorectal cancer. European Journal of Cancer Prevention 9:5 | Wrong population |
| Navarro, M. and Hijos, G. and Ramirez, T. and Omella, I. and Fuentes, P. C. L. and Lanas, A. 2019 Faecal haemoglobin concentration, a good predictor of risk of advanced colorectal neoplasia in symptomatic and asymptomatic patients. 6 | Wrong analysis |
| Navarro, M. and Omella, I. and Carrera, P. and Hijos, G. and Roncales, P. and Sopena, F. and Puente, J. J. and Ferrandez, A. and Lanas, A. 2017 Fecal hemoglobin concentration, a good predictor of risk of advanced colorectal neoplasia in symptomatic and asymtomatic patients. Gastroenterology 152 | Wrong analysis |
| Nielsen, S. and Willenbrock, H. and Fog, J. U. and Stenvang, J. et al 2011 Validation of a plasma based miRNA PCR test for early detection of colorectal cancer. European Journal of Cancer. Conference: EORTC NCI ASCO Annual Meeting on 'Molecular Markers in Cancer' 47 | Wrong publication type |
| Niv, Y. and Sperber, A. D. 1995 Sensitivity, specificity, and predictive value of fecal occult blood testing (Hemoccult II) for colorectal neoplasia in symptomatic patients: a prospective study with total colonoscopy. American Journal of Gastroenterology 90:11 | Wrong analysis |
| Orntoft, M. B. and Nielsen, H. J. and Orntoft, T. F. and Andersen, C. L. and Danish Study Group on Early Detection of Colorectal, Cancer 2015 Performance of the colorectal cancer screening marker Sept9 is influenced by age, diabetes and arthritis: a nested case-control study. BMC Cancer 15 | Wrong analysis |
| Parente, F. and Marino, B. and Perna, F. and Saracino, I. and Zullo, A. et al 2010 Multiple faecal tests (colon panel) for the detection of colon cancer: A new strategy for appropriate prioritization of screening referrals? Preliminary experience in Italy. Digestive and Liver Disease 2 | Wrong publication type |

| | |
|---|---|
| Parente, F. R. and Marino, B. and Perna, F. and Saracino, I. M. et al 2010 Multiple faecal tests (colon panel) for the detection of colon cancer: A new strategy for appropriate prioritization of screening referrals? Preliminary experience in Italy. Gastroenterology 1 | Wrong publication type |
| Parsirad, M. and Aletaha, N. and Avanaki, F. A. and Farahvash, M. J. and Fazeli, M. S. and Zebardast, J. 2020 Investigating the relationship between red blood cell distribution width and early detection of colorectal cancer and colonic polyps. Journal of Kerman University of Medical Sciences 27 | Wrong analysis |
| Pasanen, P. and Eskelinen, M. and Kulju, A. and Penttila, I. and Janatuinen, E. and Alhava, E. 1995 Tumour-associated trypsin inhibitor (TATI) in patients with colorectal cancer: a comparison with CEA, CA 50 and CA 242. Scandinavian Journal of Clinical & Laboratory Investigation 55:2 | Wrong population |
| Pin Vieito, N. and Garcia Nimo, L. and Bujanda Fernandez De Pierola, L. et al. 2018 Accuracy of quantitative fecal immunochemical test to detect colorectal cancer. real-life data in 22,819 patients from the general practice setting in Spain. United European Gastroenterology Journal 6 | Wrong publication type |
| Pin Vieito, N. and Iglesias, M. J. and Remedios-Espino, D. et al. 2020 Predictive value of carcinoembryonic antigen in symptomatic patients without colorectal cancer. post-hoc analysis within colonpredict cohort. United European Gastroenterology Journal 8 | Wrong outcome |
| Pin Vieito, N. and Zarraquinos, S. and Cubiella, J. 2018 Systematic review and meta-analysis: Diagnostic accuracy of quantitative fecal immunochemical tests for colorectal cancer detection in symptomatic patients. United European Gastroenterology Journal 6 | Wrong publication type |
| Pin Vieito, N. and Zarraquinos, S. and Cubiella, J. 2019 High-risk symptoms and quantitative faecal immunochemical test accuracy: Systematic review and meta-analysis. World Journal of Gastroenterology 25:19 | Wrong publication type |
| Pin-Vieito, N. and Iglesias, M. J. and Remedios, D. and Alvarez-Sanchez, V. et al. 2020 Predictive Value of Carcinoembryonic Antigen in Symptomatic Patients without Colorectal Cancer: A Post-Hoc Analysis within the COLONPREDICT Cohort. Diagnostics 10:12 | Wrong analysis |
| Pin-Vieito, N. and Iglesias, M. J. and Remedios, D. et al. 2020 Retrospective cohort study: Risk of gastrointestinal cancer in a symptomatic cohort after a complete colonoscopy: Role of faecal immunochemical test. World Journal of Gastroenterology 26:1 | Wrong analysis |
| Pin-Vieito, N. and Iglesias, M. J. and Remedios, D. and Rodriguez-Alonso, L. et al. 2020 Risk of gastrointestinal cancer in a symptomatic cohort after a complete colonoscopy: Role of faecal immunochemical test. World Journal of Gastroenterology 26:1 | Wrong analysis |

| | |
|---|---|
| Quach, D. T. 2012 Evaluation of Asia-Pacific colorectal screening score in predicting risk of advanced colorectal neoplasia in Vietnamese with lower bowel symptoms. Journal of Gastroenterology and Hepatology 5 | Wrong publication |
| Rasmussen, S. and Haastrup, P. F. and Balasubramaniam, K. and Elnegaard, S. et al. 2019 Predictive values of colorectal cancer alarm symptoms in the general population: a nationwide cohort study. British Journal of Cancer 120:6 | Wrong analysis |
| Robertson, R. and Campbell, C. and Weller, D. P. and Elton, R. et al. 2006 Predicting colorectal cancer risk in patients with rectal bleeding. British Journal of General Practice 56 | Wrong analysis |
| Rodriguez, M. M. and Ramio-Pujol, S. and Serrano, M. et al. 2018 Raid-Crc: A Novel Non-Invasive Tool for Colorectal Cancer Screening Based on Bacterial Signatures. Gastroenterology 154 | Wrong publication type |
| Saldanha, J. D. and Moug, S. J. and Linton, K. and Diament, R. H. 2013 Symptoms do not predict colorectal cancer in an FOB screened population. Scottish Medical Journal 58:2 | Wrong population |
| Sewitch, M. J. and Joseph, L. and Hilsden, R. J. and Bitton, A. 2011 Screening colonos copy algorithms not accurate enough for use in research. American Journal of Epidemiology 11 | Wrong outcome |
| Shen, J. and Jin, G. and Zhang, Z. and Zhang, J. et al. 2022 A multiple-dimension model for microbiota of patients with colorectal cancer from normal participants and other intestinal disorders. Applied Microbiology and Biotechnology. | Wrong analysis |
| Shen, X. and Xue, Y. and Cong, H. and Wang, X. and Fan, Z. and Cui, X. and Ju, S. 2020 Circulating lncRNA DANCR as a potential auxillary biomarker for the diagnosis and prognostic prediction of colorectal cancer. Bioscience Reports 40:3 | Wrong population |
| Siddique, K. and Titu, L. V. and Walsh, C. J. and Anderson, J. A. and Smith, D. J. 2013 A predictive risk score for colorectal cancer using a weighted numerical score and marker of tumour metabalone. Surgical Endoscopy and Other Interventional Techniques 1 | Wrong publication type |
| Simons, Ccjm and Schouten, L. J. and Weijenberg, M. P. and Goldbohm, R. A. and van den Brandt, P. A. 2010 Bowel movement and constipation frequencies and the risk of colorectal cancer among men in the Netherlands Cohort Study on Diet and Cancer. American Journal of Epidemiology 172:12 | Wrong population |
| So, B. and Yau, Y. and Kariyawasam, V. and Leong, R. 2019 Prospective study of risk score strategies in the prediction of advanced colorectal neoplasia at colonoscopy. Gut 68 | Wrong publication type |
| So, B. and Yau, Y. and Kariyawasam, V. and Leong, R. 2019 Prospective study of risk score strategies in the prediction of advanced colorectal neoplasia at colonoscopy. Turkish Journal of Gastroenterology 30 | Wrong publication type |
| So, B. and Yau, Y. and Kariyawasam, V. and Leong, R. W. 2018 Prospective study of risk score strategies in the prediction of advanced colorectal neoplasia at | Wrong publication type |

| | |
|---|---|
| colonoscopy. Journal of Gastroenterology and Hepatology 33 | |
| So, B. and Yau, Y. and Kariyawasam, V. and Leong, R. W. 2019 Prospective study of risk score strategies in the prediction of advanced colorectal neoplasia at colonoscopy. Swiss Medical Weekly 149 | Wrong publication type |
| Stapley, S. and Rubin, G. and Shephard, E. and Alsina, D. and Hamilton, W. 2016 Identification and quantification of the clinical features of inflammatory bowel disease and colorectal cancer in patients aged 550 years: Who needs urgent colonoscopy. United European Gastroenterology Journal 4 | Wrong outcome |
| Stelling, H. P. and Maimon, H. N. and Smith, R. A. and Haddy, R. I. and Markert, R. J. 1990 A comparative study of fecal occult blood tests for early detection of gastrointestinal pathology. Archives of Internal Medicine 150 | Wrong population |
| Stonestreet, J. and Chandrapalan, S. and Woolley, D. and Uthman, O. and Arasaradnam, R. P. 2019 Systematic review and meta-analysis: Diagnostic accuracy of faecal immuno-chemical testing for haemoglobin (FIT) in detecting colorectal cancer for both symptomatic and screening population. Acta Gastro-Enterologica Belgica 82 | Wrong publication type |
| Storm, L. and Christensen, I. J. and Jensenius, J. C. and Nielsen, H. J. and Thiel, S. and Danish Study Group on Early Detection of Colorectal, Cancer 2015 Evaluation of complement proteins as screening markers for colorectal cancer. Cancer Immunology, Immunotherapy 64:1 | Wrong analysis |
| Symonds, E. L. and Cock, C. and Bampton, P. A. and Fraser, R. J. and Young, G. P. 2019 Use of Fecal Immunochemical Tests in an Elevated Risk Population: Predictors of Advanced Neoplasia. Gastroenterology 156 | Wrong population |
| Thompson, M. and Flashman, K. and Asiimwe, A. and O'Leary, D. and Parvaiz, A. and Senapati, A. 2014 A simple reliable clinical assessment of patients with bowel symptoms based on a 22-year prospective study of 29 005 outpatients of whom 1626 had bowel cancer. Colorectal Disease 2 | Wrong publication type |
| Thompson, M. R. and Perera, R. and Senapati, A. and Dodds, S. 2007 Predictive value of common symptom combinations in diagnosing colorectal cancer. British Journal of Surgery 94:10 | Wrong publication type |
| Thornton, M. J. and Herron, L. and Godber, I. and Downey, M. G. and Benyounes, H. 2019 Quantitative faecal immunochemical testing (qFIT) is a valuable tool for the triage of symptomatic colorectal patients. Colorectal Disease 21 | Wrong publication type |
| Thorsen, S. B. and Lundberg, M. and Villablanca, A. et al. 2013 Detection of serological biomarkers by proximity extension assay for detection of colorectal neoplasias in symptomatic individuals. Journal of Translational Medicine 11 | Wrong population |
| Tobi, M. and Rambus, M. A. and Antaki, F. 2016 Pre-colonoscopy fecal binding of ADNAB-9 anti-adenoma monoclonal is diagnostic for adenomas and | Wrong publication type |

| | |
|---|---|
| predictive for colorectal neoplasia risk. Gastroenterology 1 | |
| Tsai, W. S. and You, J. F. and Hung, H. Y. and Hsieh, P. S. et al. 2019 Novel Circulating Tumor Cell Assay for Detection of Colorectal Adenomas and Cancer. Clinical and Translational Gastroenterology 10:10 | Wrong population |
| Velamazan, R. and Hijos, G. and Saura, N. and Navarro, M. et al. 2020 The Use of a Quick and Easy Faecal Test as a Tool to Avoid Non-Pathological Colonoscopies and Prioritize High Risk Symptomatic Patients. Gastroenterology 158 | Wrong publication type |
| Vukobrat-Bijedic, Z. and Husic-Selimovic, A. and Bijedic, N. et al. 2014 Sensitivity of Symptomatology Versus Diagnostic Procedures and Concentration of CEA and CA19-9 in the Early Detection of Colorectal Cancer. Acta Informatica Medica 22:2 | Wrong study design |
| Wang, Z. and Shang, J. and Zhang, G. and Kong, L. and Zhang, F. and Guo, Y. and Dou, Y. and Lin, J. 2022 Evaluating the Clinical Performance of a Dual-Target Stool DNA Test for Colorectal Cancer Detection. Journal of Molecular Diagnostics 24:2 | Wrong population |
| Wanigasooriya, K. and Vayalapra, S. and Konda, N. and Karri, R. S. and Nightingale, P. and Beggs, A. and Ismail, T. 2019 Sensitivity and specificity of anaemia blood tests in detecting malignancy amongst patients referred via a 2-week wait suspected lower gastrointestinal cancer referral pathway. Colorectal disease 21 | Wrong publication type |
| Weller, D. and Thomas, D. and Hiller, J. and Woodward, A. and Edwards, J. 1994 Screening for colorectal cancer using an immunochemical test for faecal occult blood: results of the first 2 years of a South Australian programme. Australian & New Zealand Journal of Surgery 64:7 | Wrong population |
| Westwood, M. and Corro Ramos, I. and Lang, S. et al. 2017 Faecal immunochemical tests to triage patients with lower abdominal symptoms for suspected colorectal cancer referrals in primary care: a systematic review and cost-effectiveness analysis. Health Technology Assessment (Winchester, England) 21:33 | Wrong publication type |
| Westwood, M. and Lang, S. and Armstrong, N. et al. 2017 Faecal immunochemical tests (FIT) can help to rule out colorectal cancer in patients presenting in primary care with lower abdominal symptoms: a systematic review conducted to inform new NICE DG30 diagnostic guidance. BMC Medicine 15:1 | Wrong publication type |
| Widlak, M. and Neal, M. and Daulton, E. and Thomas, C. et al. 2018 Risk stratification of symptomatic patients using faecal biomarkers and urinary volatile organic compounds. Gut 67 | Wrong analysis |
| Widlak, M. M. and Neal, M. and Daulton, E. and Thomas, C. L. et al. 2018 Risk stratification of symptomatic patients suspected of colorectal cancer using faecal biomarkers (faecal immunochemical test and faecal calprotectin) and urinary volatile organic compounds. United European Gastroenterology Journal 6 | Wrong publication type |

| | |
|---|---|
| Widlak, M. M. and Thomas, C. L. and Thomas, M. G. et al. 2017 Undetectable faecal immunochemical test for haemoglobin excludes colorectal cancer in symptomatic patients: A prospective UK study. Gut 66 | Wrong publication type |
| Widlak, M. M. and Thomas, C. L. and Tomkins, C. and Smith, S. et al. 2016 Role of faecal biomarkers in detecting colorectal cancer and adenoma in symptomatic patients. United European Gastroenterology Journal 4 | Wrong publication type |
| Wilcox, B. and Croner, L. J. and Jones, J. J. and Benz, R. W. et al 2013 Plasma protein-based biomarker classifiers that discriminate patients with colon polyps or adenomas from those who do not as determined by colonoscopy. Gastroenterology 1 | Wrong publication type |
| Wilcox, B. E. and Benz, R. W. and Croner, L. J. and Dillon, R. and Jones, J. J. and Kao, A. and You, J. and Blume, J. E. 2016 The discovery and validation of blood plasma protein-based classifier panels for colorectal cancer and advanced adenoma using a combined mass spectrometry-and elisa-based workflow in studies including 1,605 patient samples. Gastroenterology 1 | Wrong publication type |
| Wilhelmsen, M. and Christensen, I. J. and Gawel, S. et al. 2015 Early detection of colorectal neoplasia: Combination of eight cancer-associated blood based protein biomarkers. Tumor Biology 36 | Wrong publication type |
| Wilhelmsen, M. and Rasmussen, L. and Davis, G. et al. 2016 Early detection of noeplasia: Combination of 17 cancer-associated blood-based protein biomarkers. Tumor Biology 37 | Wrong publication type |
| Willmann, L. and Alshiwanna, B. and Aston, D. and George, S. and Abi Hanna, D. and Bassan, M. S. and Koo, J. H. 2021 Risk Stratification Of Faecal Occult Blood Test Positive Patients To Low And High-Risk Groups: Can Patients Wait Beyond 30 Days For Their Colonoscopy? Gastroenterology 160 | Wrong population |
| Wilson, N. and Baker-Beal, L. and Kyaw, W. W. and Sonabend, R. and Logan, R. 2020 Real world experience of faecal immunochemical testing (FIT) in uk primary care to support the referral and diagnosis of colorectal cancer (CRC). United European Gastroenterology Journal 8 | Wrong publication type |
| Woodfield, G. and Belluomo, I. and Laponogov, I. and Veselkov, K. and Spanel, P. and Cross, A. J. and Hanna, G. B. 2021 Breath testing for colorectal polyps and cancer-the colorectal breath analysis1 study (COBRA1). Gut 70 | Wrong publication type |
| Xuan, Z. 2022 Clinical Value of Combined Detection of Mir-490-5p and Dicer1 in the Diagnosis of Rectal Cancer. Acta Medica Mediterranea 38 | Wrong population |
| Yang, Q. and Wang, S. and Huang, J. and Xia, C. and Jin, H. and Fan, Y. 2018 Serum miR-20a and miR-486 are potential biomarkers for discriminating colorectal neoplasia: A pilot study. Journal of Cancer Research and Therapeutics 14:7 | Wrong population |
| Yao, Y. and Ni, H. and Wang, X. and Xu, Q. and Zhang, J. and Jiang, L. and Wang, B. and Song, S. and Zhu, X. 2021 A New Biomarker of Fecal Bacteria for Non-Invasive Diagnosis of Colorectal | Wrong population |

| | |
|---|---|
| Cancer. Frontiers in Cellular and Infection Microbiology 11 | |
| Yin, B. and Mi, J. Y. and Zhai, H. L. and Zhao, B. Q. and Bi, K. X. 2021 An effective approach to the early diagnosis of colorectal cancer based on three-dimensional fluorescence spectra of human blood plasma. Journal of Pharmaceutical & Biomedical Analysis 193 | Wrong study design |
| Youssef, T. M. and Yousry, W. A. and Salama, M. M. and Ahmed, A. E. and Awaad, I. A. 2021 Evaluation of plasma M2-Pyruvate Kinase as a novel biomarker in the differentiation between Functional and Organic Colonic Disorders. QJM. Conference: 41st Annual International Ain Shams Medical Congress. Online. 114 | Wrong publication type |
| Zahorec, R. and Marek, V. and Waczulikova, I. and Veselovsky, T. and Palaj, J. and Keckes, S. and Durdik, S. 2021 Predictive model using hemoglobin, albumin, fibrinogen, and neutrophil-to-lymphocyte ratio to distinguish patients with colorectal cancer from those with benign adenoma. Neoplasma 68 | Wrong population |
| Brazer et al 1991 Using ordinal logistic regression to estimate the likelihood of colorectal neoplasia. J Clin Epidemiol. 44 | Wrong population |
| Colditz et al 2000 Harvard report on cancer prevention volume 4: Harvard cancer risk index. Risk index working group, Harvard venter for cancer prevention. Cancer causes control 11 | Wrong analysis |
| Imperiale et al 2003 Using risk for advanced proximal colonic neoplasia to tailor endoscopic screening for colorectal cancer. Ann Intern Med 139 | Wrong population |
| Driver JA, Gaziano JM, Gelber RP, et al. 2007 Development of a risk score for colorectal cancer in men. Am J Med 120 | Wrong population |
| Freedman AN, Slattery ML, Ballard-Barbash R, et al. 2009 Colorectal cancer risk prediction tool for white men and women without known susceptibility. J Clin Oncol 27 | Wrong population |
| Wei EK, Colditz GA, Giovannucci EL, et al. 2009Cumulative risk of colon cancer up to age 70 years by risk factor status using data from the Nurses' Health Study. Am J Epidemiol 170 | Wrong analysis |
| Kastrinos F, Allen JI, Stockwell DH, et al. 2009 Development and validation of a colon cancer risk assessment tool for patients undergoing colonoscopy. Am J Gastroenterol 104 | Wrong population |
| Ma E, Sasazuki S, Iwasaki M, et al. 2010 10-Year risk of colorectal cancer: development and validation of a prediction model in middle-aged Japanese men. Cancer Epidemiol. 34 | Wrong population |
| Park HW, Han S, Lee JS, et al. 2012 Risk stratification for advanced proximal colon neoplasm and individualized endoscopic screening for colorectal cancer by a risk-scoring model. Gastrointest Endosc 76 | Wrong population |
| Bellentani S, Baldoni P, Petrella S, et al. 1990 A simple score for the identification of patients at high risk of organic diseases of the colon in the family doctor consulting room. Fam Pract 7 | Wrong outcome |

| | |
|---|---|
| Fung et al, 2015 blood based protein biomarker panel for the detection of colorectal cancer. PLoS ONE | Wrong population |
| Tao et al 2012 comparison and combination of blood-based inflammatory markers with faecal occult blood tests for non-invasive colorectal cancer screening. British Journal of Cancer 106 | Wrong population |
| Birks et al. 2017 Evaluation of a prediction model for colorectal cancer: retrospective analysis of 2.5 million patient records. Cancer medicine 6:10 | Wrong population |
| Chen et al. 2021 A user-friendly objective prediction model in predicting colorectal cancer based on 234 044 Asian adults in a prospective cohort. ESMO 6:6 | Wrong population |
| Amirkhan et al 2018 Using recurrent neural networks to predict colorectal cancer among patients. I*EEE Symposium Series on Computational Intelligence (SSCI)* | Wrong population |

# PRISMA 2020 Checklist

| Section and Topic | Item # | Checklist item | Location where item is reported |
|---|---|---|---|
| **TITLE** | | | |
| Title | 1 | Identify the report as a systematic review. | Page 1 |
| **ABSTRACT** | | | |
| Abstract | 2 | See the PRISMA 2020 for Abstracts checklist. | Page 2 |
| **INTRODUCTION** | | | |
| Rationale | 3 | Describe the rationale for the review in the context of existing knowledge. | Page 3 |
| Objectives | 4 | Provide an explicit statement of the objective(s) or question(s) the review addresses. | Page 3 |
| **METHODS** | | | |
| Eligibility criteria | 5 | Specify the inclusion and exclusion criteria for the review and how studies were grouped for the syntheses. | Page 4 |
| Information sources | 6 | Specify all databases, registers, websites, organisations, reference lists and other sources searched or consulted to identify studies. Specify the date when each source was last searched or consulted. | Page 4 |
| Search strategy | 7 | Present the full search strategies for all databases, registers and websites, including any filters and limits used. | Supplement 2 |
| Selection process | 8 | Specify the methods used to decide whether a study met the inclusion criteria of the review, including how many reviewers screened each record and each report retrieved, whether they worked independently, and if applicable, details of automation tools used in the process. | Page 4 |
| Data collection process | 9 | Specify the methods used to collect data from reports, including how many reviewers collected data from each report, whether they worked independently, any processes for obtaining or confirming data from study investigators, and if applicable, details of automation tools used in the process. | Page 4 |
| Data items | 10a | List and define all outcomes for which data were sought. Specify whether all results that were compatible with each outcome domain in each study were sought (e.g. for all measures, time points, analyses), and if not, the methods used to decide which results to collect. | Page 4 |
| | 10b | List and define all other variables for which data were sought (e.g. participant and intervention characteristics, funding sources). Describe any assumptions made about any missing or unclear information. | Page 4 |
| Study risk of bias assessment | 11 | Specify the methods used to assess risk of bias in the included studies, including details of the tool(s) used, how many reviewers assessed each study and whether they worked independently, and if applicable, details of automation tools used in the process. | Page 4 |
| Effect measures | 12 | Specify for each outcome the effect measure(s) (e.g. risk ratio, mean difference) used in the synthesis or presentation of results. | Page 4/5 |
| Synthesis methods | 13a | Describe the processes used to decide which studies were eligible for each synthesis (e.g. tabulating the study intervention characteristics and comparing against the planned groups for each synthesis (item #5)). | Page 4/5 |
| | 13b | Describe any methods required to prepare the data for presentation or synthesis, such as handling of missing summary statistics, or data conversions. | Page 4/5 |
| | 13c | Describe any methods used to tabulate or visually display results of individual studies and syntheses. | Page 4/5 |
| | 13d | Describe any methods used to synthesize results and provide a rationale for the choice(s). If meta-analysis was performed, describe the model(s), method(s) to identify the presence and extent of statistical heterogeneity, and software package(s) used. | Page 4/5 |
| | 13e | Describe any methods used to explore possible causes of heterogeneity among study results (e.g. subgroup analysis, meta-regression). | NA |
| | 13f | Describe any sensitivity analyses conducted to assess robustness of the synthesized results. | NA |
| Reporting bias assessment | 14 | Describe any methods used to assess risk of bias due to missing results in a synthesis (arising from reporting biases). | NA |
| Certainty assessment | 15 | Describe any methods used to assess certainty (or confidence) in the body of evidence for an outcome. | NA |

# PRISMA 2020 Checklist

| Section and Topic | Item # | Checklist item | Location where item is reported |
|---|---|---|---|
| **RESULTS** | | | |
| Study selection | 16a | Describe the results of the search and selection process, from the number of records identified in the search to the number of studies included in the review, ideally using a flow diagram. | Page 5 |
| | 16b | Cite studies that might appear to meet the inclusion criteria, but which were excluded, and explain why they were excluded. | NA |
| Study characteristics | 17 | Cite each included study and present its characteristics. | Page 5 table 1 |
| Risk of bias in studies | 18 | Present assessments of risk of bias for each included study. | Figure 2 |
| Results of individual studies | 19 | For all outcomes, present, for each study: (a) summary statistics for each group (where appropriate) and (b) an effect estimate and its precision (e.g. confidence/credible interval), ideally using structured tables or plots. | Table 2/3 Figure 3/4 |
| Results of syntheses | 20a | For each synthesis, briefly summarise the characteristics and risk of bias among contributing studies. | Pages 5 - 10 |
| | 20b | Present results of all statistical syntheses conducted. If meta-analysis was done, present for each the summary estimate and its precision (e.g. confidence/credible interval) and measures of statistical heterogeneity. If comparing groups, describe the direction of the effect. | NA |
| | 20c | Present results of all investigations of possible causes of heterogeneity among study results. | NA |
| | 20d | Present results of all sensitivity analyses conducted to assess the robustness of the synthesized results. | NA |
| Reporting biases | 21 | Present assessments of risk of bias due to missing results (arising from reporting biases) for each synthesis assessed. | NA |
| Certainty of evidence | 22 | Present assessments of certainty (or confidence) in the body of evidence for each outcome assessed. | NA |
| **DISCUSSION** | | | |
| Discussion | 23a | Provide a general interpretation of the results in the context of other evidence. | Page 10/11 |
| | 23b | Discuss any limitations of the evidence included in the review. | Page 13 |
| | 23c | Discuss any limitations of the review processes used. | Page 13 |
| | 23d | Discuss implications of the results for practice, policy, and future research. | Page 11/12 |
| **OTHER INFORMATION** | | | |
| Registration and protocol | 24a | Provide registration information for the review, including register name and registration number, or state that the review was not registered. | Page 4 |
| | 24b | Indicate where the review protocol can be accessed, or state that a protocol was not prepared. | Page 4 |
| | 24c | Describe and explain any amendments to information provided at registration or in the protocol. | Page 4 |
| Support | 25 | Describe sources of financial or non-financial support for the review, and the role of the funders or sponsors in the review. | Page 14 |
| Competing interests | 26 | Declare any competing interests of review authors. | Page 14 |
| Availability of data, code and other materials | 27 | Report which of the following are publicly available and where they can be found: template data collection forms; data extracted from included studies; data used for all analyses; analytic code; any other materials used in the review. | Page 14 |

*From:* Page MJ, McKenzie JE, Bossuyt PM, Boutron I, Hoffmann TC, Mulrow CD, et al. The PRISMA 2020 statement: an updated guideline for reporting systematic reviews. BMJ 2021;372:n71. doi: 10.1136/bmj.n71

For more information, visit: http://www.prisma-statement.org/
